# Above the Energy Gap Law: Heavy Chalcogenide Substitution in NIR II-Emissive Diradicaloid Qubits

**DOI:** 10.1021/acscentsci.5c01001

**Published:** 2025-10-29

**Authors:** Lauren E. McNamara, Aimei Zhou, Sophie W. Anferov, David J. Gosztola, Matthew D. Krzyaniak, Michael R. Wasielewski, Lei Sun, Jan-Niklas Boyn, Richard D. Schaller, John S. Anderson

**Affiliations:** † Department of Chemistry, 2462 University of Chicago, Chicago, Illinois 60637, United States; ‡ Department of Chemistry, School of Science, 557712 Westlake University, Hangzhou, Zhejiang Province 310024, China; § Institute of Natural Sciences, Westlake Institute for Advanced Study, Hangzhou, Zhejiang Province 310024, China; ∥ Center for Nanoscale Materials, 1291 Argonne National Laboratory, 9700 S Cass Ave Lemont, 9700 S Cass Ave Lemont Illinois, 60439, United States; ⊥ Department of Chemistry, 3270 Northwestern University, Evanston, Illinois 60208, United States; # Key Laboratory for Quantum Materials of Zhejiang Province, Department of Physics, School of Science, 557712 Westlake University, Hangzhou, Zhejiang Province 310024, China; ∇ Department of Chemistry, 5635 University of Minnesota, Minneapolis, Minnesota 55455, United States

## Abstract

Near-infrared (NIR, 700–1700 nm)- and telecom (∼1260–1625 nm)-emissive molecules are good candidates for biological imaging and quantum sensing applications, respectively; however, bright low energy emission is rare due to exponentially increasing nonradiative decay rates in these regions, a phenomenon known as the energy gap law. Recent literature has emphasized the importance of minimizing skeletal modes to prevent increased nonradiative decay rates, but most organic lumiphores in these regions utilize large, conjugated scaffolds containing many C=C modes. Here we report a compact, telecom-emissive scaffold, tetrathiafulvalene-2,3,6,7-tetraselenate, or TTFts, that displays remarkable air, water, and acid stability, exhibits record quantum yields and brightness values, and retains quantum coherence under ambient conditions. These properties are enabled through methodical selenium substitution, which bathochromically shifts emission while shifting skeletal vibrations to lower energies. This new scaffold validates heavy heteroatom substitution strategies and establishes a new class of bright telecom emitters and robust qubits.

## Graphical abstract

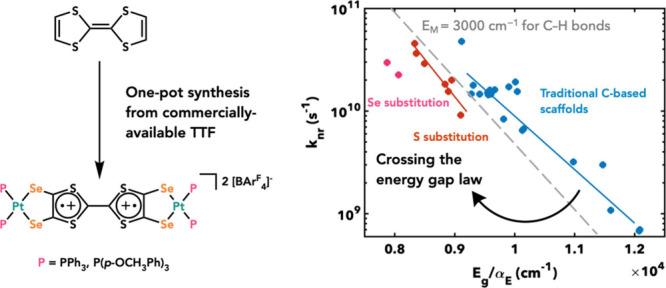

## Introduction

Molecules that emit in the NIR region are promising for several applications, including biomedical imaging, as they fall into the tissue transparent region where scattering and autofluorescence is minimized.^1–12^ Furthermore, molecules that emit deep into the NIR also fall into the telecom bands where attenuation is minimized in optical fibers, and they are thus ideal for communication and quantum information science applications.^13–15^ While many quantum dot-based systems can achieve bright emission in these low energy regions, they often suffer from increased toxicity and difficult excretion, targetability, and tunability.^16–19^ The organic-based lumiphores that operate at these wavelengths require large, conjugated scaffolds to shift absorption and emission into these low energy regions.^1,10,20–26^ These complex scaffolds introduce a multitude of vibrational modes, frequently with substantial ν_C–H_ and ν_C=C_ character, which have been theorized to contribute to the exponential increase in nonradiative decay rates as their energy gaps decrease, an empirical observation known as the energy gap law.^27–34^ Consequently, typical molecular dyes have extremely low photoluminescence quantum yields (PLQYs) as their transition energies decrease.^1^

Recently, our lab has reported a sulfur-based scaffold, tetrathiafulvalene-2,3,6,7-tetrathiolate, or TTFtt, that minimizes these deleterious vibrational modes through heteroatom substitution, allowing for bright NIR II (1000–1700 nm) emission from a much smaller scaffold.^35^ Furthermore, detailed photophysical measurements on TTFtt complexes conclusively demonstrate the importance of skeletal C=C modes, rather than C–Hs, in nonradiative decay in this region.^29^ These studies underscore the importance of skeletal mode management for enhanced PLQYs in the biomedically and technologically important NIR and telecom regions.

Heteroatom substitution, particularly with heavier chalcogenides, can bathochromically shift emission through compression of the π manifold while also minimizing and shifting deleterious skeletal vibrational modes to lower energies, enabling bright emission deeper into the NIR region.^36^ We therefore hypothesized that tetrathiafulvalene-2,3,6,7-tetraselenate (TTFts), a core with even heavier Se as the capping heteroatoms,^37^ should enable brighter and lower energy NIR emission if it could be stabilized in a doubly oxidized form through coordination to metal centers in analogy to TTFtt.

Here we report the first exploration of the coordination chemistry of TTFts. We successfully isolate Pt complexes of TTFts with thorough characterization of their structure, composition, and physical properties. Two electron oxidation of the TTFts core in these complexes enables bright 1300–1400 nm organic-based emission and diradical character on a compact and modular scaffold. Photophysical and vibrational studies reveal that the heavier Se atoms shift vibrational modes to lower energies, leading to record PLQYs, brightness values, and nonradiative decay rates for organic-based monomer emission >1300 nm. This bright NIR emission and diradical character also make TTFts-based molecules compelling candidates for applications in quantum information science. Excitingly, room temperature coherence is maintained in solution and transient EPR measurements suggest optical spin polarization. Additionally, stability in water and acid is maintained over several hours, ideal for biological quantum sensing applications. These studies demonstrate that heavy heteroatom substitution is an effective strategy to move past the traditional limitations of the energy gap law and paves the way for robust, telecom-emissive organic qubits.

## Results and Discussion

### Synthesis and Structural Characterization

We generated TTFts^4–^ using a one-pot synthesis reported by Williams in 1989 where tetrathiafulvalene (TTF) is deprotonated by 4 equiv of lithium diisopropylamide (LDA) and then stirred with 4 equiv of Se powder.^38^ This in situ generated tetraanion was then reacted with 2 equiv of P_2_PtCl_2_ (where P = triphenylphosphine for **1** and tris­(*p*-methoxyphenyl)­phosphine for **2**, see Figure 1a), followed by in situ oxidation with 2 equiv of [Fc^BzO^]­[BAr^F^_4_] (where Fc^BzO^ = benzoylferrocenium and BAr^F^_4_ = tetrakis­(3,5-bis­(trifluoromethyl)­phenyl)­borate) to obtain the final dicationic complexes **1** and **2**. Both capping phosphines were chosen to directly compare to previously synthesized TTFtt analogs, which have shown electronic structure tunability through changes in phosphine donation.^29,39^ Furthermore, Pt was specifically chosen due to the remarkable stability seen in analogous Pt-capped TTFtt dicationic systems.^29,35,39^ Pt helps shift the +1/+2 redox couple in these systems toward less oxidizing potentials which inhibits reduction and thus allows for stability in a variety of solvents even in air.

**Figure 1:**
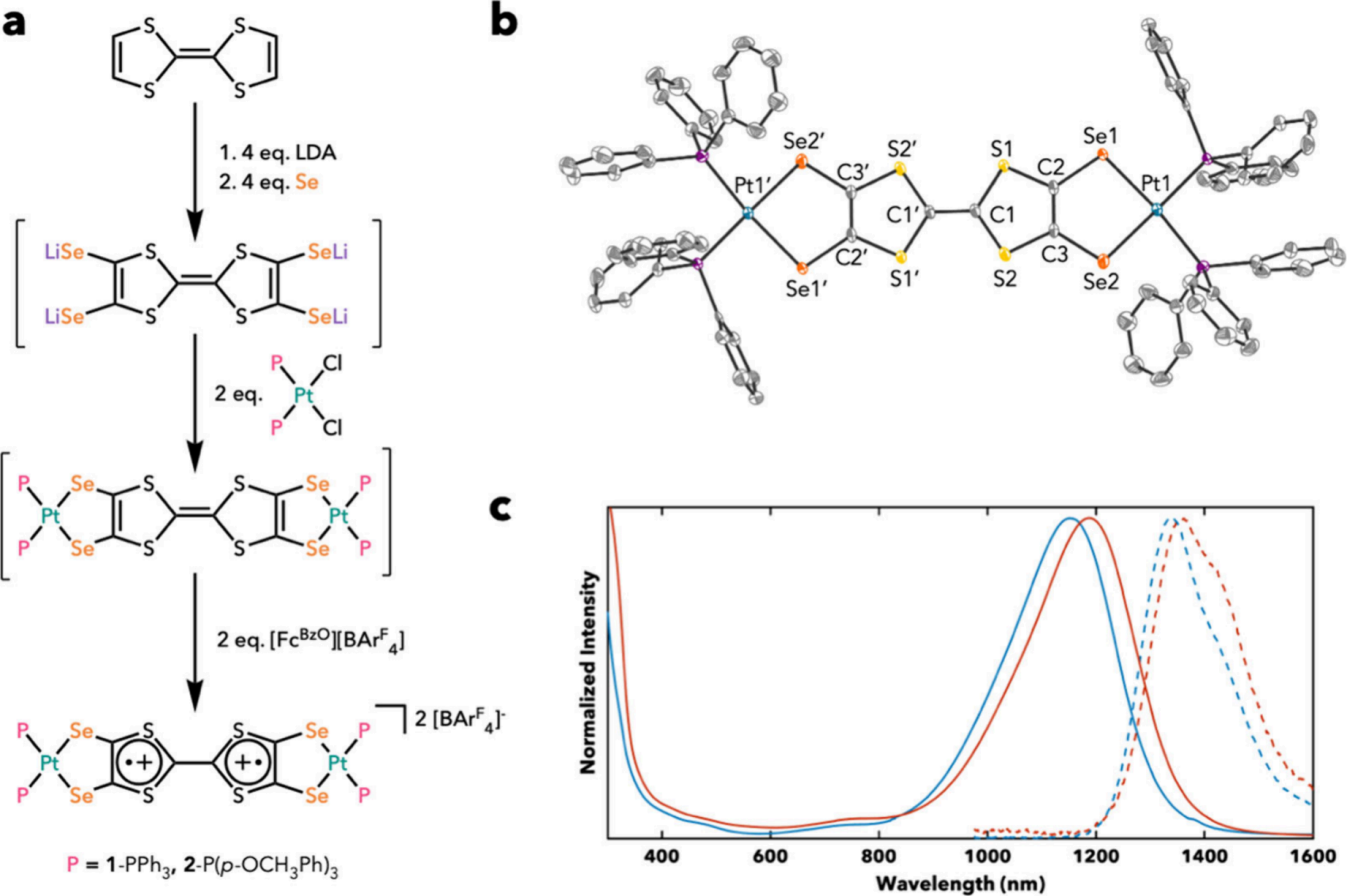
(a) Synthetic scheme for complexes **1** and **2**. (b) Single-crystal structure of **1**, with hydrogens, solvent, and counteranions omitted for clarity. Thermal ellipsoids shown at 50%. (c) Absorption (solid) and emission (dashed) spectra for **1** and **2** shown in blue and red, respectively.

Crystals of **1** suitable for single-crystal X-ray diffraction (SXRD) were grown by layering a dichloromethane (DCM) solution with petroleum ether and diffusing overnight at −35 °C. The structure of **1** confirms the synthesis of the desired TTFts dicationic complex. We note that this is the first structure and isolation of a TTFts complex with any transition metal and thus provides an opportunity to study the coordination chemistry of this chalcogenide rich motif. The SXRD structure of **1** reveals slight out-of-plane bending at the Se sites which is atypical for TTFtt analogs.^29,35,39^ Additionally, the central C1–C1’ (see Figure 1b) distance in **1** is 1.431(9) Å, longer than the C1–C1′ distance of 1.404(7) Å reported in the analogous TTFtt compound; however, the C–S bonds in the TTF core are shorter in TTFts than in TTFtt.^29^

NMR analyses also confirm the synthesis and purity of the desired complexes **1** and **2**, with Se satellites present in the ^195^Pt and ^31^P spectra. Evans method measurements confirm these complexes also display moderate diradical character, consistent with the properties of the related TTFtt complexes, with moments around 1.25 μ_B_ for both **1** and **2** (Figures S7 and S8).^29,35,39,40^

### Photophysical Properties

In DCM, both **1** and **2** display significant shifts (∼55 nm bathochromic, see Table 1 and Figure 1c) from their TTFtt counterparts, absorbing at 1154 and 1190 nm, respectively.^29,39^ These NIR II absorptions are also remarkably intense, with extinction coefficients (ε) of 105,400 M^–1^cm^–1^ for **1** and 92,800 M^–1^cm^–1^ for **2**. Upon excitation, these complexes emit far into the NIR II region at 1340 and 1362 nm, an ∼ 80 nm bathochromic shift from the TTFtt analogs for **1** and **2**, respectively.^29,39^ Notably, this emission is the brightest in this region (>1300 nm) for molecular organic lumiphores, with PLQYs of 0.065% for **1** and 0.028% (see eq 1) for **2**. When accounting for overall brightness (ε × PLQY), these dyes are also record-setting for organic monomers emitting >1300 nm with values of 6,850 and 2,600 M^–1^ cm^–1^.^24^ Furthermore, these dyes are promising for tail emission imaging in the telecom C band, where attenuation is lowest.^13–15^ At 1550 nm, emission intensity is still at 15.3% and 22.9% of the emission maxima for **1** and **2**, respectively.

**Table 1:** Photophysical Parameters and Lifetimes across TTFts Complexes in DCM at 298 K^a^

	PLQY (%)	λ_Abs,exp_ (nm)	λ_Em,exp_ (nm)	λ_Abs,th,S_ (nm)	λ_Em,th,S_ (nm)	λ_Abs,th,T_ (nm)	τ_TA,S_ (ps)	τ_TA,T_ (ps)	*k*_nr_ (10^10^ s^–1^)
**1**	0.065	1154	1340	1111	1214	1387	44.4 ± 5.3	996.3 ± 514	2.2 ± 0.3
**2**	0.028	1190	1362	1138	1246	1391	33.7 ± 1.4	795.9 ± 172	3.0 ± 0.1
**1-TTFtt** ^29^	0.070	1100	1266	1055	1160	1141	34.5 ± 7.4	^a^	2.9 ± 0.6
**2-TTFtt** ^39^	0.041	1130	1280	1141	1190	1143	22.0 ± 4.4	648.2 ± 913	4.5 ± 0.9

a Triplet decay not fit; decay longer than instrument resolution.^29^ **1-TTFtt** and **2-TTFtt** indicate the same capping metals, ligands, and counteranions as **1** and **2**, respectively, but with a TTFtt core instead of TTFts. “T” and “S” mark triplet and singlet transitions, “exp” indicates experimentally measured, and “th” indicates theoretically predicted. “TA” marks lifetimes derived from transient absorption measurements. See Experimental Section and Supporting Information for equations used to calculate the nonradiative rates.

We conducted ultrafast NIR transient absorption (TA) on both compounds in DCM to determine the excited state lifetimes and whether reduced nonradiative rates enabled this record brightness. The resultant TA spectra show similar, albeit bathochromically-shifted, features to the TTFtt analogs.^29,39^ A ground state bleach (GSB) appears at ∼1100–1150 nm, an excited state absorption (ESA) is present at ∼1220–1250 nm, and stimulated emission (SE) is seen at ∼1350–1390 nm across both complexes (see Figure 2a, Figure S15). This excited state, previously assigned as *S*_1_ in related TTFtt systems, has a lifetime of 44.4 ps for **1** and 33.7 ps in **2** (see Table 1, Figure 2b and Figure 4a).^29,39^ The singlet state assignment is supported by variable excitation and oxygen experiments (see Figures S94–S96). These singlet lifetimes are comparable to TTFtt-based dyes that emit 100 nm bluer, indicative of a major decrease in nonradiative decay rates in these bathochromically-shifted, TTFts-based systems.^39^ Notably, the GSB shifts, and a new ESA grows in around 20 ps, indicative of intersystem crossing (ISC) to a nearby triplet excited state, *T*_2_ (see below, Figure 4a). These putative triplet signals persist to ∼2.5 ns for both complexes, around the time window of our measurement. Shortwave infrared (SWIR) TA measurements were conducted to better capture triplet spectral content and enable fitting of the triplet decay, which yielded approximate decay lifetimes of ∼996 and 796 ps for **1** and **2**, respectively.

**Figure 2:**
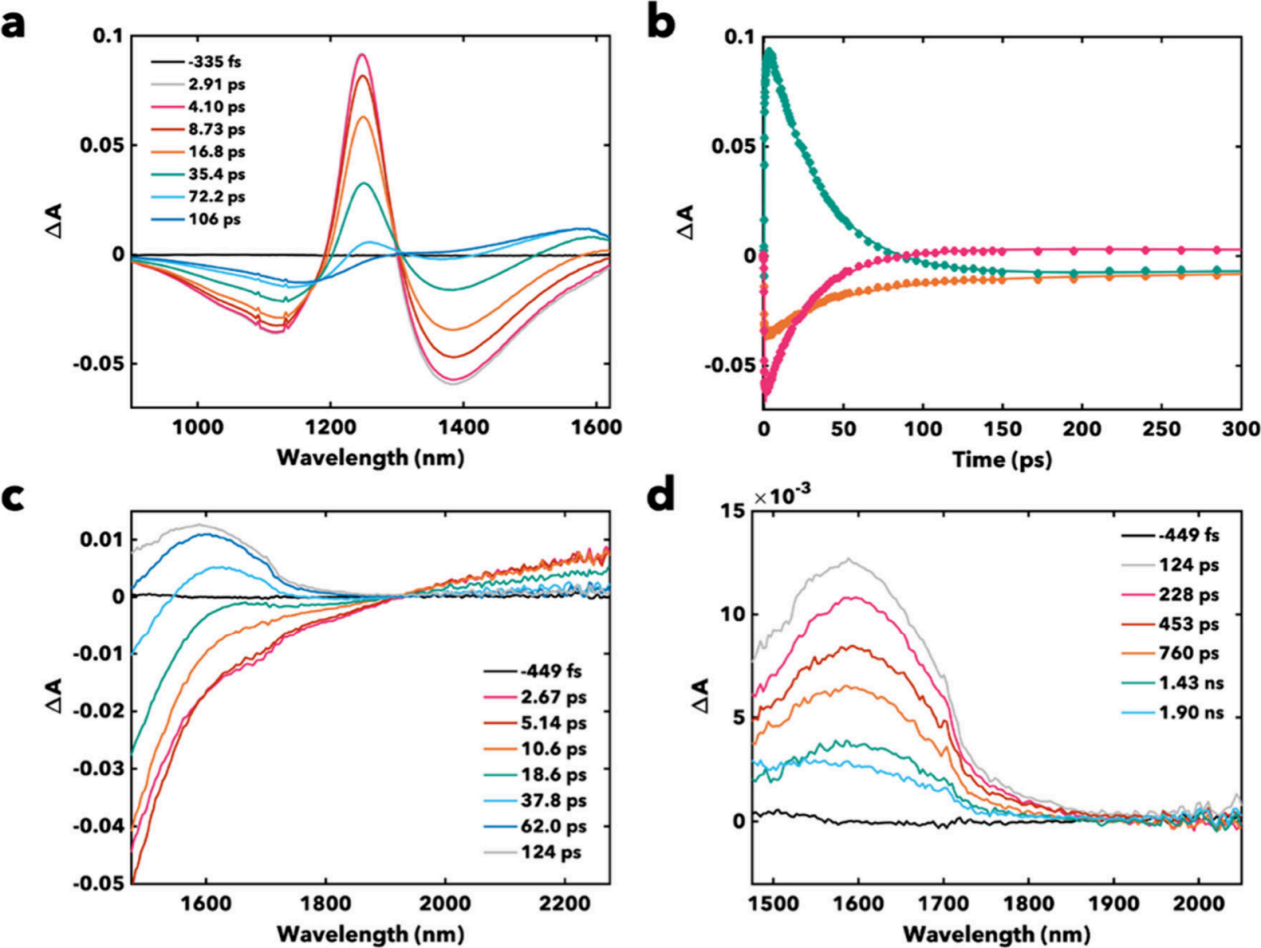
(a) NIR TA spectra of **2** in DCM at 298 K. (b) Transient kinetics of **2** in DCM at 298 K, corresponding to the three main GSB (orange), ESA (teal), and SE (magenta) features. (c) SWIR TA spectra of **2** in DCM at 298 K, showing decay of initial excited state (*S*_1_). (d) SWIR TA spectra of **2** in DCM at 298 K, showing decay of second excited state (putatively *T*_2_).

SWIR measurements capturing the red edge of the SE feature were also consistent with the previously acquired singlet lifetimes, and they showed an additional ESA corresponding to *S*_1_ that spectrally extends past the SWIR detection limit of ∼2300 nm (see Figures 2c and 2d). We have previously determined that the *S*_1_ TA lifetimes in related TTFtt systems are near identical with the time-resolved photoluminescence excited-state lifetimes acquired by time-correlated single-photon counting (TCSPC) measurements, so here we use the TA lifetimes (given the improved temporal resolution of TA) to calculate nonradiative rates for these new TTFts-based systems (see Table 1).^35^

Both the nonradiative decay rates, *k*_nr_, of **1** and **2** (see Table 1) are dramatically slower when compared to the nonradiative decay rates of analogous reported phosphine-capped PtTTFtt complexes, even beyond the already slower nonradiative decay rates in these species compared to most other molecular organic-based fluorophores in their respective regions (see Figure 3a).^28,29,35,39^ This improvement is best demonstrated by extrapolating the organic and TTFtt trend lines to the energy gaps of **1** and **2** in Figure 3a. Such extrapolation would result in predicted *k*_nr_ values near or above the ∼10^11^ s^–1^ limit shown in Figure 3a. Comparing these three groups of molecular, organic-based, π–π emitters clearly emphasizes the improvement in nonradiative decay rates in this class of NIR lumiphores. This slower nonradiative decay supports that these new TTFts-based systems constitute promising platforms for OLED and bioimaging applications.^28,29,35,39,41^ With this new class of NIR-emitting molecules, nonradiative decay rates have finally pushed far past the traditional molecular, organic-based limitations predicted by the energy gap law and the previously reported nonradiative decay rates for other organic NIR emitters.^27,28^

**Figure 3:**
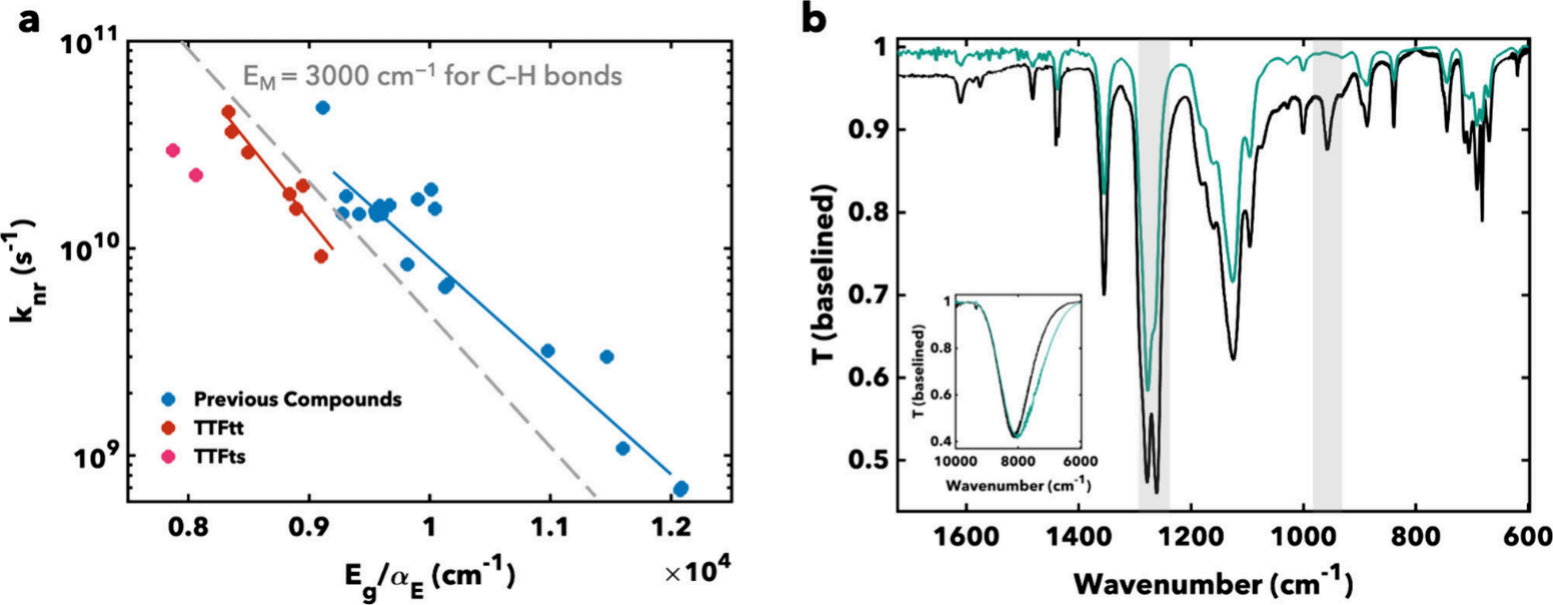
(a) Comparison of nonradiative decay rates across previously reported organic lumiphores,^22^ phosphine-capped TTFtt analogs,^23,29,33^ and **1** and **2**. Dashed line indicates predicted C–H mode limitation on nonradiative decay rates. (b) Comparison of IR spectra of **1** (teal) and TTFtt (black) analog,^23^ normalized to the NIR feature, dropcast onto KBr plates from a DCM solution. Gray boxes highlight changes between the spectra. Inset: IR spectra showing bathochromic shift of NIR feature.

### Vibrational Analysis

Given the remarkably slowed nonradiative decay rates in this new class of lumiphores, we investigated changes in vibrational structure upon Se substitution. We have previously hypothesized that skeletal vibrations play an important role in mediating nonradiative decay in NIR lumiphores. Thus, we were interested in changes between the vibrational spectra of TTFts and TTFtt analogs.^29,39^

Fourier-transform infrared spectroscopy (FTIR) measurements display the expected bathochromic shift in electronic absorption in TTFts compared to TTFtt in the NIR region (Figure 3b, inset). Of note, we normalized this region for ease of comparison, but due to a drop-off in the detector response past 8000 cm^–1^ this shift is more evident in the trailing absorption. More interestingly, two major changes are apparent at low energies. First, the features at ∼ 1260 and 1278 cm^–1^ (predicted by DFT to correspond to linear combinations of coupled core and aryl C=C modes, see Supplementary Videos 1–4) display major intensity changes relative to each other moving from TTFtt to TTFts. Furthermore, a feature around 955 cm^–1^ in TTFtt, previously assigned as a C–S stretch, is no longer present in the FTIR spectrum of TTFts presumably due to red-shifting outside of the instrument spectral window.^29^

Modulation of vibrational profiles by inclusion of heavier Se atoms is further supported by DFT frequency calculations, which predict shifting of the C–S modes and significant decreases in the intensity of the core vibrations moving from TTFtt to TTFts (). The calculated magnitude of the vibrational intensity decrease is even larger for the shift from TTFtt to TTFts than for perdeuteration of the TTFtt analog of **1**, **1-TTFtt**, where experimental studies demonstrated that nonradiative decay was suppressed and PL lifetimes roughly doubled upon deuteration.^29^ All of this experimental and theory data supports that the much lower energy vibrations due to the larger mass of Se, along with the shift in vibrational symmetry and secondary vibrational environment (i.e., C=C vibrations attached to S and Se vs all S), enable bright emission by increasing the quanta needed to reach degeneracy with electronic transitions.

### Electronic Structure

Given that previous bathochromically-shifted TTFtt complexes exhibit small T–S gaps, we experimentally and theoretically investigated the T–S gaps in these new TTFts systems as well.^35,39^ Experimentally, we conducted variable-temperature (VT) photoluminescence (PL), TCSPC, and TA measurements to determine the ground states of **1** and **2**. PL and TCSPC were measured in both 1:1 dibromomethane:toluene (DBrM:toluene) and inert polymer to rule out rigidification effects.

The low temperature PL spectra of **1** and **2** in 1:1 DBrM:toluene show resolved vibronic features spaced ∼275 cm^–1^ apart. This spacing is notably smaller than that seen in their TTFtt counterparts.^29^ The shifting of these coupled vibrational modes upon deuteration was calculated to be behind the doubling of lifetime, and consequently these coupled skeletal vibrations were predicted to be responsible for the energy gap law behavior seen in this region. Thus, the shifting of this vibronic spacing to lower energies could be responsible for the improved performance of these TTFts-based emitters in these low energy regions.

Upon cooling, both analogs show increased PL intensity (roughly an order of magnitude increase in PLQY at 5 K compared to 298 K) and longer lifetimes, which has been observed before in their TTFtt counterparts and tentatively attributed to decreased ISC from *S*_1_ to *T*_2_, although it could potentially also indicate depopulation of vibrational modes. However, **2** shows a decrease in both PL intensity and lifetime (see Figure 4c) upon cooling below ∼10 K in both polymer and DBrM:toluene, a behavior which is not observed in **1**.

**Figure 4:**
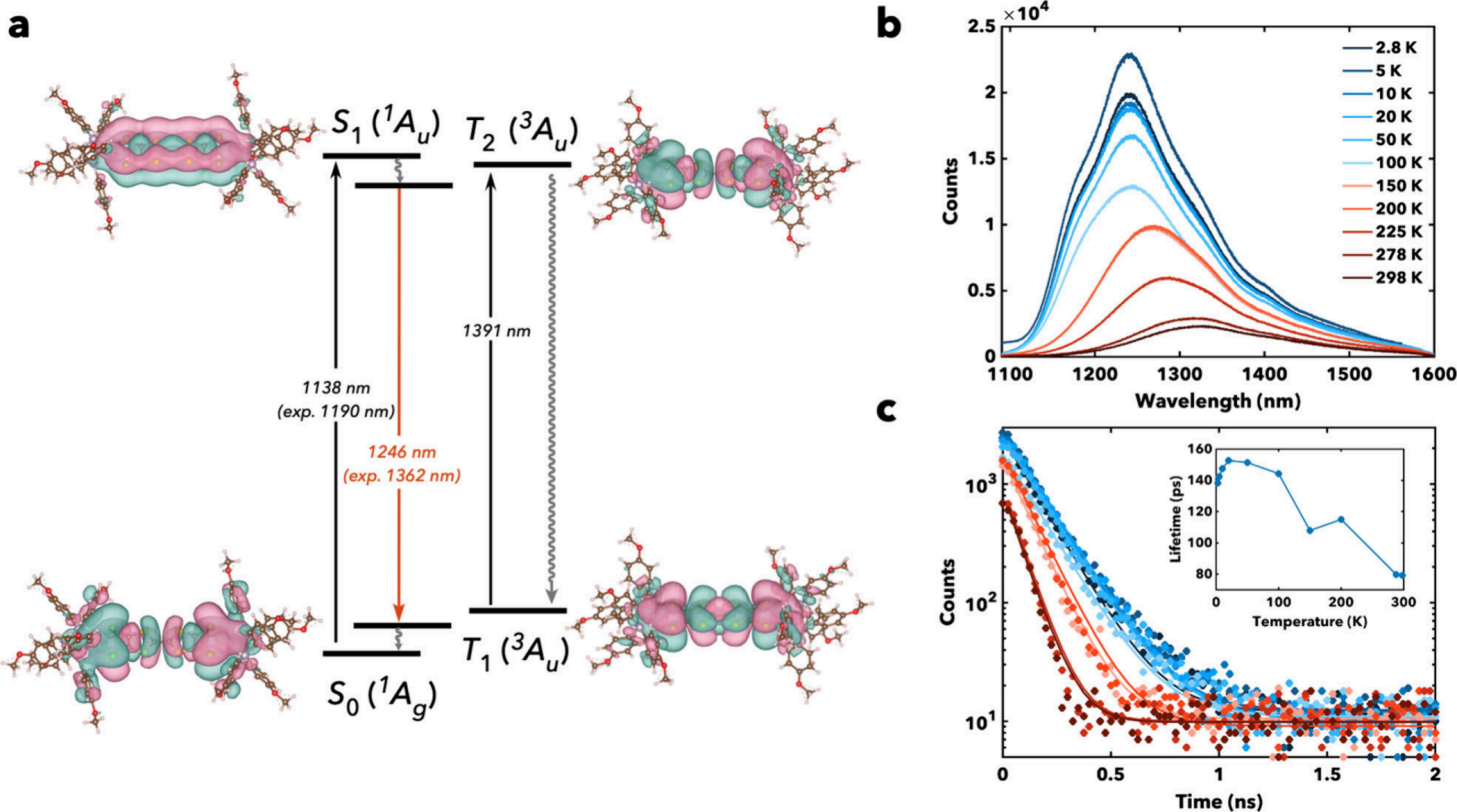
(a) Jablonski diagram of **2**, showing the natural transition orbitals (NTOs) obtained via TD-DFT simulations for both the singlet and triplet manifolds.^42^ Wavelengths shown are TD-DFT predicted values, except where noted. (b) Variable-temperature PL of **2** in 1:1 DBrM:toluene. (c) Variable-temperature lifetime measurements of **2** in 1:1 DBrM:toluene. Inset: lifetime fits of **2** across temperatures in 1:1 DBrM:toluene.

Both TCSPC and TA show this lifetime decrease at low temperatures in **2**, and while a decrease in PL intensity could be indicative of a triplet ground state, the lifetime decrease in addition to the PL decrease may more reasonably correspond to a negative *T*_2_–*S*_1_ gap in which ISC is favored at lower temperatures. Theory supports this hypothesis, as calculations predict that **2** has a slightly smaller *T*_2_–*S*_1_ gap that could explain the low temperature drop in lifetimes and PL. We have previously shown that the *T*_1_–*S*_0_ gap in TTFtt complexes can be modulated by capping ligands, but these data suggest that it may be possible to also modulate excited state *T*_2_–*S*_1_ gaps with capping ligand identity. This offers another potential handle for electronic structure design and optimization for quantum sensing applications.

DFT predicts that complexes **1** and **2** have a singlet ground state, with **2** displaying a small *T*_1_–*S*_0_ gap of 1.75 kcal/mol (see ). Additionally, both **1** and **2** are predicted to have near-identical diradical character of ∼0.72 by CASSCF­(4,4) (see ). Both complexes are also predicted to have extremely high oscillator strength (∼1.04, see ) absorptions around 1100 nm from the singlet ground state, with the OMe substitution resulting in a bathochromic shift of 27 nm, consistent with the donation trend seen in triarylphosphine-capped TTFtt analogs.^39^ Absorption from the triplet state is predicted to be bathochromically-shifted by ∼270 nm compared to the singlet ground state, with absorptions around 1390 nm and slightly reduced oscillator strengths. Emission from the singlet is predicted to lie ∼100 nm bathochromically-shifted from the ground state absorption, and emission oscillator strengths are comparable to those corresponding to absorption. Relaxation from the upper triplet (*T*_2_, see Figure 4a) is a symmetry-forbidden u to u transition which may account for its nonemissive nature. Notably, theory also suggests Se substitution does not result in a dramatic increase in spin–orbit coupling (SOC), as the transition from *S*_0_ to *T*_1_ in **1** is predicted to have an oscillator strength on the order of ∼10^–6^. This low SOC has potentially important implications for the application of these molecules as qubits.

### Qubit Viability

We have previously established that TTFtt-based systems are promising qubits, operable under ambient conditions and in solution.^43^ Coupling the stability and telecom emission of these systems with ambient, solution-phase coherence would lay the groundwork for optically addressable molecular qubits for use in biological and communications applications.

We conducted continuous-wave electron paramagnetic resonance (CW EPR) characterization on 0.1 mM 1:1 DCM:toluene (v:v) solutions of compounds **1**, **2**, and **2-TTFtt** at room temperature. Spectra of **1** and **2** exhibit axial signals, indicating both molecules are in the slow motion regime due to their large sizes and high molecular weights.^44^ The spectrum of **1** shows relatively narrow peaks, which can be fit with anisotropic *g*-factors (*g*_⊥_ = 2.0071, *g*_∥_ = 2.0057) and a relatively small zero-field splitting (ZFS, |*D*| = 16 MHz) with a tumbling correlation time of 16 ns (Figure 5a). The EPR features of **2** are too weak and broad to fit (Figure S97a). The significant line broadening of **2** indicates faster spin relaxation and decoherence. In comparison, the spectrum of **2-TTFtt** shows an isotropic peak with *g* = 2.0028, close to the free-electron value, well-resolved hyperfine splitting with *A*_Pt_ = 10.1 MHz and *A*_P_ = 5.2 MHz, and unresolved ZFS (Figure S97b). Overall, Se substitution increases both molecular weight and spin–orbit coupling, giving rise to larger *g*-factors, slower molecular tumbling, and observable ZFS. The pronounced triplet character of **1** and **2** and their optical properties suggests potential viability for optical detection of magnetic resonance (ODMR).

**Figure 5:**
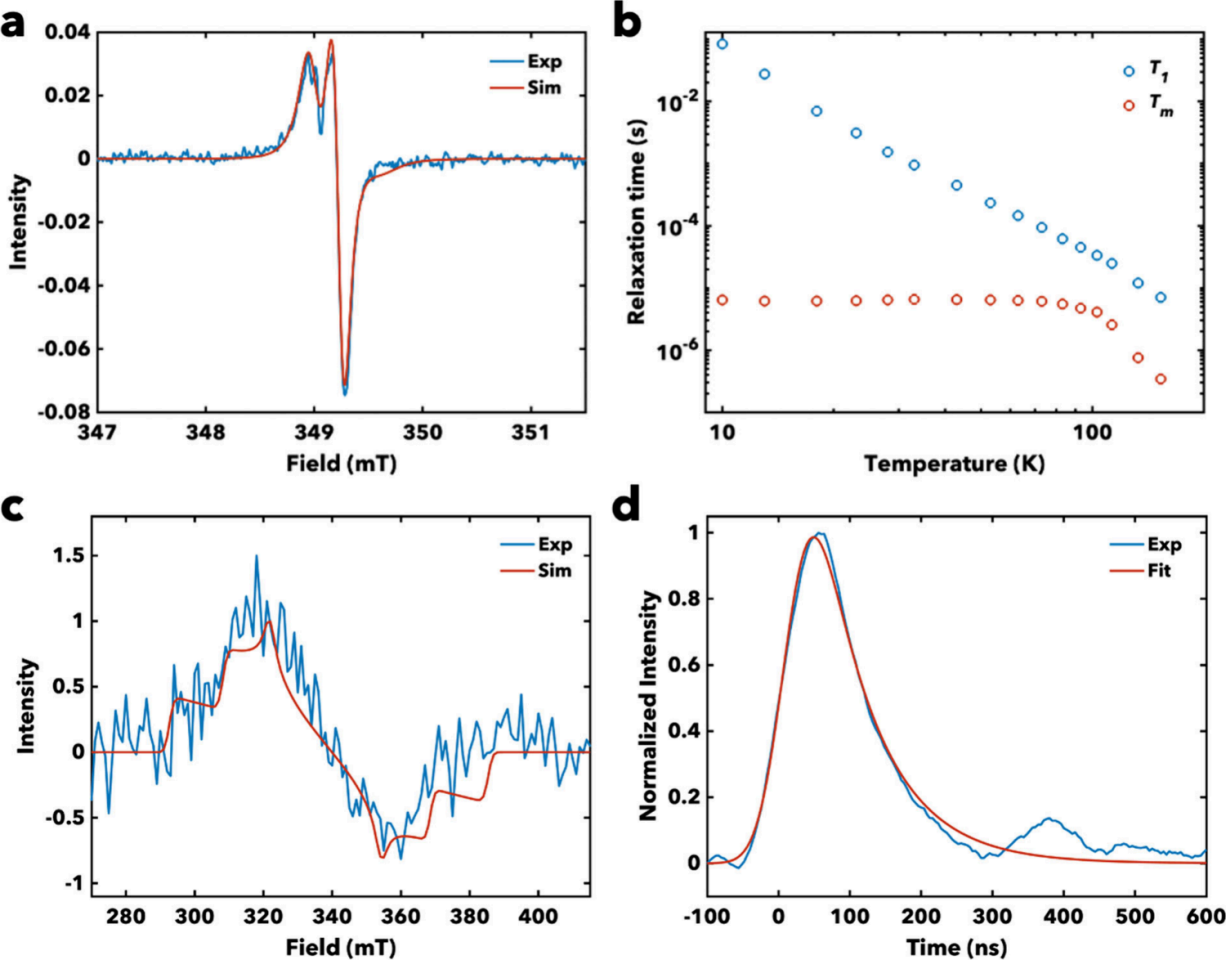
(a) Continuous-wave EPR of **1** in 1:1 DCM:toluene at 295 K. (b) Spin–lattice relaxation (*T*_1_) and phase memory (*T*_m_) times of 0.1 mM solution of **2** in 1:1 DCM:toluene across various temperatures. (c) Transient EPR spectrum of **2** in 1:1 DBrM:toluene at 85 K. (d) Transient kinetics of **2** in 1:1 DBrM:toluene at 85 K.

We then examined the spin dynamics of **1**, **2**, and **2-TTFtt** with pulse EPR spectroscopy. A nutation experiment on **1** at 295 K generated Rabi oscillations that show coherent addressability of this molecule via resonant microwave radiation (Figure S99). Inversion recovery and Hahn echo decay measurements revealed a spin–lattice relaxation time of *T*_1_ = 916 ns and a phase memory time of *T*_m_ = 158 ns (Figure S100–101, see eqs 2 and 3). Both times are slightly shorter than previously reported values for the TTFtt-based qubit PtTTFtt,^43^ yet they are sufficiently long for quantum sensing protocols, e.g. electron spin echo envelope modulation (ESEEM).^45^ These results confirm the qubit nature of **1** in solution at room temperature.

**2** and **2-TTFtt** displayed no pulse EPR signal at room temperature, so we performed variable-temperature pulse EPR measurements to assess their qubit viability (Figure S102). At low temperatures, **2** behaves similarly to PtTTFtt (Figure 5b).^43^ At 10 K, its *T*_1_ is remarkably long, on the order of 100 ms, and *T*_m_ is approximately 8 μs (see eqs 2 and 4). As the temperature rises to 100 K, *T*_1_ decreases by more than 3 orders of magnitude while *T*_m_ remains relatively constant. Around 100 K, *T*_m_ drastically drops and becomes unmeasurable above 153 K, which is attributed to increased solvent motion generating magnetic noise. *T*_1_ declines nearly exponentially with increasing temperatures (*T*_1_ ∝ *T* ^–3.26^) from 10 to 153 K, showing the dominant role of the Raman process in spin relaxation (Figure S103). The coherence is not recovered above the melting point of the solvent, consistent with the broad CW EPR features of **2**. Considering the structural differences between **1** and **2**, we attribute the shorter room temperature *T*_m_ of **2** to the methoxy groups, which might introduce additional motion and slower molecular tumbling while increasing molecular weight. Both effects may facilitate spin decoherence, leading to unmeasurable *T*_m_ at room temperature. In addition, **2-TTFtt** shows marginal pulse EPR signals even at low temperatures (Figure S104), and we only managed to measure it at 30 K. Its *T*_1_ and *T*_m_ are 840 and 6.1 μs, respectively, both of which are shorter than the values of **2** (*T*_1_ = 1.05 ms and *T*_m_ = 6.5 μs at 30 K) (Figures S105 and S106). Hence, the substitution of S for Se does not significantly alter qubit performance.

Lastly, we conducted transient EPR (trEPR) measurements in 1:1 DBrM:toluene to confirm ISC, characterize the spin properties of the *T*_2_ state, and determine the lifetime of *T*_2_ at 85 K. Following photoexcitation of **2** with an 8 ns, 900 nm, laser pulse, a broad a-a-a-e-e-e (where a = enhanced absorption and e = emission) triplet EPR spectrum is observed centered around *g* = 2 (see Figure 5c and 5d). Simulation of the spectrum yields the ZFS parameters of |*D*| = 1.3 GHz and *E*/*D* = 0.1. This value of |*D*| would correspond to a mean distance of 3.4 Å under the point dipole approximation which agrees well with the distance between the central rings.

### Stability

Given that the TTFts moiety has never been coordinated to metals before, we were interested in probing the stability of these Pt-capped TTFts diradicaloids. Redox stability was initially examined with cyclic voltammetry (CV), where two reversible features can be assigned as TTFts^2–/3–^ and TTFts^3–/4–^ redox couples in analogy to the more thoroughly studied redox properties of TTFtt (see Figures S12 and S13).^39,49^ The potentials for these couples range from −0.091 V to −0.238 V vs ferrocene/ferrocenium in DCM for **1** and **2**. Similar to previously established trends in triarylphosphine-capped TTFtt analogs, the increase in donation of the *para* substituent dramatically shifts the redox couple to more negative potentials, ideal for imaging in a biologically reducing environment. These comparatively reducing potentials also suggest suitable stability for these complexes in a variety of solvent environments.

Correspondingly, complexes **1** and **2** show good solution-phase stability across several solvents in air (Figures S76–S87). Similar to the TTFtt analogs, strong negative solvatochromic behavior is observed, which is promising for ratiometric imaging applications (Figures S88 and S89).^39^ While a general negative solvatochromic trend is observed, overlapping solvent vibrational overtones also result in a changing emission profile across solvents which makes a quantitative comparison difficult. Furthermore, these compounds are also soluble and show no degradation over the course of 14 h in 1:40 DMSO:water mixtures and emission can be detected in aqueous solutions. Remarkably, these compounds are also completely soluble, stable, and bright in glacial acetic acid, demonstrating the necessary robustness for possible use in gastrointestinal imaging.^46^ Thus, these complexes are remarkably robust candidates for a variety of applications, in addition to their excellent qubit and photophysical properties.

## Conclusions

We have synthesized the first coordination complexes of the Se-based linker TTFts and have further isolated the previously unreported TTFts-based dicationic diradicaloid motif. The addition of Se atoms to a TTF core allows for near 1400 nm organic-based emission, with record PLQYs, brightness, and nonradiative decay rates for organic monomers emitting beyond 1300 nm. These values far exceed those of traditional organic NIR lumiphores in this region and are a significant improvement from previously reported TTFtt-based systems, pushing emission further into the telecom bands while decreasing nonradiative decay. These record values, along with the remarkable stability of these systems, are especially promising for the design of high-efficiency telecom OLEDs and bright quantum sensors. Additionally, TTFts-based qubits exhibit similarly excellent spin coherence properties to TTFtt-based systems, opening the door for room temperature, telecom-operable optically addressable qubits.

## Experimental Section

### General Synthetic Methods

All linker synthesis, metalation, and oxidation procedures were performed under inert conditions (dry N_2_) in a MBraun UNIlab glovebox. Elemental analyses (C, H, N) were conducted by Midwest Microlabs. All solvents used were dried, purged with N_2_ on a Pure Process Technology solvent system, and subsequently filtered through activated alumina and stored over 4 Å molecular sieves. Pt­(*p*-OCH_3_Ph)_2_Cl_2_,^47^ Pt­(PPh_3_)_2_Cl_2_,^48^ **2-TTFtt**,^39^ and [Fc^BzO^]­[BAr^F^_4_],^49^ were prepared according to literature procedures. All other chemicals and reagents were purchased from commercial sources and used as received.

### Single-Crystal X-ray Diffraction (SXRD)

SXRD data was collected at 100 K on a Bruker D8 VENTURE diffractometer equipped with a microfocus Mo-target X-ray tube (λ = 0.71073 Å) and a PHOTON 100 CMOS detector. A single crystal was mounted on a cryo-loop and transferred into the diffractometer nitrogen stream before collection. Data reduction and integration were performed with the Bruker APEX3 ^50^ software package (Bruker AXS, version 2015.5–2, 2015). Data was scaled and corrected for absorption effects using the multiscan procedure as implemented in SADABS (Bruker AXS, version 2014/54, 2015). The crystal structure was solved by SHELXT^51^ (version 2014/55) and refined by a full-matrix least-squares procedure using OLEX26 ^52^ (XL refinement program version 2018/17). Disorder was modeled with common restraints. We note some A-level alerts, resulting from extraneous electron density near the heavy atoms Pt and Se. However, the bond lengths and geometrical aspects of the core scaffold are still sufficiently high resolution for interpretation. Deposition number 2415439 contains the supplementary crystallographic data for this paper. These data can be obtained free of charge via the joint Cambridge Crystallographic Data Centre (CCDC) and Fachinformationszentrum Karlsruhe Access Structures service.

### Cyclic Voltammetry

Cyclic voltammetry measurements were performed using a silver wire pseudoreference, glassy carbon working electrode, and platinum wire counter electrode. Each voltammogram was referenced to an internal standard (Fc^+^/Fc). All measurements were acquired using a BASi Epsilon potentiostat and analyzed using the BASi Epsilon software version 1.40.67NT.

### FTIR Spectroscopy

All IR measurements were performed by dropcasting DCM solutions onto KBr plates. Each spectrum was acquired on a Bruker Tensor II; both background subtractions and baseline corrections were applied for each complex using the OPUS software.

### NMR Spectroscopy

^1^H, ^31^P, and ^195^Pt NMR spectra were acquired on Bruker DRX 400 and 500 spectrometers. Residual solvent peaks were referenced for all ^1^H NMR measurements and 85% phosphoric acid and sodium hexachloroplatinate were used as references for ^31^P and ^195^Pt NMR measurements, respectively. Evans method measurements were conducted in CD_2_Cl_2_ with a capillary insert of 95/5 v/v% CD_2_Cl_2_/DCM. Pascal’s constants were used to correct for the diamagnetic contribution.^53^

### UV–Vis–NIR

UV–vis–NIR measurements were performed using a Shimadzu UV-3600 Plus dual beam spectrophotometer and a Thermo Scientific Evolution 300 spectrometer and analyzed using VisionPro software.

### Photoluminescence Spectroscopy

Room temperature emission spectra were acquired on a Horiba Scientific PTI QuantaMaster fluorometer. Low temperature emission spectra were recorded by loading samples in 1:1 DBrM:toluene into 0.5 mm cuvettes, sealing with vacuum grease, and cooling in a Janis cryostat.

### Transient Absorption Spectroscopy

NIR transient absorption spectroscopy was performed using a 5 kHz amplified titanium:sapphire laser with a 120 fs laser pulsewidth. A portion of the 800 nm fundamental output was focused into a sapphire crystal to produce near-infrared continuum probe pulses that were mechanically delayed. Pump pulses were tuned to 900 nm using an optical parametric amplifier, and a mechanical chopper reduced the repetition rate to 2.5 kHz. Samples were excited with 0.2 mW of pump power.

SWIR transient absorption with a shortwave infrared probe was performed using a 35 fs amplified Ti:sapphire laser operating at 2 kHz. The 2 mJ output was beamsplit equally and directed to two optical parametric amplifiers (OPAs). One OPA was tuned to produce a signal wavelength of 1200 nm that was used as the pump beam after mechanically reducing the repetition rate to 1 kHz and mechanically delayed. The probe beam was produced using the idler output of the second OPA that was tuned to 2400 nm and focused into a sapphire crystal to produce a shortwave infrared continuum. This beam was dispersed in a grating spectrometer and detected on a single-shot basis using a red-extended InGaAs array detector.

### Lifetime Measurements

Time-correlated single photon counting was performed on the samples using a 975 nm diode laser with a 60 ps pulsewidth operating at 20 MHz. Collected PL was dispersed using a 0.3 m spectrograph and detected with a superconducting nanowire single photon detector and multichannel scaler with 25 ps bin width. Of note, the instrument response function (IRF) falls around ∼50 ps.

### Photoluminescence Quantum Yield Determination

The samples and references, in 1 cm quartz fluorescence cuvettes (Starna), were normalized to optical densities at 900 nm using UV–vis–NIR measurements. All samples were kept at or below 0.1 OD at 900 nm to minimize reabsorption effects. Three different ODs were tested per sample, forming a gradient upon plotting against the integrated photoluminescence. The individual quantum yields were then calculated using this gradient and the reference method (reference is PtdppeTTFtt, PLQY = 0.136% in DCM at 298 K^35^). The following equation was used:

1 $${PLQY}_{s}={PLQY}_{r}(\frac{m_{s}}{m_{r}}{)\left(\frac{n_{s}}{n_{r}}\right)}^{2}$$

Here *m* is the gradient of the OD vs integrated PL plot and *n* is the refractive index of the solvent. r and s refer to the reference and the sample, respectively.

### Nonradiative Rate Determination

Both the experimentally determined quantum yield values, ϕ, and the TA singlet lifetimes were utilized to determine the radiative and nonradiative rates through the expression ϕ = *k*_r_/(*k*_r_ + *k*_nr_), where the denominator, *k*_r_ + *k*_nr_, is the reciprocal of the singlet lifetime, τ_TA,S_.

### Continuous Wave (CW) Electron Paramagnetic Resonance (EPR) Spectroscopy

The CW EPR spectra of **1**, **2**, and **2-TTFtt** were acquired using a Bruker E500 spectrometer operating at X-band (9.6 GHz) frequencies at the Instrumentation and Service Center for Molecular Sciences, Westlake University. The magnetic field was calibrated for accuracy by a standard BDPA radical sample, which revealed +0.351 G correction. The modulation amplitude was set to for **2** and **2-TTFtt**, and 0.2 G for **1**. The microwave power was 0.1 mW. The solution was prepared under an N_2_ atmosphere by dissolving crystalline material in 1:1 DCM: toluene and diluting to 1 × 10^–4^ mol/L. The sample was flame-sealed in a quartz tube and the CW EPR spectrum was recorded at room temperature (295 K). The CW EPR spectrum was fitted using the chili function in EasySpin in MATLAB R2023b with a *S* = 1 spin.^54^ Fitting for **1** revealed *g*_⊥_ = 2.00705, *g*_∥_ = 2.00569, *D* = 15.961 MHz, and 0.05578 mT of Gaussian broadening. Fitting for **2-TTFtt** revealed isotropic *g* = 2.00278, *A*_Pt_ = 10.1 MHz, *A*_P_ = 5.2 MHz, *D* = 0, and 0.18 mT of Gaussian broadening.

### Room-Temperature Pulse EPR Spectroscopy

The room-temperature echo-detected field swept (EDFS), inversion recovery, and Hahn echo decay experiments of **1** were acquired with a CIQTEK EPR100 spectrometer operating at X-band (9.6 GHz) frequencies equipped with an X-band pulsed ENDOR dielectric resonator EN4202DR at the Instrumentation and Service Center for Molecular Sciences, Westlake University. Samples were prepared under inert conditions by dissolving crystalline **1** in 1:1 DCM:toluene and diluting to 1 × 10^–4^ mol/L. The sample was flame-sealed in a quartz tube.

For **1**, π/2 and π pulses were applied with lengths of 16 and 32 ns, respectively. The pulse lengths were optimized with a three-pulse nutation sequence (nutation pulse – *T* – π/2 – τ – π – τ – echo) where the length of the nutation pulse was varied, and delays were set as τ = 120 ns and T = 400 ns. The relationship between the intensity of the echo and the length of the nutation pulse exhibits a nutation pattern. The microwave attenuation was tuned such that the corresponding pulse lengths of the local maxima and minima are integer multiples of 32 ns. Pulses were phased by applying a two-pulse Hahn echo sequence (π/2 – τ – π – τ – echo) at the resonant magnetic field and adjusting the phase to maximize the sum of square of the real component and minimize the sum of square of the imaginary component of the Hahn echo. All pulse EPR data were further phased by maximizing the sum of square of their real component and minimizing the sum of square of their imaginary component. All experiments were conducted with the shot repetition time (SRT) being longer than five times of *T*_1_. When echo integration was applied, approximately the top 2/3 of the echo was integrated to reduce the influence of noise.

The echo-detected field sweep (EDFS) spectrum was collected with a two-pulse Hahn echo sequence (π/2 – τ – π – τ – echo) with 4.0 mT scan width, 120 ns delay time, 100 shots per point, 512 data points, and at 296 K for **1**. π/2 and π pulses were applied with lengths of 16 and 32 ns, respectively. Long (selective) pulses were used to avoid overexcitation of the spin packets to achieve the proper line shape. Two-step phase cycling was employed with pulse phases of (+*x*, +*x*) and (−*x*, +*x*) to cancel background drift and the defense pulse. Integration of the echo was plotted against the magnetic field strength, giving an EDFS spectrum.

The spin–lattice relaxation time (*T*_1_) was characterized by an inversion recovery sequence (π – *T* – π/2 – τ – π – τ – echo) with 512 data points, at the magnetic field with the maximum EDFS intensity. τ was 120 ns, T was 400 ns, beginning and increment of T was 40 ns, and shots per point was 100. Four-step phase cycling was employed with pulse phases of (+*x*, −*x*, +*x*) (+*x*, +*x*, +*x*) (−*x*, −*x*, +*x*) and (−*x*, +*x*, +*x*) to cancel background drift, unwanted echoes, and the defense pulse. Integration of the echo was plotted against the delay time, *T*, giving the inversion recovery curve. It was fitted by a monoexponential decay function

2 $$I=ae^{-T/T_{1}}+I_{0}$$

where *I* is echo intensity, *a* is a prefactor, and *I*_0_ accounts for baseline drift.

The phase memory time (*T*_m_) was characterized by a two-pulse Hahn echo sequence (π/2 – τ – π – τ – echo) with 512 data points, at the magnetic field with the maximum EDFS intensity. τ was 120 ns and shots per point was 100. Two-step phase cycling was employed with pulse phases of (+*x*, +*x*) and (−*x*, +*x*) to cancel background drift and the defense pulse. Integration of the echo was plotted against twice of the delay time, 2τ, giving an echo decay curve. The Hahn echo decay curves collected were fitted by a monoexponential decay function:

3 $$I=ae^{-2\tau /T_{m}}+I_{0}$$

The nutation experiment was conducted with a three-pulse sequence (nutation pulse – *T* – π/2 – τ – π – τ – echo) with 100 shots per point, 512 data points, at the magnetic field with the maximum EDFS intensity. The length of the nutation pulse started at 6 ns and was incremented with 2 ns per step. τ was fixed at 120 ns. Four-step phase cycling was employed with pulse phases of (+*x*, −*x*, +*x*) (+*x*, +*x*, +*x*) (−*x*, −*x*, +*x*) and (−*x*, +*x*, +*x*) to cancel background drift, unwanted echoes, and the defense pulse. Integration of the echo was plotted against the length of the nutation pulse, giving a nutation curve.

### Low Temperature Pulse EPR Spectroscopy

The variable-temperature inversion recovery and Hahn echo decay experiments of **2** and **2-TTFtt** were acquired with a CIQTEK EPR100 spectrometer operating at X-band (9.6 GHz) frequencies at the Instrumentation and Service Center for Molecular Sciences, Westlake University. Samples were prepared under inert conditions by dissolving crystalline **2** or **2-TTFtt** in 1:1 DCM:toluene mixture (volume ratio) and diluting to 1 × 10^–4^ mmol/L. This mixture is a glassy solvent, so that molecular aggregation can be avoided when the solution is frozen. Samples were flame-sealed in a quartz tube.

The cryogenic temperature was controlled by a closed-loop helium cryostat EPR-VTS-L-D1-PWC and a Lakeshore 336 temperature controller. Samples were cooled at 10 K for at least 2 h before spin dynamics experiments. The thermal equilibrium was monitored by the *T*_1_ of the sample: it was established when two consecutive inversion recovery experiments, separated by 5 min, revealed *T*_1_ values with the difference less than 1%. Such thermal stabilization process typically took 20–30 min, after which *T*_1_ and *T*_m_ were acquired. We collected *T*_1_ and *T*_m_ from 10 to 133 K of **2** in one experimental session.

Inversion recovery and Hahn echo experiments were conducted with the same methods discussed in Room-Temperature Pulse EPR Spectroscopy, except with τ = 200 ns. The shot repetition time (SRT) was adjusted to be longer than five times of *T*_1_ at each temperature. The inversion recovery curves were all fitted with a monoexponential decay function (eq 2).

The Hahn echo decay curves were fitted by a stretched exponential decay function

4 $$I=ae^{-{\left(\frac{2\tau }{T_{m}}\right)}^{q}}+I_{0}$$

where *I* is echo intensity, *a* is a prefactor, *q* is the stretch factor, and *I*_0_ accounts for baseline drift.

### Transient EPR Measurements

The X-band time-resolved EPR measurements were performed on a Bruker Elexsys E580 X EPR spectrometer equipped with a split ring resonator (Bruker ER4118X-MS5). The temperature was kept at 85 K using an Oxford Instruments CF935 continuous-flow cryostat cooled with liquid nitrogen and controlled with an Oxford Instruments MercuryiTC. The sample was photoexcited with 7 ns, 2 mJ/pulse, 900 nm pulses generated by an optical parametric oscillator (GWU Basi-scan), pumped with the output of a frequency-tripled Nd:YAG laser (Spectra-Physics Quanta-Ray Lab 150). The laser light was coupled into the resonator via a fiber optic (Thorlabs FT1000UMT) and collimator placed outside the cryostat. The transient magnetization was acquired in quadrature under CW irradiation (∼5 mW). The EPR spectra were processed in MATLAB using home written scripts and the simulation package EasySpin v6.0.6.^54^

## Supplementary Material







## Notes

The Supporting Information is available free of charge on the ACS Publications Web site. (PDF) Supplementary Videos. The Supporting Information is available free of charge at https://pubs.acs.org/doi/10.1021/acscentsci.5c01001.

Additional experimental details and crystal coordinates (PDF)
Crystallographic data for **1** (CIF)
Calculated vibrations of **1** corresponding to 1134 cm^–1^ vibrational mode and 1349 cm^–1^ vibrational mode (.gif) and of **2** corresponding to 1336 cm^–1^ vibrational mode and 1352 cm^–1^ vibrational mode (ZIP)
