# Supplementary material for: Above the Energy Gap Law: Heavy Chalcogenide Substitution in NIR II-Emissive Diradicaloid Qubits
Source: ACS Cent Sci. 2025 Oct 29;11(11):2257–67. doi: 10.1021/acscentsci.5c01001 (PMC12670300; doi:10.1021/acscentsci.5c01001)
Supplement: Supplementary file 1 [file oc5c01001_si_001.pdf]

Supporting Information for

Above the energy gap law: Heavy chalcogenide substitution in NIR II-emissive diradicaloid qubits

Lauren E. McNamara,[1] Aimei Zhou,[2,3] Sophie W. Anferov,[1] David J. Gosztola,[4] Matthew D. Krzyaniak,[5] Michael R. Wasielewski,[5] Lei Sun,[2,3,6] Jan-Niklas Boyn,[7] Richard D. Schaller,[4,5] and John S. Anderson[1,]*

[1]Department of Chemistry, University of Chicago, Chicago, IL, USA
[2]Department of Chemistry, School of Science, Westlake University, Hangzhou, Zhejiang Province, China
[3]Institute of Natural Sciences, Westlake Institute for Advanced Study, Hangzhou, Zhejiang Province, China
[4]Center for Nanoscale Materials, Argonne National Laboratory, Argonne, IL, USA
[5]Department of Chemistry, Northwestern University, Evanston, IL, USA
[6]Key Laboratory for Quantum Materials of Zhejiang Province, Department of Physics, School of Science, Westlake University, Hangzhou, Zhejiang Province, China
[7]Department of Chemistry, University of Minnesota, Minneapolis, MN, United States

*Correspondence to: jsanderson@uchicago.edu

Table of Contents

General synthetic methods *6*

Characterization and analysis methods *6*

Synthetic procedures **10**

Synthesis of [{$(PPh_3)_2Pt$}$_2$TTFts][BAr^F_4]$_2$ (1) **10**

Synthesis of [{$(P(p\text{-}OCH_3Ph)_3)_2Pt$}$_2$TTFts][$BAr^F_4$]$_2$ (2) **11**

NMR spectra *12*

1. ^{1}H NMR spectra **12**

Figure S1. ^{1}H NMR spectrum of **1** in CD_2Cl_2. 12
Figure S2. ^{1}H NMR spectrum of **2** in CD_2Cl_2. 12

2. ^{31}P NMR spectra **13**

Figure S3. ^{31}P{^{1}H} NMR spectrum of **1** in CD_2Cl_2. 13
Figure S4. ^{31}P{^{1}H} NMR spectrum of **2** in CD_2Cl_2. 13

3. ^{195}Pt NMR spectra **14**

Figure S5. ^{195}Pt{^{1}H} NMR spectrum of **1** in CD_2Cl_2. 14
Figure S6. ^{195}Pt{^{1}H} NMR spectrum of **2** in CD_2Cl_2. 14

4. Evans method measurements **15**

Figure S7. Evans method ^{1}H NMR spectrum of a 6.3 mM solution of **1** in CD_2Cl_2. Magnetic moment: 1.251 μ_B. Inset: Zoomed-in spectrum to show solvent shift. 15
Figure S8. Evans method ^{1}H NMR spectrum of a 5.2 mM solution of **2** in CD_2Cl_2. Magnetic moment: 1.260 μ_B. Inset: Zoomed-in spectrum to show solvent shift. 15

Infrared spectra *16*

Figure S9. Infrared transmittance spectrum of **1** showing NIR window. Sample prepared by dropcasting a DCM solution. 16
Figure S10. Infrared transmittance spectrum of **2** with NIR window. Sample prepared by dropcasting a DCM solution. 16
Figure S11. Infrared transmittance spectrum of **2** (teal) compared with **2-TTFtt** (black) with normalized NIR window (lower) and skeletal vibrational shifts (upper). Samples prepared by dropcasting a DCM solution.... 17

Electrochemical measurements *18*

Figure S12. Cyclic voltammogram of 1 mM **1** in 0.1 M [TBA][PF_6] in DCM. Scan rate: 0.1 V/s. 18
Figure S13. Cyclic voltammogram of 1mM **2** in 0.1 M [TBA][PF_6] in DCM. Scan rate: 0.1 V/s. Magenta trace shows smaller scan window, teal trace shows larger scan window. 18

Optical spectroscopy *19*

1. Transient absorption spectra and lifetime determination **19**

Figure S14. Growth of NIR transient absorption spectra of **1** in DCM at 298 K. 19
Figure S15. Decay of NIR transient absorption spectra of **1** in DCM at 298 K. 19
Figure S16. Decay of NIR transient absorption spectra of **1** in DCM at 298 K, at long timepoints. 20
Figure S17. NIR transient kinetics of **1** in DCM at 298 K. 20
Figure S18. Growth of NIR transient absorption spectra of **1** in CH_2Br_2 (DBrM) at 298 K. 21
Figure S19. Decay of NIR transient absorption spectra of **1** in DBrM at 298 K. 21
Figure S20. Decay of NIR transient absorption spectra of **1** in DBrM at 298 K, at long timepoints. 22
Figure S21. NIR transient kinetics of **1** in DBrM at 298 K. 22
Figure S22. Growth of NIR transient absorption spectra of **2** in DCM at 298 K. 23
Figure S23. Decay of NIR transient absorption spectra of **2** in DCM at 298 K, at long timepoints. 23
Figure S24. Growth of NIR transient absorption spectra of **2** in DBrM at 298 K. 24
Figure S25. Decay of NIR transient absorption spectra of **2** in DBrM at 298 K. 24

Figure S26. Decay of NIR transient absorption spectra of **2** in DBrM at 298 K, at long timepoints. 25
Figure S27. NIR transient kinetics of **2** in DBrM at 298 K. 25
Figure S28. Growth of SWIR transient absorption spectra of **1** in DCM at 298 K. 26
Figure S29. Decay of SWIR transient absorption spectra of **1** in DCM at 298 K. 26
Figure S30. Decay of SWIR transient absorption spectra of **1** in DCM at 298 K, at long timepoints. 27
Figure S31. SWIR transient kinetics of **1** in DCM at 298 K. 27
Figure S32. Growth of SWIR transient absorption spectra of **2** in DCM at 298 K. 28
Figure S33. SWIR transient kinetics of **2** in DCM at 298 K. 28
Figure S34. Growth of NIR transient absorption spectra of **1** in 1:1 DBrM:toluene at 298 K. 29
Figure S35. Decay of NIR transient absorption spectra of **1** in 1:1 DBrM:toluene at 298 K. 29
Figure S36. Decay of NIR transient absorption spectra of **1** in 1:1 DBrM:toluene at 298 K, at long timepoints. 30
Figure S37. NIR transient kinetics of **1** in 1:1 DBrM:toluene at 298 K. 30
Figure S38. Growth of NIR transient absorption spectra of **1** in 1:1 DBrM:toluene at 100 K. 31
Figure S39. Decay of NIR transient absorption spectra of **1** in 1:1 DBrM:toluene at 100 K. 31
Figure S40. Decay of NIR transient absorption spectra of **1** in 1:1 DBrM:toluene at 100 K, at long timepoints. 32
Figure S41. NIR transient kinetics of **1** in 1:1 DBrM:toluene at 100 K. 32
Figure S42. Growth of NIR transient absorption spectra of **1** in 1:1 DBrM:toluene at 2.9 K. 33
Figure S43. Decay of NIR transient absorption spectra of **1** in 1:1 DBrM:toluene at 2.9 K. 33
Figure S44. Decay of NIR transient absorption spectra of **1** in 1:1 DBrM:toluene at 2.9 K, at long timepoints. 34
Figure S45. NIR transient kinetics of **1** in 1:1 DBrM:toluene at 2.9 K. 34
Figure S46. Growth of NIR transient absorption spectra of **2** in 1:1 DBrM:toluene at 298 K. 35
Figure S47. Decay of NIR transient absorption spectra of **2** in 1:1 DBrM:toluene at 298 K. 35
Figure S48. Decay of NIR transient absorption spectra of **2** in 1:1 DBrM:toluene at 298 K, at long timepoints. 36
Figure S49. NIR transient kinetics of **2** in 1:1 DBrM:toluene at 298 K. 36
Figure S50. Growth of NIR transient absorption spectra of **2** in 1:1 DBrM:toluene at 195 K. 37
Figure S51. Decay of NIR transient absorption spectra of **2** in 1:1 DBrM:toluene at 195 K. 37
Figure S52. Decay of NIR transient absorption spectra of **2** in 1:1 DBrM:toluene at 195 K, at long timepoints. 38
Figure S53. NIR transient kinetics of **2** in 1:1 DBrM:toluene at 195 K. 38
Figure S54. Growth of NIR transient absorption spectra of **2** in 1:1 DBrM:toluene at 100 K. 39
Figure S55. Decay of NIR transient absorption spectra of **2** in 1:1 DBrM:toluene at 100 K. 39
Figure S56. Decay of NIR transient absorption spectra of **2** in 1:1 DBrM:toluene at 100 K, at long timepoints. 40
Figure S57. NIR transient kinetics of **2** in 1:1 DBrM:toluene at 100 K. 40
Figure S58. Growth of NIR transient absorption spectra of **2** in 1:1 DBrM:toluene at 50 K. 41
Figure S59. Decay of NIR transient absorption spectra of **2** in 1:1 DBrM:toluene at 50 K. 41
Figure S60. Decay of NIR transient absorption spectra of **2** in 1:1 DBrM:toluene at 50 K, at long timepoints. 42
Figure S61. NIR transient kinetics of **2** in 1:1 DBrM:toluene at 50 K. 42
Figure S62. Growth of NIR transient absorption spectra of **2** in 1:1 DBrM:toluene at 10 K. 43
Figure S63. Decay of NIR transient absorption spectra of **2** in 1:1 DBrM:toluene at 10 K. 43
Figure S64. Decay of NIR transient absorption spectra of **2** in 1:1 DBrM:toluene at 10 K, at long timepoints. 44
Figure S65. NIR transient kinetics of **2** in 1:1 DBrM:toluene at 10 K. 44
Figure S66. Growth of NIR transient absorption spectra of **2** in 1:1 DBrM:toluene at 2.9 K. 45
Figure S67. Decay of NIR transient absorption spectra of **2** in 1:1 DBrM:toluene at 2.9 K. 45
Figure S68. Decay of NIR transient absorption spectra of **2** in 1:1 DBrM:toluene at 2.9 K, at long timepoints. 46
Figure S69. NIR transient kinetics of **2** in 1:1 DBrM:toluene at 2.9 K. 46

2. TCSPC lifetime determination 47

Figure S70. Photoluminescence decay (upper) and fit lifetimes (lower) of **1** in 1:1 DBrM:toluene from 298 K (red) to 2.8 K (blue)........ 47
Figure S71. Photoluminescence decay (upper) and fit lifetimes (lower) of **1** in polymer from 298 K (red) to 2.8 K (blue). Polymer used is (poly(butyl methacrylate-co-isobutyl methacrylate)........ 48
Figure S72. Photoluminescence decay (upper) and fit lifetimes (lower) of **2** in polymer from 298 K (red) to 2.8 K (blue)........ 49

3. Variable temperature photoluminescence........ 49
Figure S73. Photoluminescence across temperatures of **1** in polymer........ 50
Figure S74. Photoluminescence across temperatures of **1** in 1:1 DBrM:toluene........ 50
Figure S75. Photoluminescence across temperatures of **2** in polymer........ 51

4. Stability and solvatochromism experiments........ 51
Figure S76. Stability of **1** in MeCN in air, at 298 K........ 51
Figure S77. Stability of **1** in acetone in air, at 298 K........ 52
Figure S78. Stability of **1** in THF in air, at 298 K........ 52
Figure S79. Stability of **1** in DCM in air, at 298 K........ 53
Figure S80. Stability of **1** in 1:1 DBrM:toluene in air, at 298 K........ 53
Figure S81. Stability of **2** in 1:40 DMSO:water, in air, at 298 K........ 54
Figure S82. Stability of **2** in THF in air, at 298 K........ 54
Figure S83. Stability of **2** in MeCN in air, at 298 K........ 55
Figure S84. Stability of **2** in acetone in air, at 298 K........ 55
Figure S85. Stability of **2** in glacial acetic acid in air, at 298 K........ 56
Figure S86. Stability of **2** in DCM in air, at 298 K........ 56
Figure S87. Stability of **2** in 1:1 DBrM:toluene in air, at 298 K........ 57
Figure S88. Solvatochromism of **1** across solvents at 298 K........ 57
Figure S89. Solvatochromism of **2** across solvents at 298 K........ 58

5. Photoluminescence quantum yield measurements........ 58
Figure S90. Photoluminescence quantum yield determination of **1** (red) in DCM at 298 K. **PtdppeTTFtt**[9] **(**blue) used as a reference (PLQY = 0.136% in DCM)........ 58
Figure S91. Photoluminescence quantum yield determination of **2** (red) in DCM at 298 K. **PtdppeTTFtt**[9] **(**blue) used as a reference (PLQY = 0.136% in DCM)........ 59

6. Molar absorption coefficient determination........ 59
Figure S92. Absorption spectra of **1** and **2** in DCM at 298 K........ 59

7. Variable wavelength excitation and oxygen experiments........ 59
Figure S93. Photoluminescence spectra of **1** in anaerobic DCM at 298 K, at varying excitation wavelengths. 60
Figure S94. Photoluminescence spectra of **2** in anaerobic DCM at 298 K, at varying excitation wavelengths. 60
Figure S95. Photoluminescence spectra of **2** in anaerobic DCM and a DCM blank at 298 K, using 1300 nm light for excitation........ 61
Figure S96. Photoluminescence spectra of **2** in anaerobic DCM and after increasing exposure to oxygen at 298 K, using 1300 nm light for excitation........ 61

EPR spectroscopy........ 62

1. CW spectra........ 62
Figure S97. Experimental X-band CW EPR spectrum of a 1×10^{-4} mol/L 1:1 DCM:toluene solution of (**a**) **2** and (**b**) **2-TTFtt** at 295 K........ 62

2. Pulsed EPR........ 62
Figure S98. Experimental X-band EDFS spectrum of a 1×10^{-4} mol/L 1:1 DCM:toluene solution of **1** at 295 K........ 62
Figure S99. Rabi oscillations for a 1×10^{-4} mol/L 1:1 DCM:toluene solution of **1** at 295 K........ 63
Figure S100. Spin–lattice relaxation time (T_1) of a 1×10^{-4} mol/L 1:1 DCM:toluene solution of **1** at 295 K. T_1 = 0.916 µs........ 63

Figure S101. Phase memory time (T_m) of a 1×10^{-4} mol/L 1:1 DCM:toluene solution of **1** at 295 K. T_m = 158 ns. 64
Figure S102. Measurement of (upper) spin−lattice relaxation times (T_1) and (lower) phase memory times (T_m) of 1×10^{-4} mol/L DCM:toluene solution of **2** across various temperatures. Normalized echo intensity shown as a function of delay time T and 2τ from 153 K to 10 K. See methods for experimental and fitting details. 65
Figure S103. Variable-temperature inversion recovery experiments of **2**, showing the temperature dependence of T_1. The 1/T_1 vs T data is fitted by the Raman spin−lattice process, $1T1 = aTm$, where a is a pre-factor and m is the exponent. Fitting revealed m = 3.26 66
Figure S104. Experimental X-band EDFS spectrum of a 1×10^{-4} mol/L 1:1 DCM:toluene solution of **2-TTFtt** at 30 K. 66
Figure S105. Spin−lattice relaxation time (T_1) of a 1×10^{-4} mol/L 1:1 DCM:toluene solution of **2-TTFtt** at 30 K. T_1 = 840.49 μs. 67
Figure S106. Phase memory time (T_m) of a 1×10^{-4} mol/L 1:1 DCM:toluene solution of **2-TTFtt** at 30 K. T_m = 6.112 μs. 67

X-ray crystallography* *68
Table S1. Crystallographic data for complex **1**. 68
Table S2. SXRD coordinates of **1**. 68

Computational methodology* *72

Computational Analysis and Electronic Structure 72
Table S3. Triplet-singlet gaps for **1** and **2**. 73
Table S4. Predicted diradical character for **1** and **2**. 73
Figure S107. HONO-LUNO diagrams for **1** and **2**, showing multireference character. 73

Predicted Photophysical Properties* *74
Table S5. Predicted absorption and emission for singlet and triplet states for **1** and **2**, along with calculated oscillator strengths. 74
Figure S108. Calculated singlet absorption and emission for **1**. 74
Figure S109. Calculated triplet absorption for **1**. 75
Table S6. Calculated vibrational frequencies (in cm^{-1}) and intensities for **1** and its corresponding TTFtt analog.[11] Cells with no values were either not reported or a vibration at that frequency was not predicted. Green cells indicate core vibrations. 76

References* *76

General synthetic methods

All linker synthesis, metalation, and oxidation procedures were performed under inert conditions (dry N_2) in a MBraun UNIlab glovebox. Elemental analyses (C, H, N) were conducted by Midwest Microlabs. All solvents used were dried, purged with N_2 on a Pure Process Technology solvent system, and subsequently filtered through activated alumina and stored over 4 Å molecular sieves. $Pt(P(p\text{-}OCH_3Ph)_3)_2Cl_2$,[1] $Pt(PPh_3)_2Cl_2$,[2] **2-TTFtt**,[3] and $[Fc^{BzO}][BAr^F{}_4]$,[4] and were prepared according to literature procedures. All other chemicals and reagents were purchased from commercial sources and used as received.

Characterization and analysis methods

Single-crystal X-ray Diffraction (SXRD)

SXRD data was collected at 100 K on a Bruker D8 VENTURE diffractometer equipped with a microfocus Mo-target X-ray tube (λ = 0.71073 Å) and a PHOTON 100 CMOS detector. A single crystal was mounted on a cryo-loop and transferred into the diffractometer nitrogen stream before collection. Data reduction and integration were performed with the Bruker APEX3[5] software package (Bruker AXS, version 2015.5-2, 2015). Data was scaled and corrected for absorption effects using the multi-scan procedure as implemented in SADABS (Bruker AXS, version 2014/54, 2015). The crystal structure was solved by SHELXT[6] (version 2014/55) and refined by a full-matrix least-squares procedure using OLEX26[7] (XL refinement program version 2018/17). Disorder was modeled with common restraints. We note a couple A-level alerts, resulting from extraneous electron density near the heavy atoms Pt and Se. However, the bond lengths and geometrical aspects of the core scaffold are still sufficiently high resolution for interpretation. Deposition number 2415439 contains the supplementary crystallographic data for this paper. This data can be obtained free of charge via the joint Cambridge Crystallographic Data Centre (CCDC) and Fachinformationszentrum Karlsruhe Access Structures service.

Cyclic voltammetry

Cyclic voltammetry measurements were performed using a silver wire pseudoreference, glassy carbon working electrode, and a platinum wire counter electrode. Each voltammogram was referenced to an internal standard (Fc^+/Fc). All measurements were acquired using a BASi Epsilon potentiostat and analyzed using the BASi Epsilon software version 1.40.67NT.

FT-IR spectroscopy

All IR measurements were performed by dropcasting DCM solutions onto KBr plates. Each spectrum was acquired on a Bruker Tensor II; both background subtractions and baseline corrections were applied for each complex using the OPUS software.

NMR spectroscopy

^{1}H, ^{31}P, and ^{195}Pt NMR spectra were acquired on Bruker DRX 400 and 500 spectrometers. Residual solvent peaks were referenced for all ^{1}H NMR measurements and 85% phosphoric acid and sodium hexachloroplatinate were used as references for ^{31}P and ^{195}Pt NMR measurements, respectively. Evans method measurements were conducted in CD_2Cl_2 with a capillary insert of 95/5 w/w% CD_2Cl_2/DCM. Pascal's constants were used to correct for the diamagnetic contribution.[8]

UV-vis-NIR

UV-vis-NIR measurements were performed using a Shimadzu UV-3600 Plus dual beam spectrophotometer and a Thermo Scientific Evolution 300 spectrometer and analyzed using VisionPro software.

Photoluminescence spectroscopy

Room temperature emission spectra were acquired on a Horiba Scientific PTI QuantaMaster fluorometer. Low temperature emission spectra were recorded by loading samples in 1:1 DBrM:toluene into 0.5 mm cuvettes, sealing with vacuum grease, and cooling in a Janis cryostat.

Transient absorption spectroscopy

NIR transient absorption spectroscopy was performed using a 5 kHz amplified titanium:sapphire laser with a 120 fs laser pulsewidth. A portion of the 800 nm fundamental output was focused into a sapphire crystal to produce near-infrared continuum probe pulses that were mechanically delayed. Pump pulses were tuned to 900 nm using an optical parametric amplifier, and a mechanical chopper reduced the repetition rate to 2.5 kHz. Samples were excited with 0.2 mW of pump power.

SWIR transient absorption with a shortwave infrared probe was performed using a 35 fs amplified Ti:sapphire laser operating at 2 kHz. The 2 mJ output was beamsplit equally and directed to two optical parametric amplifiers (OPAs). One OPA was tuned to produce a signal wavelength of 1200 nm that was used as the pump beam after mechanically reducing the repetition rate to 1 kHz and mechanically delayed. The probe beam was produced using the idler output of the second OPA that was tuned to 2400 nm and focused into a sapphire crystal to produce a shortwave infrared continuum. This beam was dispersed in a grating spectrometer and detected on a single-shot basis using a red-extended InGaAs array detector.

Lifetime measurements

Time-correlated single photon counting was performed on the samples using a 975 nm diode laser with a 60 ps pulsewidth operating at 20 MHz. Collected PL was dispersed using a 0.3 m spectrograph and detected with a superconducting nanowire single photon detector and multichannel scaler with 25 ps bin width. Of note, the instrument response function (IRF) falls around ~50 ps.

Photoluminescence quantum yield determination

The samples, in 1 cm quartz fluorescence cuvettes (Starna) were normalized to optical densities at 900 nm using UV-Vis-NIR measurements. All samples were kept at or below 0.1 OD at 900 nm to minimize reabsorption effects. Three different ODs were tested per sample, forming a gradient upon plotting against the integrated photoluminescence. The individual quantum yields were then calculated using this gradient and the reference method (reference is PtdppeTTFtt, PLQY = 0.136% in DCM at 298K[9]). The following equation was used:

$$PLQY_s = PLQY_r(\frac{m_s}{m_r})(\frac{n_s}{n_r})^2$$

Where *m* is the gradient of the OD vs integrated PL plot and *n* is the refractive index of the solvent. *r* and *s* refer to the reference and the sample, respectively.

Nonradiative rate determination
Both the experimentally determined quantum yield values, ϕ , and the TA singlet lifetimes were utilized to determine the radiative and nonradiative rates through the expression $\phi = k_r/(k_r+k_{nr})$, where the denominator, (k_r+k_{nr}), is the reciprocal of the singlet lifetime, $\tau_{TA,S}$.

Electron paramagnetic resonance (EPR) measurements
Continuous wave (CW) electron paramagnetic resonance (EPR) spectroscopy.
The CW EPR spectra of **1** and **2** were acquired using a Bruker E500 spectrometer operating at X-band (9.6 GHz) frequencies at the Instrumentation and Service Center for Molecular Sciences, Westlake University. The magnetic field was calibrated for accuracy by a standard BDPA radical sample, which revealed + 0.0351 mT correction. The modulation amplitude was set to 0.1 mT for **2** and **2-TTFtt**, and 0.02 mT for **1**. The microwave power was 0.1 mW. The solution was prepared under an N_2 atmosphere by dissolving crystalline material in 1:1 DCM: toluene and diluting to 1×10^{-4} mol/L. The sample was flame sealed in a quartz tube and the CW EPR spectrum was recorded at room temperature (295 K). The CW EPR spectrum was fitted using the chili function in EasySpin in MATLAB R2023b with a S = 1 spin. Fitting for **1** revealed $g_\perp$ = 2.00705, $g_{//}$ =2.00569, D = 15.961 MHz, and a tumbling correlation time of 16.12 ns, and 0.05578 mT of Gaussian broadening. Fitting for **2-TTFtt** revealed isotropic g = 2.00278, A_{Pt} = 10.1 MHz, A_P = 5.2 MHz, D = 0, and 0.18 mT of Gaussian broadening.

Room-temperature pulse EPR spectroscopy.
The room-temperature echo-detected field swept (EDFS), inversion recovery, and Hahn echo decay experiments of **1** were acquired with a CIQTEK EPR100 spectrometer operating at X-band (9.6 GHz) frequencies equipped with an X-band pulsed ENDOR dielectric resonator EN4202DR at the Instrumentation and Service Center for Molecular Sciences, Westlake University. Samples were prepared under inert conditions by dissolving crystalline **1** in 1:1 DCM:toluene and diluting to 1×10^{-4} mol/L. The sample was flame sealed in a quartz tube.

For **1**, π/2 and π pulses were applied with lengths of 16 ns and 32 ns, respectively. The pulse lengths were optimized with a three-pulse nutation sequence (nutation pulse – T – π/2 – τ – π – τ – echo) where the length of the nutation pulse was varied, and delays were set as τ = 120 ns and T = 400 ns. The relationship between the intensity of the echo and the length of the nutation pulse exhibits a nutation pattern. The microwave attenuation was tuned such that the corresponding pulse lengths of the local maxima and minima are integer multiples of 32 ns. Pulses were phased by applying a two-pulse Hahn echo sequence (π/2 – τ – π – τ – echo) at the resonant magnetic field and adjusting the phase to maximize the sum of square of the real component and minimize the sum of square of the imaginary component of the Hahn echo. All pulse EPR data were further phased by maximizing the sum of square of their real component and minimizing the sum of square of their imaginary component. All experiments were conducted with the shot repetition time (SRT) being longer than five times of T_1. When echo integration was applied, approximately the top 2/3 of the echo was integrated to reduce the influence of noise.

The echo-detected field sweep (EDFS) spectrum was collected with a two-pulse Hahn echo sequence (π/2 – τ – π – τ – echo) with 4.0 mT scan width, 120 ns delay time, 100 shots per point, 512 data points, and at 295 K for **1**. For **2-TTFtt**, scan width was 20 mT, delay time was 200 ns,

shots per point was 100, number of points was 1024, and temperature was 30 K. π/2 and π pulses were applied with lengths of 16 ns and 32 ns, respectively. Two-step phase cycling was employed with pulse phases of (+x, +x) and (-x, +x) to cancel background drift and the defense pulse. Integration of the echo was plotted against the magnetic field strength, giving an EDFS spectrum.

The spin–lattice relaxation time (T_1) was characterized by an inversion recovery sequence (π – T – π/2 – τ – π – τ – echo) with 512 data points, at the magnetic field with the maximum EDFS intensity. τ was 120 ns, T was 400 ns, increment of T was 40 ns, and shots per point was 100. Four-step phase cycling was employed with pulse phases of (+x, -x, +x) (+x, +x, +x) (-x, -x, +x) and (-x, +x, +x) to cancel background drift, unwanted echoes, and the defense pulse. Integration of the echo was plotted against the delay time, T, giving the inversion recovery curve. It was fitted by a monoexponential decay function:

$$I = ae^{-\frac{T}{T_1}} + I_0$$

where I is echo intensity, a is a pre-factor, and I_0 accounts for baseline drift.

The phase memory time (T_m) was characterized by a two-pulse Hahn echo sequence (π/2 – τ – π – τ – echo) with 512 data points, at the magnetic field with the maximum EDFS intensity. τ was 120 ns and shots per point was 100. Two-step phase cycling was employed with pulse phases of (+x, +x) and (-x, +x) to cancel background drift and the defense pulse. Integration of the echo was plotted against twice of the delay time, 2τ, giving an echo decay curve. The Hahn echo decay curves collected were fitted by a monoexponential decay function:

$$I = ae^{-\frac{2\tau}{T_m}} + I_0$$

The nutation experiment was conducted with a three-pulse sequence (nutation pulse – T – π/2 – τ – π – τ – echo) with 100 shots per point, 512 data points, at the magnetic field with the maximum EDFS intensity. The length of the nutation pulse started at 6 ns and was incremented with 2 ns per step. τ was fixed at 120 ns. Four-step phase cycling was employed with pulse phases of (+x, -x, +x) (+x, +x, +x) (-x, -x, +x) and (-x, +x, +x) to cancel background drift, unwanted echoes, and the defense pulse. Integration of the echo was plotted against the length of the nutation pulse, giving a nutation curve.

Low temperature pulse EPR spectroscopy.

The variable-temperature inversion recovery and Hahn echo decay experiments of **2** and **2-TTFtt** were acquired with a CIQTEK EPR100 spectrometer operating at X-band (9.6 GHz) frequencies at the Instrumentation and Service Center for Molecular Sciences, Westlake University. Samples were prepared under inert conditions by dissolving crystalline **2** or **2-TTFtt** in 1:1 DCM:toluene mixture (volume ratio) and diluting to 1×10^{-4} mmol/L. This mixture is a glassy solvent, so that molecular aggregation can be avoided when the solution is frozen. Samples were flame sealed in a quartz tube.

The cryogenic temperature was controlled by a closed-loop helium cryostat EPR-VTS-L-D1-PWC and a Lakeshore 336 temperature controller. Samples were cooled at 10 K for at least 2 hours

before spin dynamics experiments. The thermal equilibrium was monitored by the T_1 of the sample: it was established when two consecutive inversion recovery experiments, separated by 5 min, revealed T_1 values with the difference less than 1%. Such thermal stabilization process typically took 20 - 30 min, after which T_1 and T_m were acquired. We collected T_1 and T_m from 10 to 133 K of **2** in one experimental session.
Inversion recovery and Hahn echo experiments were conducted with the same methods discussed in the "Room-temperature pulse EPR spectroscopy" section, except with τ = 200 ns. The shot repetition time (SRT) was adjusted to be longer than five times of T_1 at each temperature. The inversion recovery curves were all fitted with a monoexponential decay function:

$$I = ae^{-\frac{T}{T_1}} + I_0$$

The Hahn echo decay curves were fitted by a stretched exponential decay function:

$$I = ae^{-(\frac{2\tau}{T_m})^q} + I_0$$

where I is echo intensity, a is a pre-factor, q is the stretch factor, and I_0 accounts for baseline drift.

Transient EPR measurements

The X-band time resolved EPR measurements were performed on a Bruker Elexsys E580 X EPR spectrometer equipped with a split ring resonator (Bruker ER4118X-MS5). The temperature was kept at 85 K using an Oxford Instruments CF935 continuous-flow cryostat cooled with liquid nitrogen and controlled with an Oxford Instruments MercuryiTC. The sample was photoexcited with 7 ns, 2 mJ/pulse, 900 nm pulses generated by an optical parametric oscillator (GWU Basi-scan), pumped with the output of a frequency-tripled Nd:YAG laser (Spectra-Physics Quanta-Ray Lab 150). The laser light was coupled into the resonator via a fiber optic (Thorlabs FT1000UMT) and collimator placed outside the cryostat. The transient magnetization was acquired in quadrature under CW irradiation (~5 mW). The EPR spectra were processed in MATLAB using home written scripts and the simulation package EasySpin v6.0.6.[10]

Synthetic procedures

Synthesis of $[\{(PPh_3)_2Pt\}_2TTFts][BAr^F_4]_2$ (**1**)

Lithium diisopropylamide (2M in tetrahydrofuran/n-heptane/ethylbenzene, 0.245 mL, 0.489 mmol) was added to 8 mL of tetrahydrofuran (THF) and cooled to −78 ˚C. Tetrathiafulvalene (0.025 g, 0.122 mmol) was added to the cooled solution and stirred for 1.5 hours. A lemon yellow-orange suspension formed after this time and selenium powder (0.039 g, 0.489 mmol) was added after and the reaction was warmed to 0 °C. The reaction was stirred at 0 °C for 2 hours, and a shiny orange precipitate formed. Next, $Pt(PPh_3)_2Cl_2$ (0.193 g, 0.245 mmol) was added in 5 mL of THF to the reaction mixture at 0 °C. The reaction was gradually warmed to room temperature and left to stir for 3 hours, after which a shiny dark pink precipitate had formed. The reaction was dried to remove THF and resuspended in 8 mL DCM. The neutral compound was then oxidized with 2.2 equivalents $[Fc^{BzO}][BAr^F_4]$ (0.311 g, 0.270 mmol) in 4 mL DCM. The dark pink suspension turned into a brown and then brown-green solution upon full addition of $[Fc^{BzO}][BAr^F_4]$ and was left to stir 10 minutes. The solution was condensed to 4 mL under vacuum, filtered through celite, and petroleum ether (8 mL) was added. Brown-green crystals immediately began to form. The solids

were washed with petroleum ether several times (3x5 mL) and dried under vacuum. The crude product was redissolved in 6 mL of DCM, filtered through celite, and layered with petroleum ether. The layered solution was cooled to −35 ˚C and left to crystallize overnight. The resulting brown-green crystals were collected and dried (0.310 g, 69% yield). Crystals suitable for SXRD were selected from a DCM/petroleum ether slow diffusion gradient left to crystallize over a 2-day period at −35 °C. ^{1}H NMR (400 MHz, CD_2Cl_2, 298 K): δ 7.25-7.28 (m, PPh_3), 7.39-7.43 (m, PPh_3), 7.55 (s, $[BAr^F_4]^-$), 7.73 (s, $[BAr^F_4]^-$). $^{31}P\{^1H\}$ NMR (162 MHz, CD_2Cl_2, 298 K): δ 12.32 ($J_{Pt,P}$ = 3070 Hz, $^2J_{Se,P}$ = 53 Hz). $^{195}Pt\{^1H\}$ NMR (107 MHz, CD_2Cl_2, 298 K): δ −4635.64. Anal. calc. for **1**, $C_{142}H_{84}B_2F_{48}Pt_2P_4S_4Se_4$: C 46.32%, H 2.30%, N 0%; found: C 46.37%, H 2.51%, N none.

Synthesis of $[\{(P(p\text{-}OCH_3Ph)_3)_2Pt\}_2TTFts][BAr^F_4]_2$ (**2**)

Lithium diisopropylamide (2M in tetrahydrofuran/n-heptane/ethylbenzene, 0.160 mL, 0.321 mmol) was added to 4 mL of tetrahydrofuran (THF) and cooled to −78 °C. Tetrathiafulvalene (0.016 g, 0.080 mmol) was added to the cooled solution and stirred for 1.5 hours. A lemon yellow-orange suspension formed after this time and selenium powder (0.025 g, 0.321 mmol) was added after and the reaction was warmed to 0 °C. The reaction was stirred at 0 °C for 2 hours, and a shiny orange precipitate formed. Next, $Pt(P(p\text{-}OCH_3Ph)_3)_2Cl_2$ (0.156 g, 0.161 mmol) was added in 3 mL of THF to the reaction mixture at 0 °C. The reaction was gradually warmed to room temperature and left to stir for 3 hours, after which a dark pink-red solution had formed. The reaction was dried to remove THF and redissolved in 6 mL DCM. The neutral compound was then oxidized with 2.2 equivalents $[Fc^{BzO}][BAr^F_4]$ (0.204 g, 0.177 mmol) in 4 mL DCM. The dark red solution turned brown upon full addition of $[Fc^{BzO}][BAr^F_4]$ and was left to stir 10 minutes. The solution was condensed to 4 mL under vacuum, filtered through celite, and petroleum ether (8 mL) was added. The solids that crashed out were washed with petroleum ether several times (3x5 mL) and dried under vacuum. The crude product was redissolved in 4 mL of DCM, filtered through celite, and layered with petroleum ether. The layered solution was cooled to −35 °C and left to crystallize overnight. The resulting brown solids were collected and dried (0.183 g, 57% yield). ^{1}H NMR (400 MHz, CD_2Cl_2, 298 K): δ 3.80 (s, OCH_3), 6.75-6.78 (d, Ph), 7.30-7.35 (t, Ph), 7.55 (s, $[BAr^F_4]^-$), 7.72 (s, $[BAr^F_4]^-$). $^{31}P\{^1H\}$ NMR (162 MHz, CD_2Cl_2, 298 K): δ 8.65 ($J_{Pt,P}$ = 3101 Hz, $^2J_{Se,P}$ = 110 Hz). $^{195}Pt\{^1H\}$ NMR (107 MHz, CD_2Cl_2, 298 K): δ −4579.64. Anal. calc. for **1**, $C_{154}H_{108}B_2O_{12}F_{48}Pt_2P_4S_4Se_4$: C 45.75%, H 2.69%, N 0%; found: C 45.77%, H 2.67%, N none.

NMR spectra

1. **^{1}H NMR spectra**

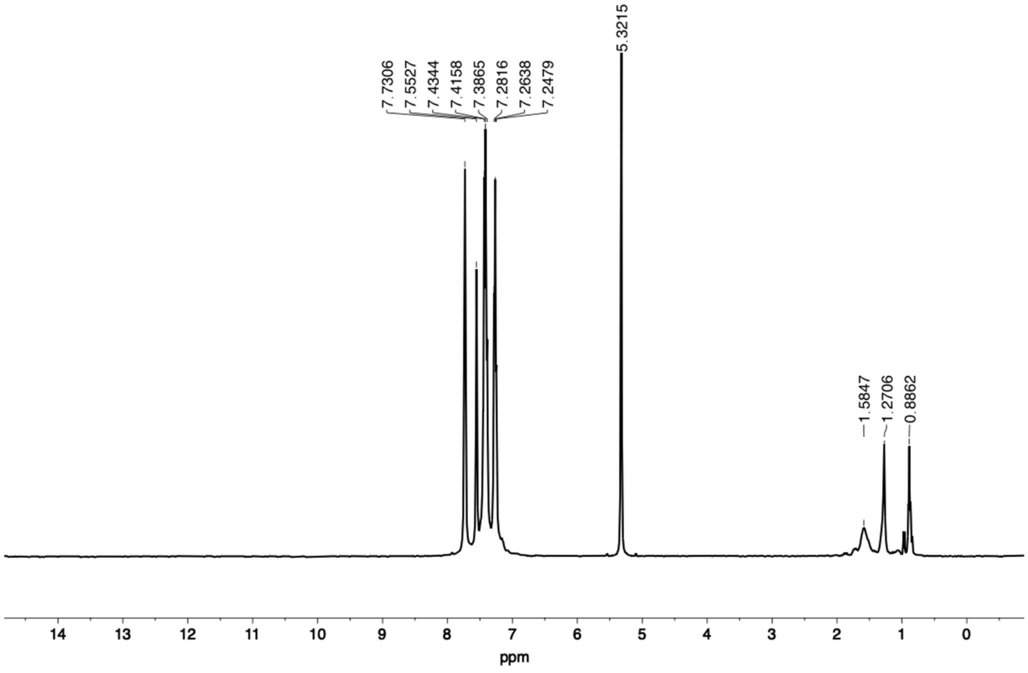

Figure S1. ^{1}H NMR spectrum of **1** in CD_2Cl_2.

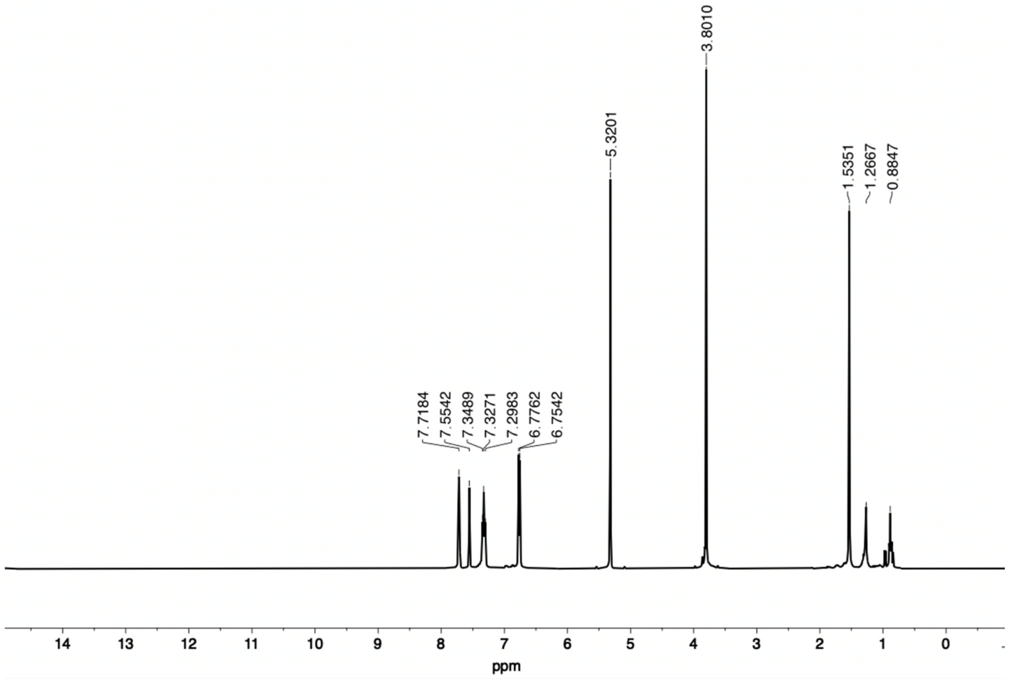

Figure S2. ^{1}H NMR spectrum of **2** in CD_2Cl_2.

2. ^{31}P NMR spectra

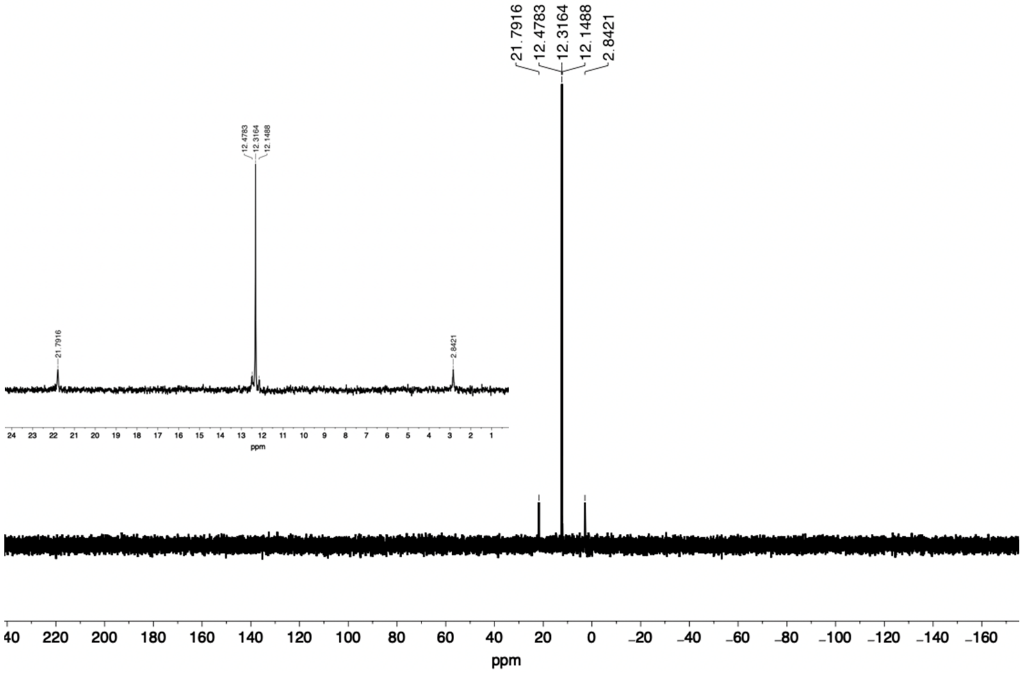

Figure S3. $^{31}P\{^1H\}$ NMR spectrum of **1** in CD_2Cl_2.

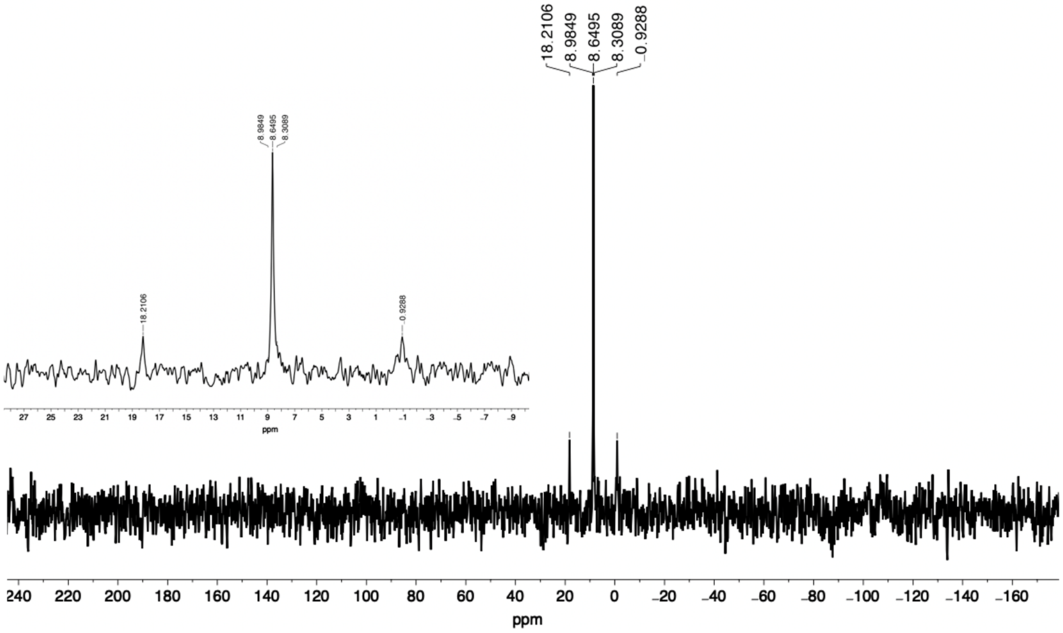

Figure S4. $^{31}P\{^1H\}$ NMR spectrum of **2** in CD_2Cl_2.

3. ^{195}Pt NMR spectra

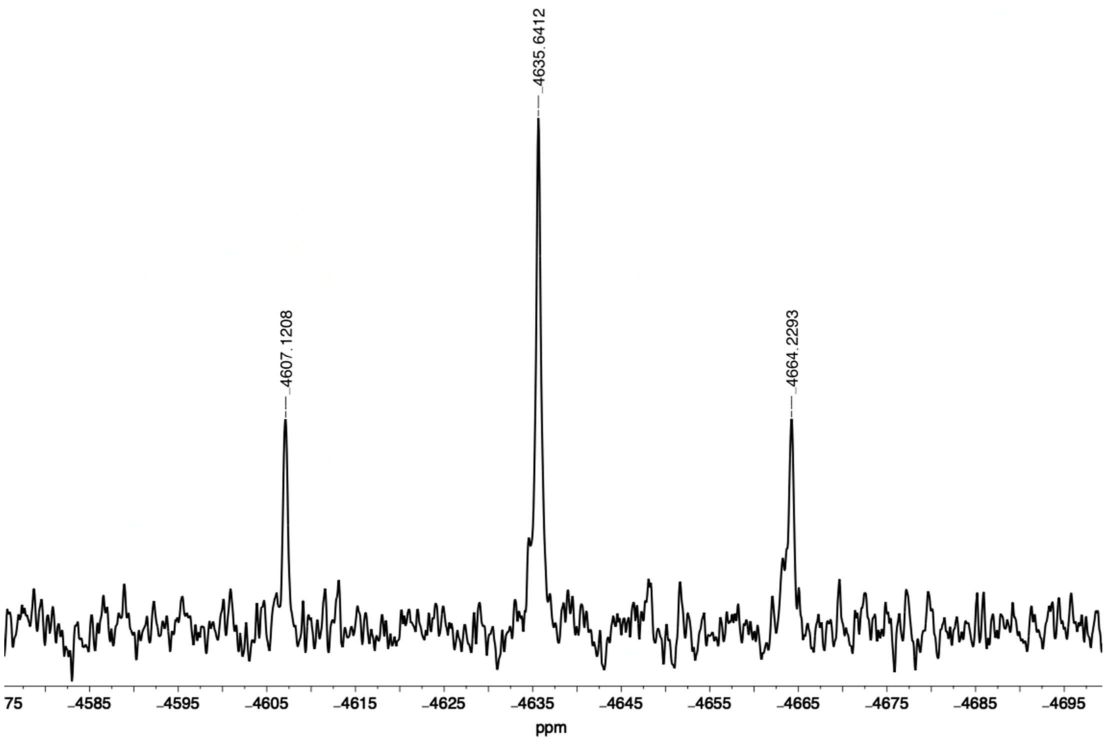

Figure S5. ^{195}Pt{^{1}H} NMR spectrum of **1** in CD_2Cl_2.

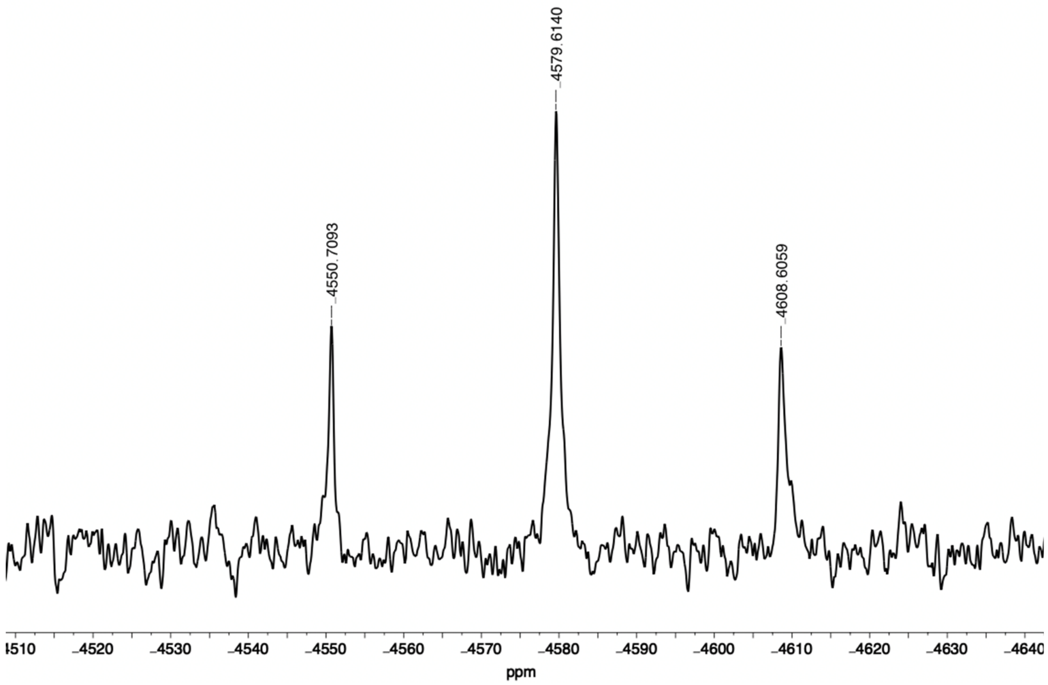

Figure S6. ^{195}Pt{^{1}H} NMR spectrum of **2** in CD_2Cl_2.

4. Evans method measurements

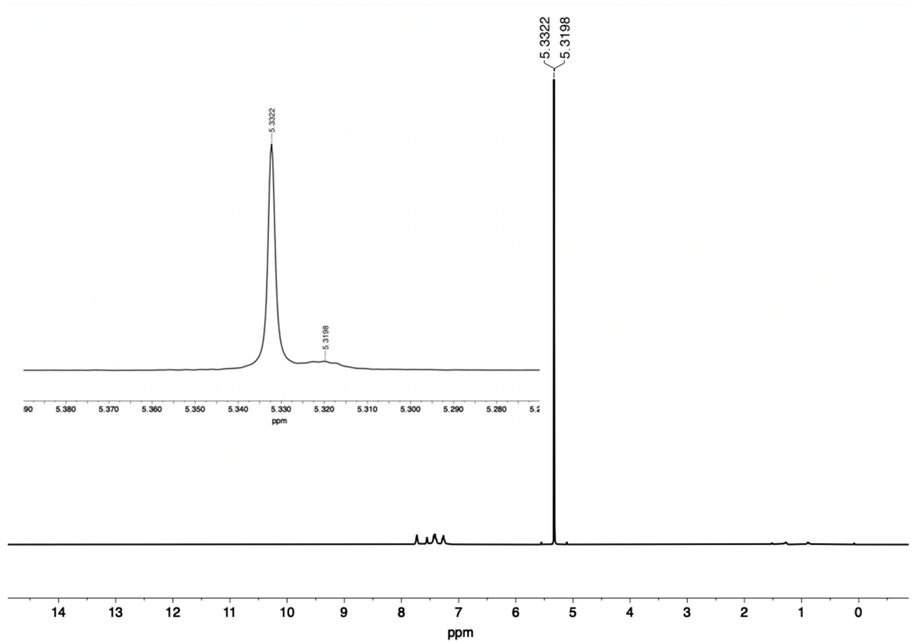

Figure S7. Evans method ^{1}H NMR spectrum of a 6.3 mM solution of **1** in CD_2Cl_2. Magnetic moment: 1.251 μ_B. Inset: Zoomed-in spectrum to show solvent shift.

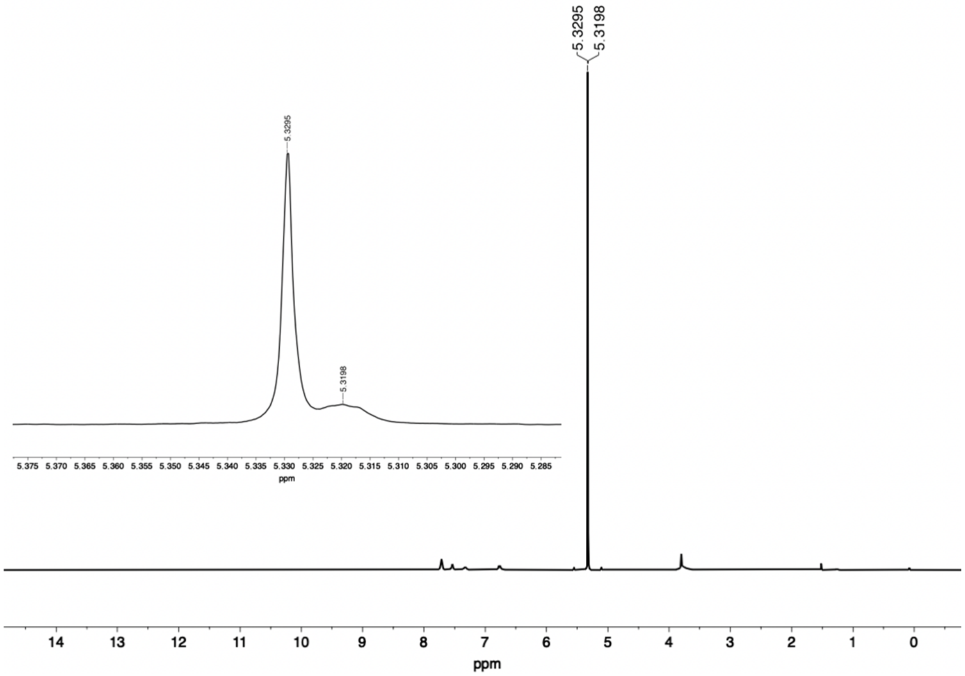

Figure S8. Evans method ^{1}H NMR spectrum of a 5.2 mM solution of **2** in CD_2Cl_2. Magnetic moment: 1.260 μ_B. Inset: Zoomed-in spectrum to show solvent shift.

Infrared spectra

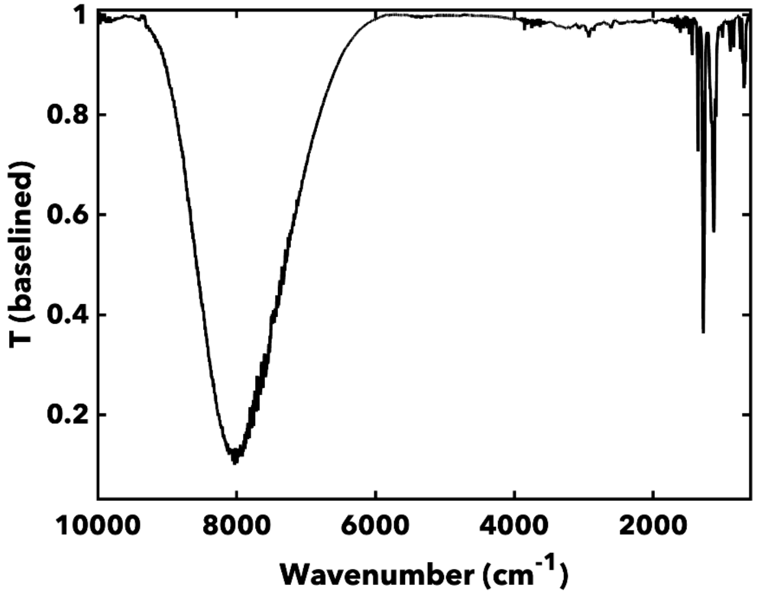

Figure S9. Infrared transmittance spectrum of **1** showing NIR window. Sample prepared by dropcasting a DCM solution.

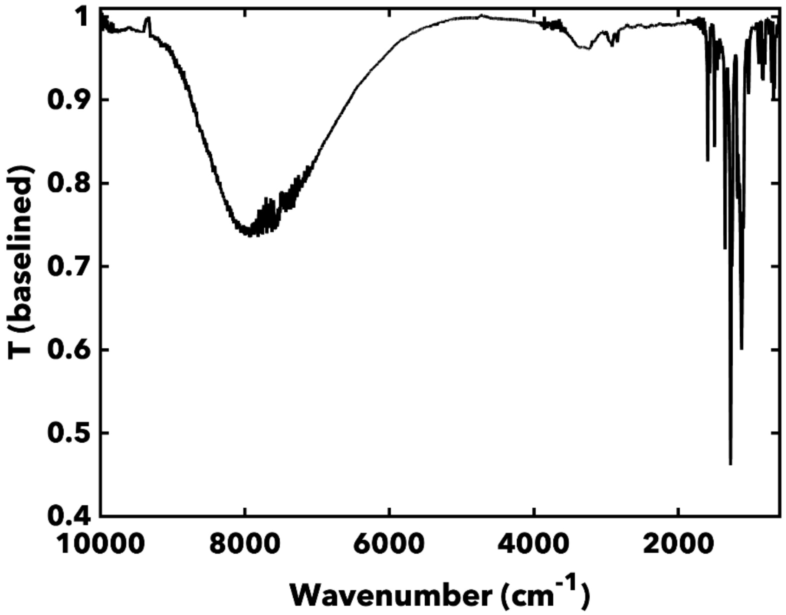

Figure S10. Infrared transmittance spectrum of **2** with NIR window. Sample prepared by dropcasting a DCM solution.

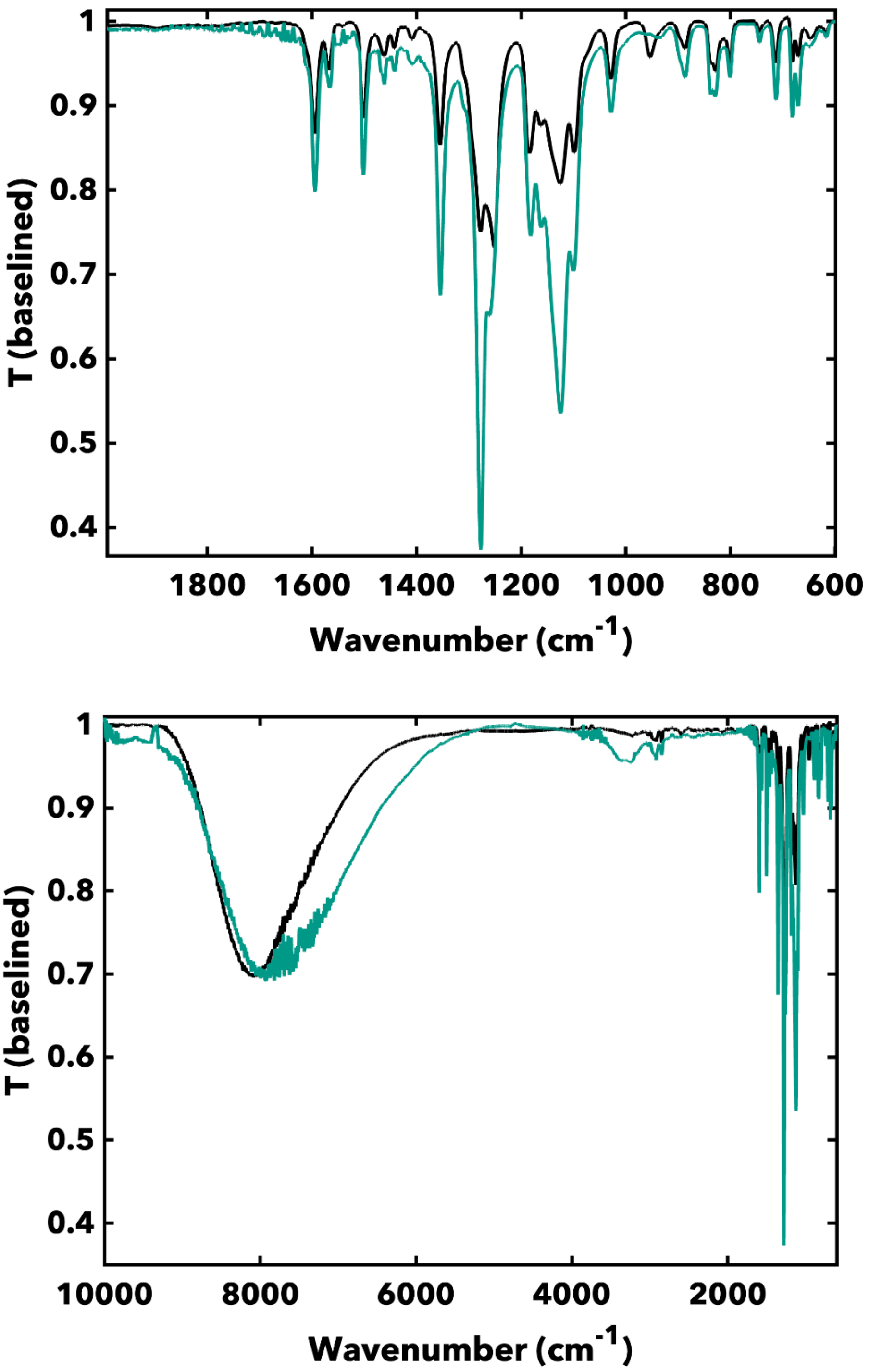

Figure S11. Infrared transmittance spectrum of **2** (teal) compared with **2-TTFtt** (black) with normalized NIR window (lower) and skeletal vibrational shifts (upper). Samples prepared by dropcasting a DCM solution.

Electrochemical measurements

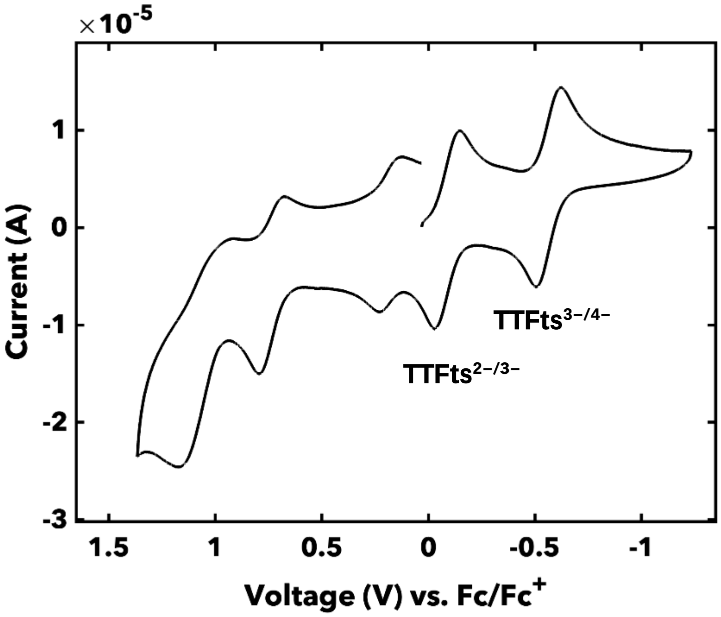

Figure S12. Cyclic voltammogram of 1 mM **1** in 0.1 M [TBA][PF_6] in DCM. Scan rate: 0.1 V/s.

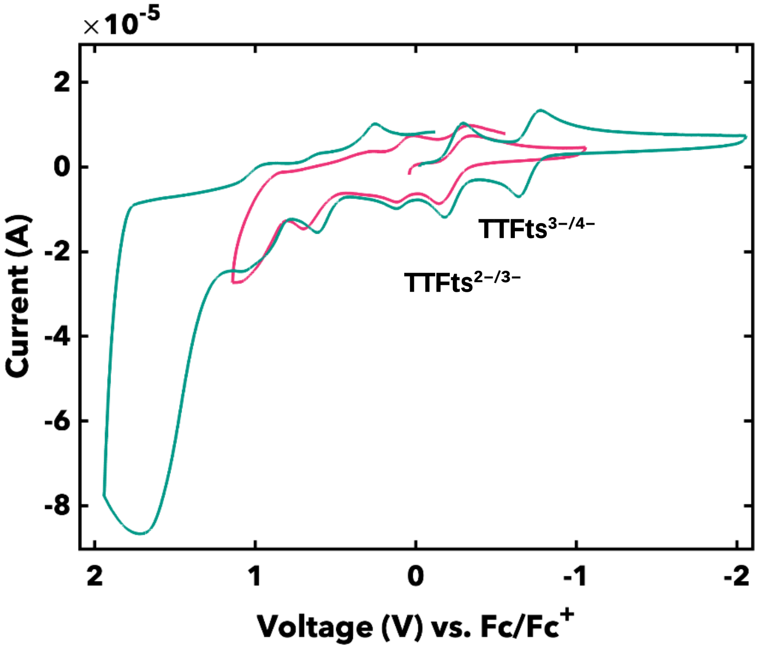

Figure S13. Cyclic voltammogram of 1mM **2** in 0.1 M [TBA][PF_6] in DCM. Scan rate: 0.1 V/s. Magenta trace shows smaller scan window, teal trace shows larger scan window.

Optical spectroscopy

1. Transient absorption spectra and lifetime determination

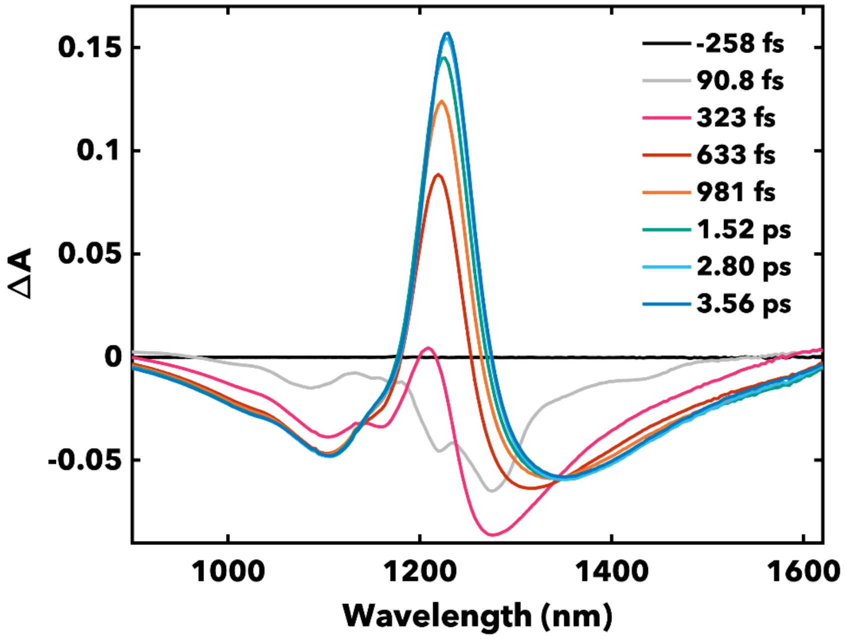

Figure S14. Growth of NIR transient absorption spectra of **1** in DCM at 298 K.

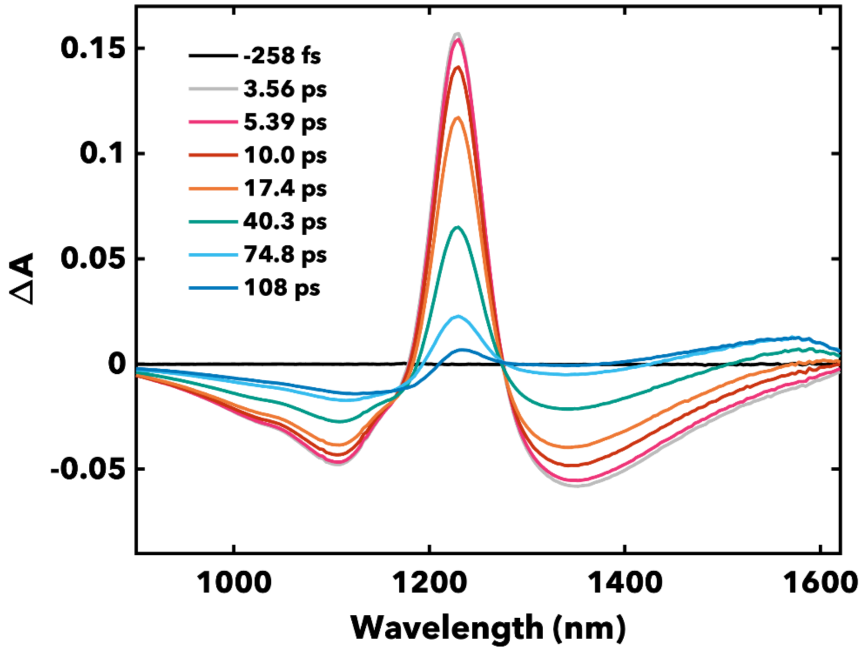

Figure S15. Decay of NIR transient absorption spectra of **1** in DCM at 298 K.

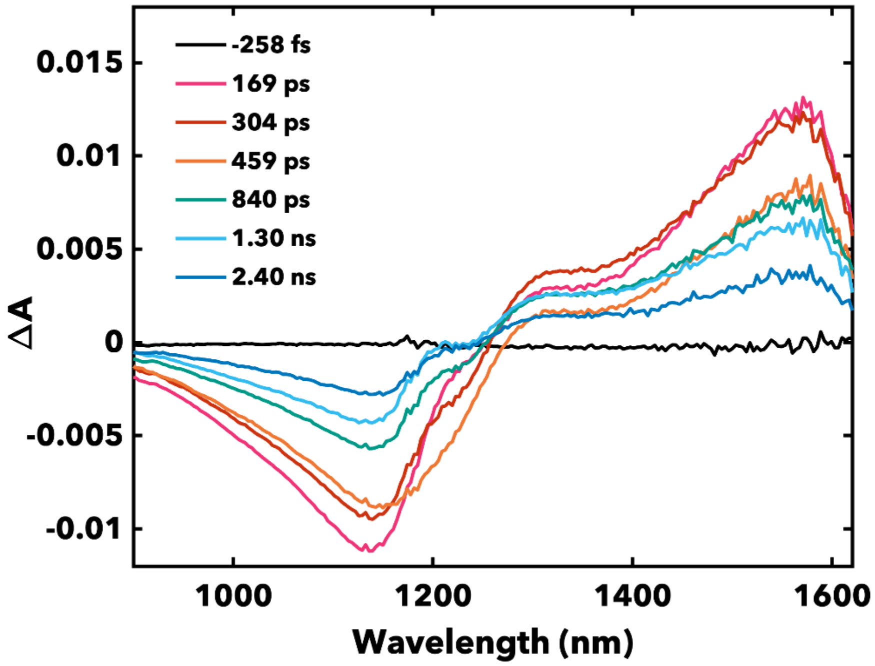

Figure S16. Decay of NIR transient absorption spectra of **1** in DCM at 298 K, at long timepoints.

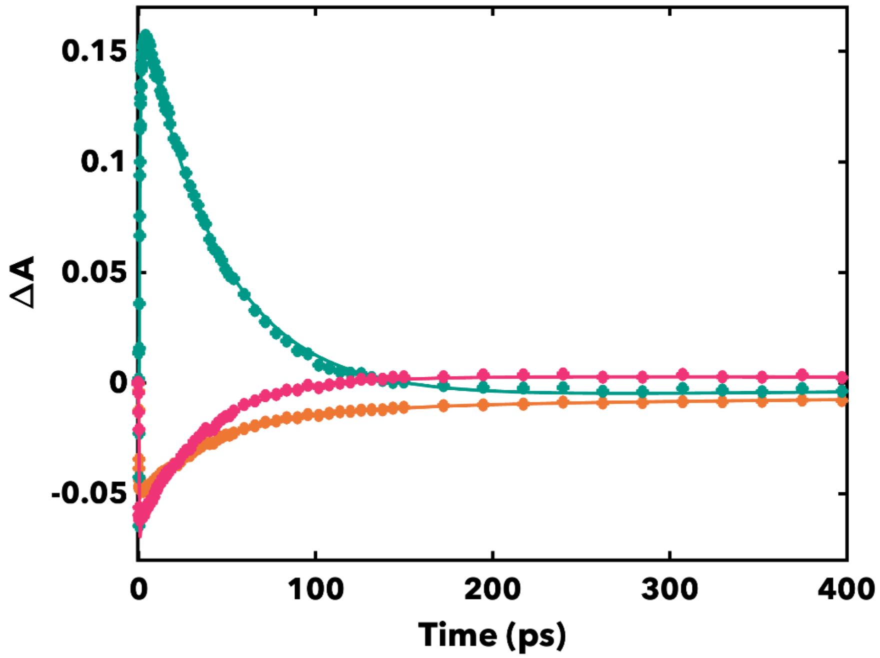

Figure S17. NIR transient kinetics of **1** in DCM at 298 K.

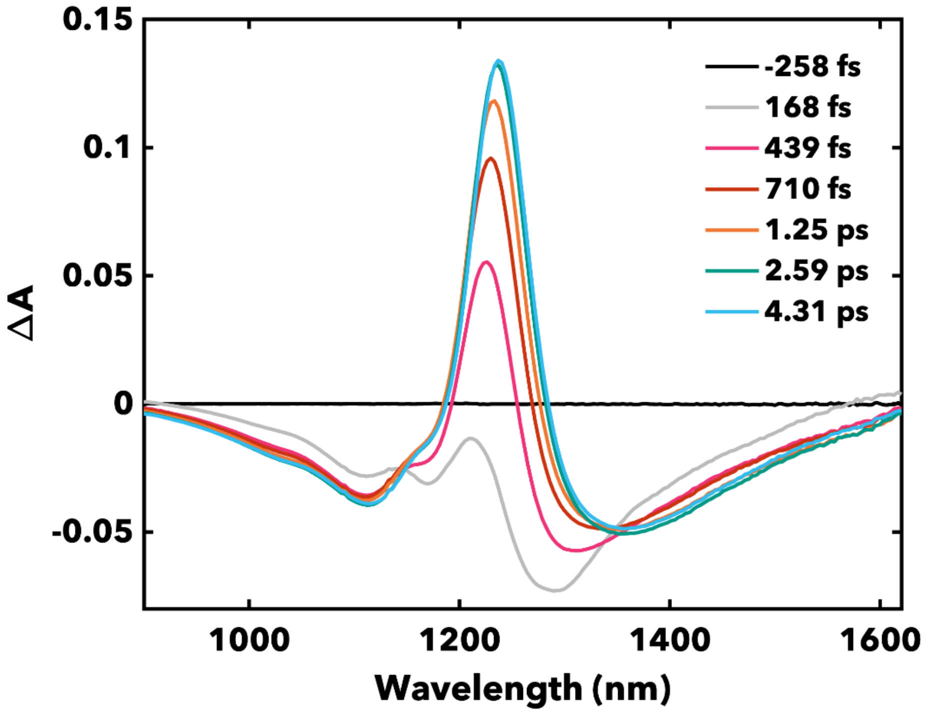

Figure S18. Growth of NIR transient absorption spectra of **1** in CH_2Br_2 (DBrM) at 298 K.

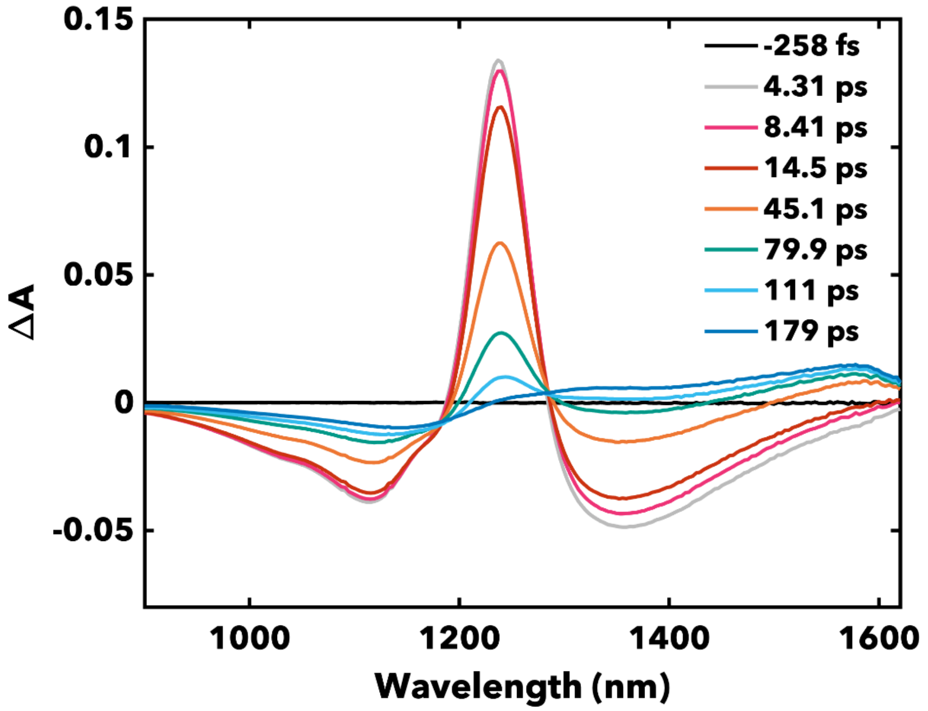

Figure S19. Decay of NIR transient absorption spectra of **1** in DBrM at 298 K.

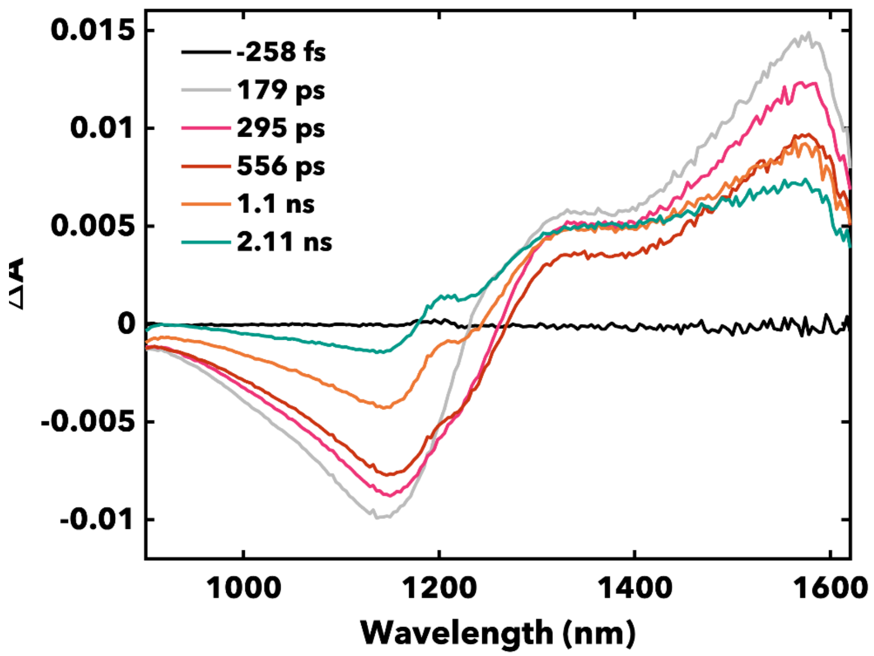

Figure S20. Decay of NIR transient absorption spectra of **1** in DBrM at 298 K, at long timepoints.

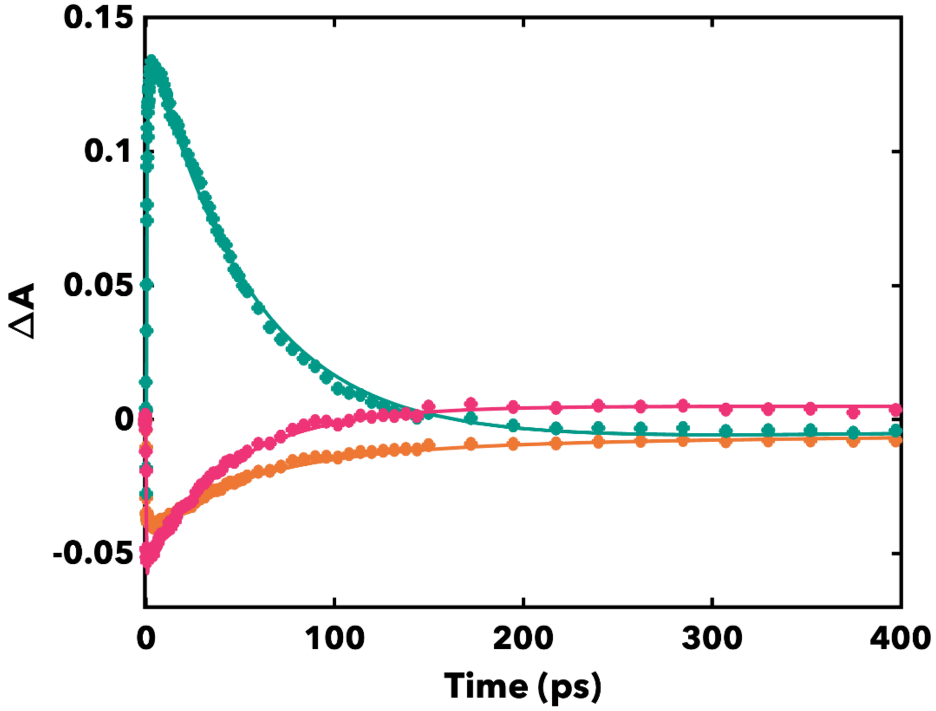

Figure S21. NIR transient kinetics of **1** in DBrM at 298 K.

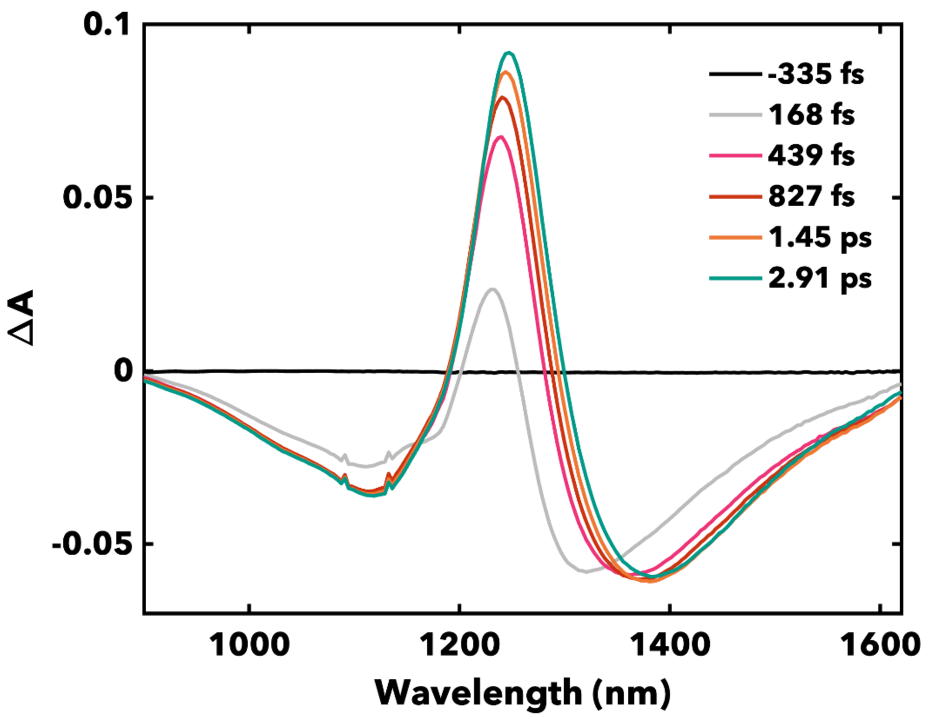

Figure S22. Growth of NIR transient absorption spectra of **2** in DCM at 298 K.

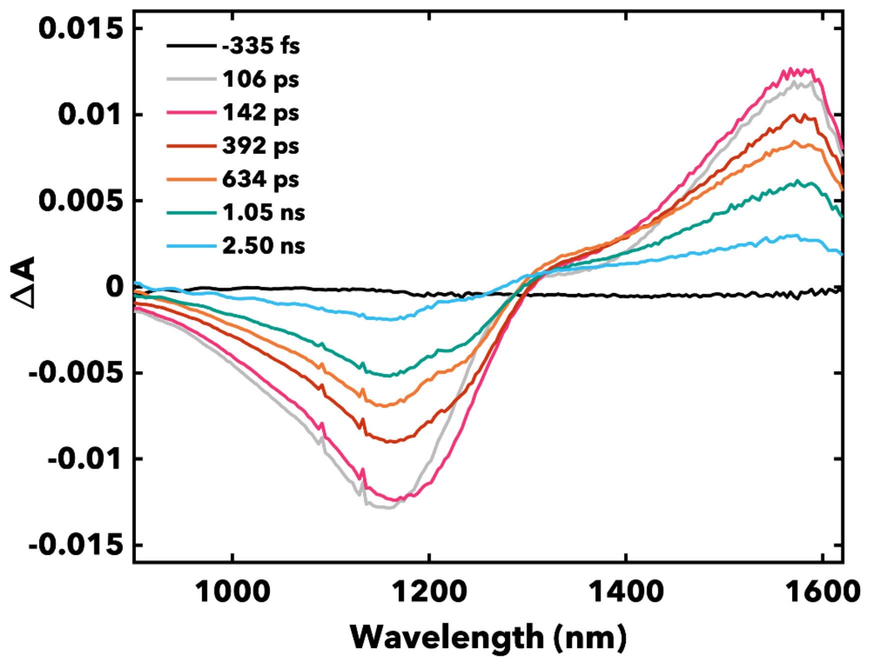

Figure S23. Decay of NIR transient absorption spectra of **2** in DCM at 298 K, at long timepoints.

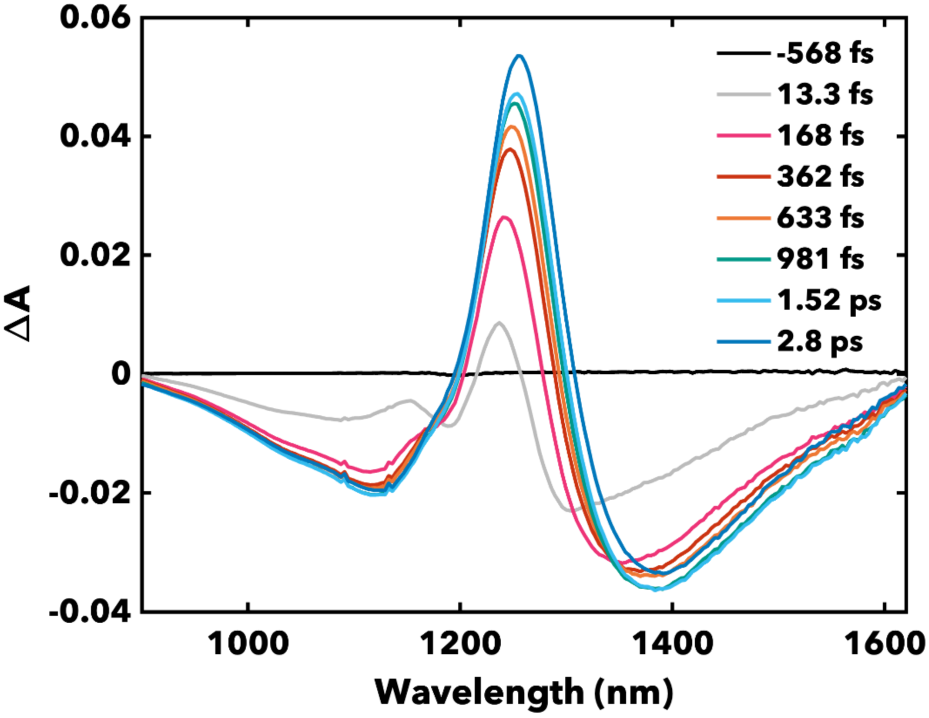

Figure S24. Growth of NIR transient absorption spectra of **2** in DBrM at 298 K.

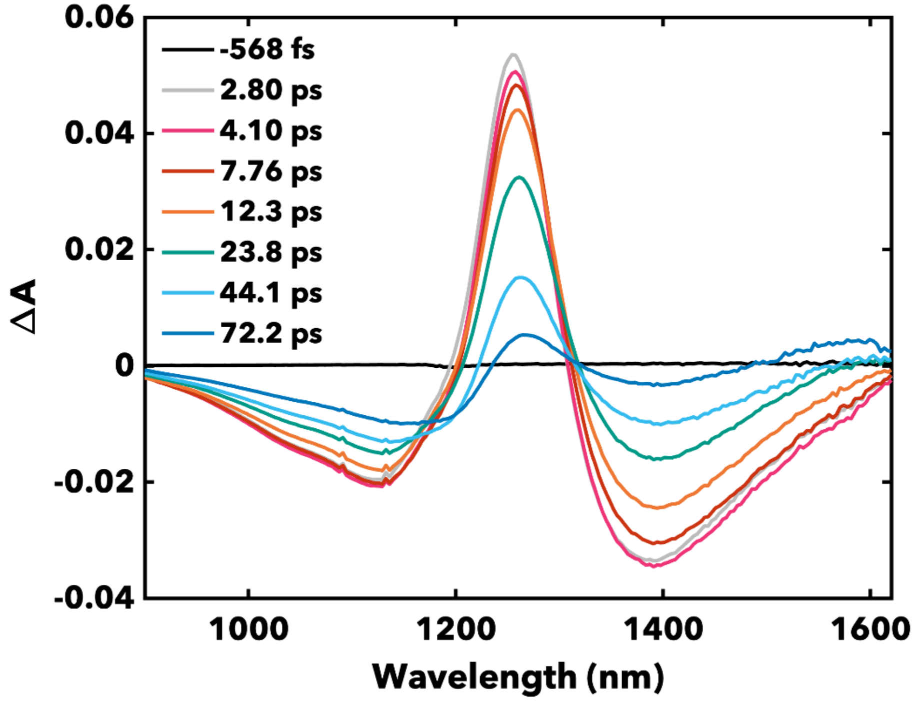

Figure S25. Decay of NIR transient absorption spectra of **2** in DBrM at 298 K.

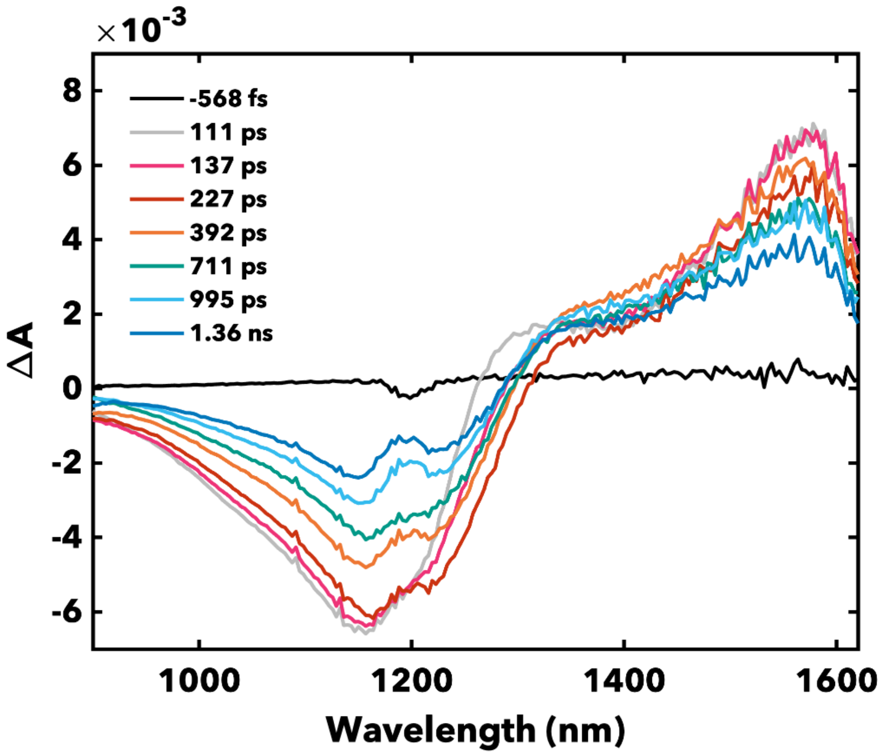

Figure S26. Decay of NIR transient absorption spectra of **2** in DBrM at 298 K, at long timepoints.

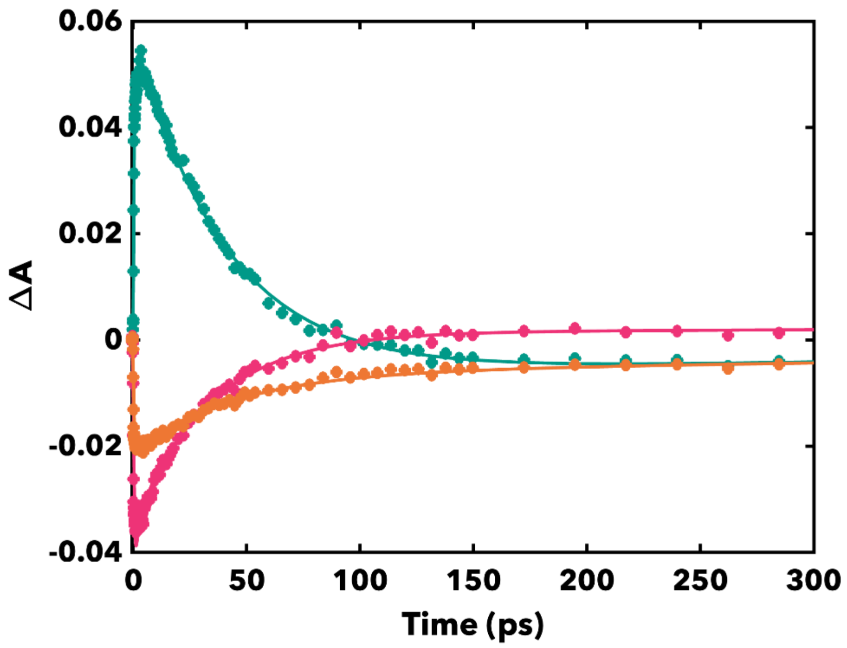

Figure S27. NIR transient kinetics of **2** in DBrM at 298 K.

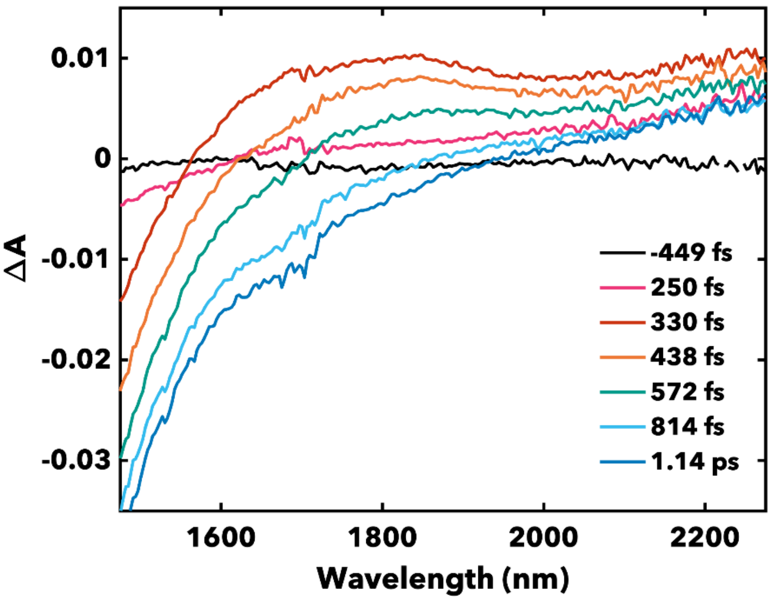

Figure S28. Growth of SWIR transient absorption spectra of **1** in DCM at 298 K.

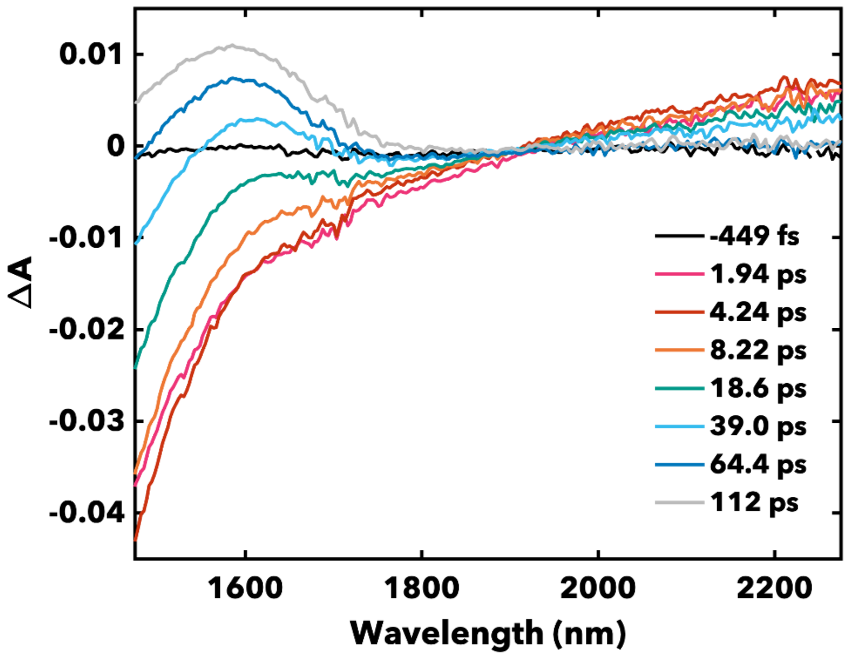

Figure S29. Decay of SWIR transient absorption spectra of **1** in DCM at 298 K.

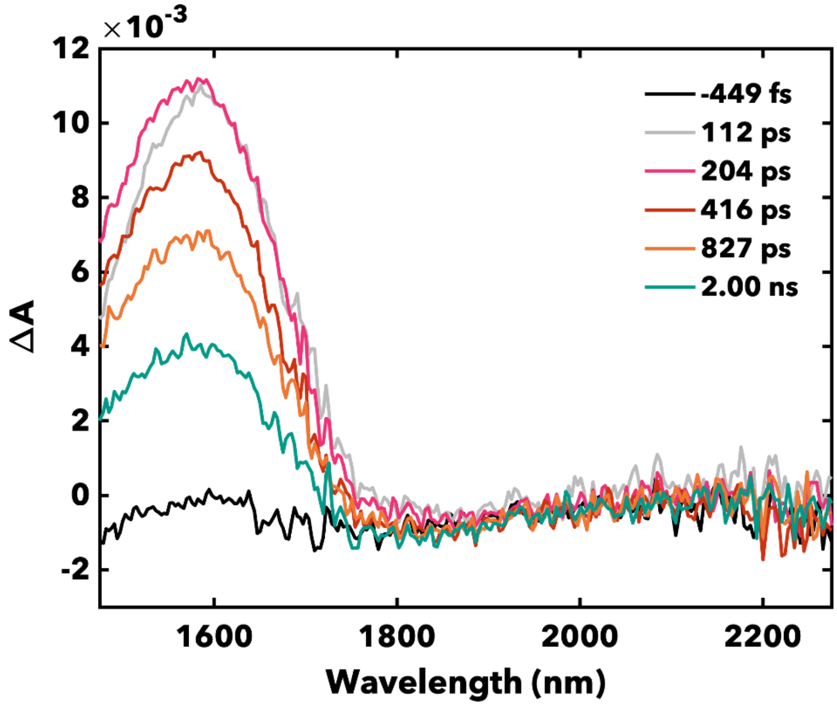

Figure S30. Decay of SWIR transient absorption spectra of **1** in DCM at 298 K, at long timepoints.

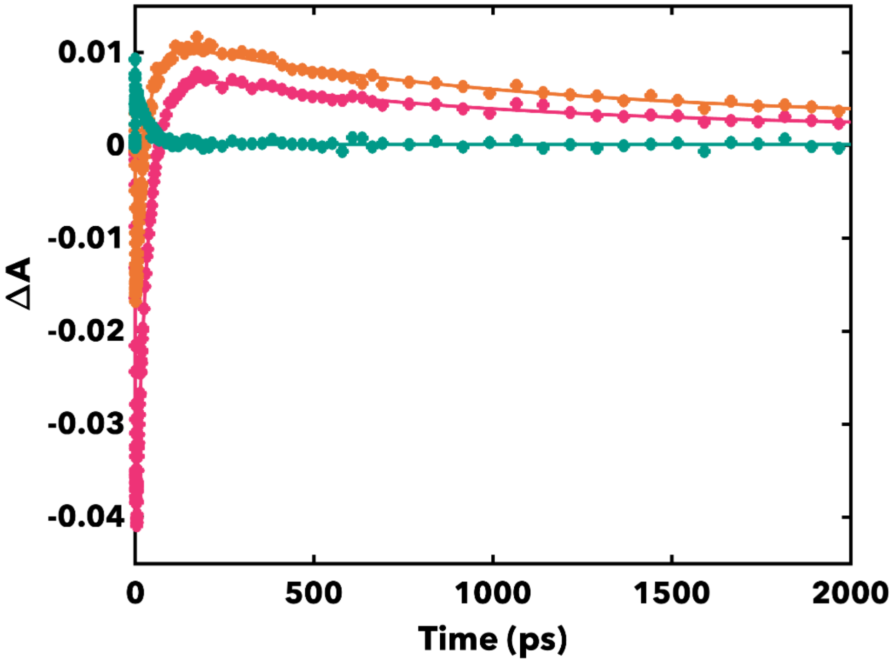

Figure S31. SWIR transient kinetics of **1** in DCM at 298 K.

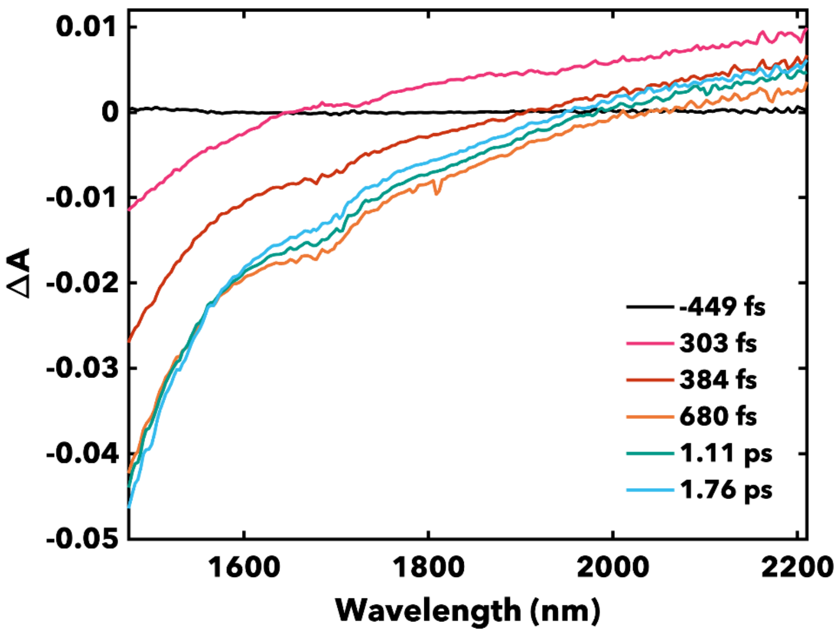

Figure S32. Growth of SWIR transient absorption spectra of **2** in DCM at 298 K.

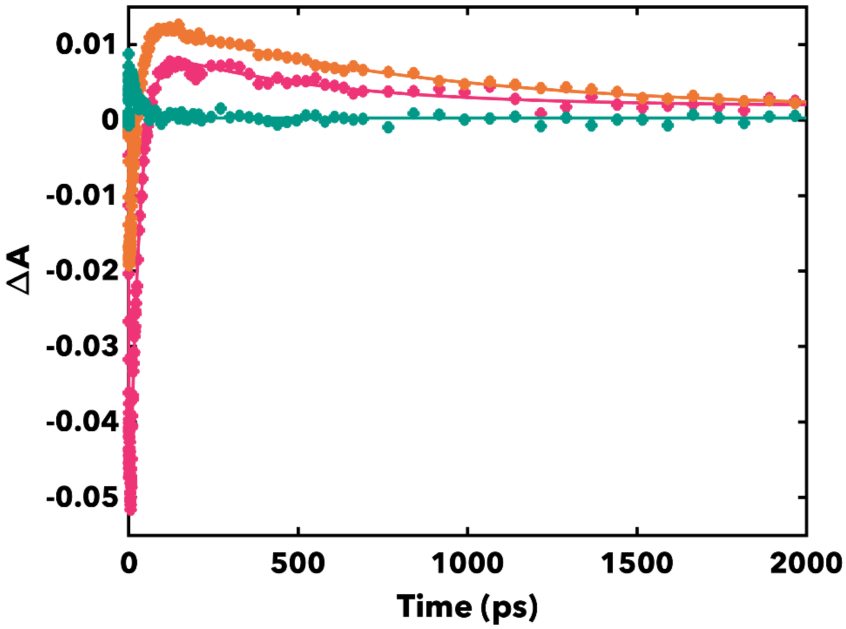

Figure S33. SWIR transient kinetics of **2** in DCM at 298 K.

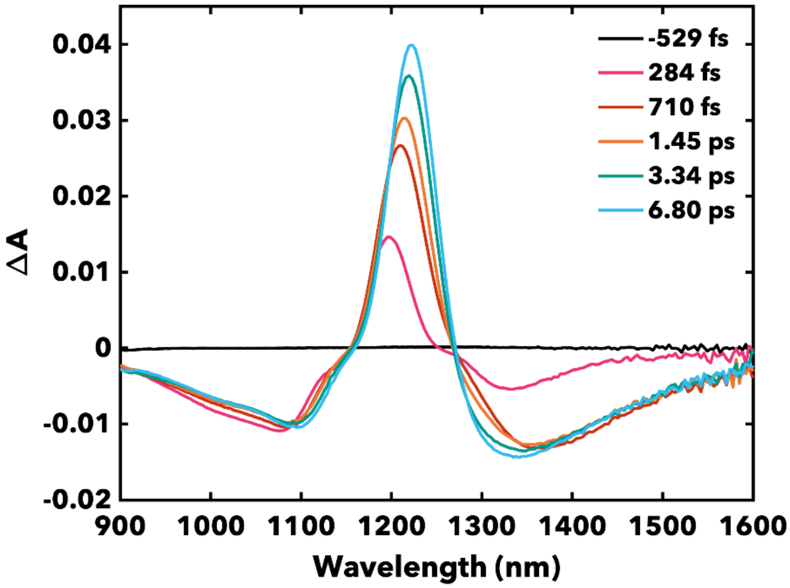

Figure S34. Growth of NIR transient absorption spectra of **1** in 1:1 DBrM:toluene at 298 K.

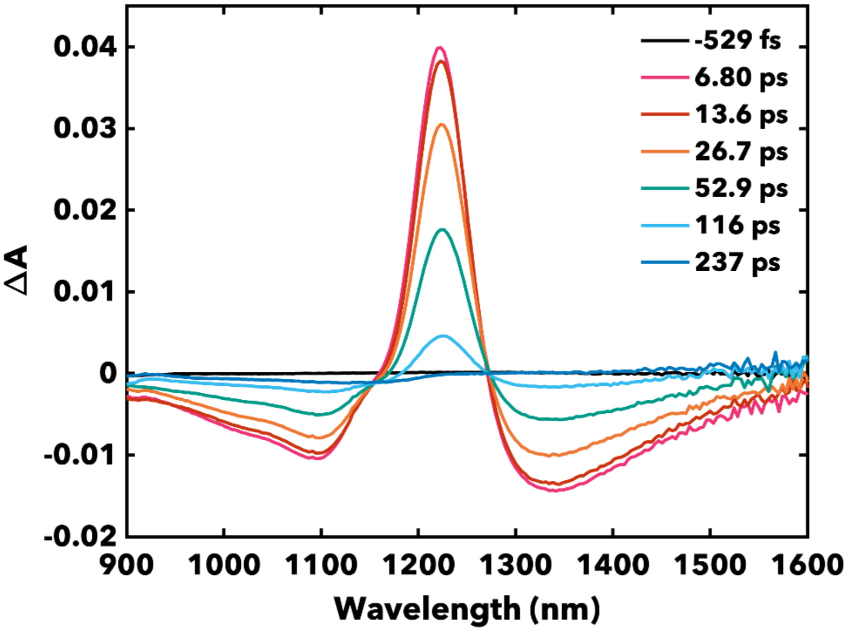

Figure S35. Decay of NIR transient absorption spectra of **1** in 1:1 DBrM:toluene at 298 K.

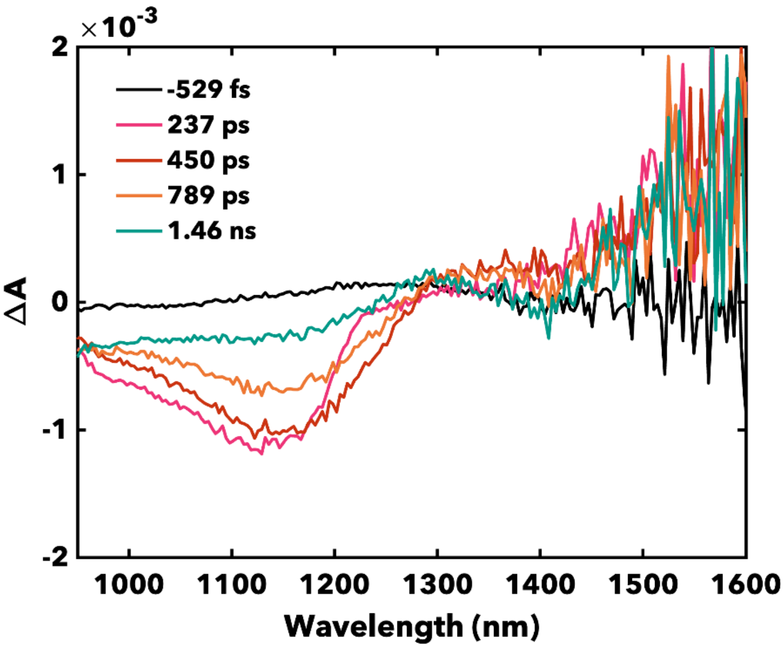

Figure S36. Decay of NIR transient absorption spectra of **1** in 1:1 DBrM:toluene at 298 K, at long timepoints.

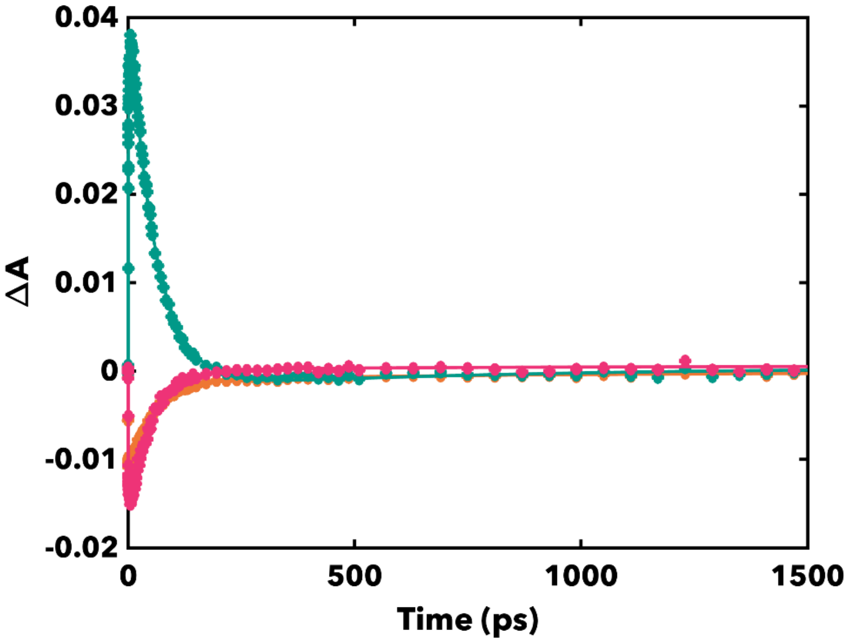

Figure S37. NIR transient kinetics of **1** in 1:1 DBrM:toluene at 298 K.

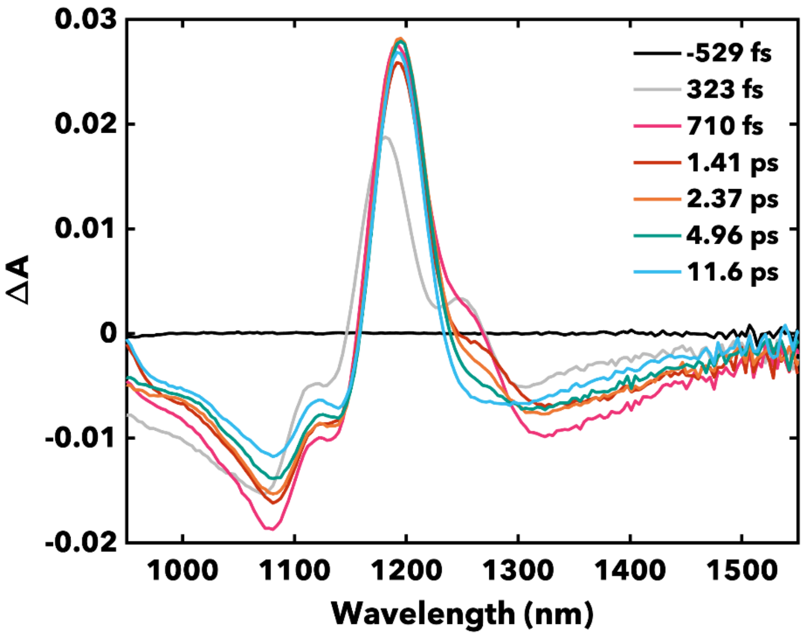

Figure S38. Growth of NIR transient absorption spectra of **1** in 1:1 DBrM:toluene at 100 K.

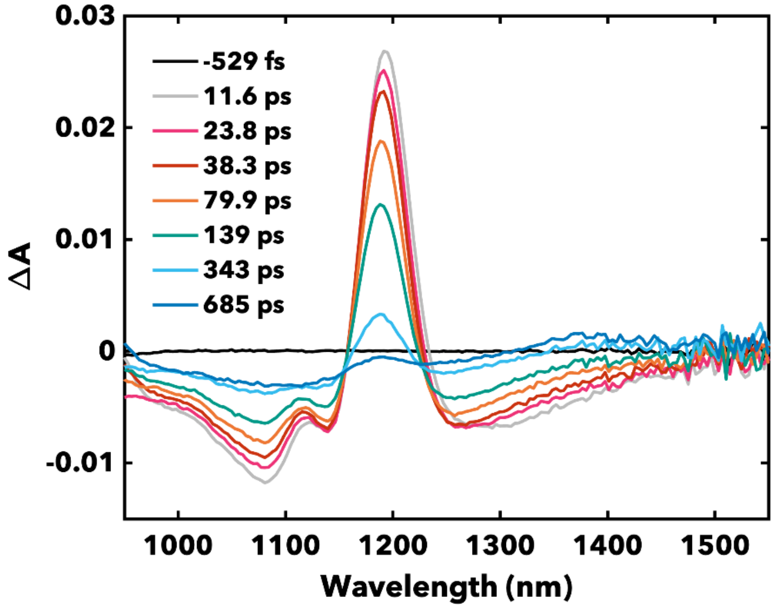

Figure S39. Decay of NIR transient absorption spectra of **1** in 1:1 DBrM:toluene at 100 K.

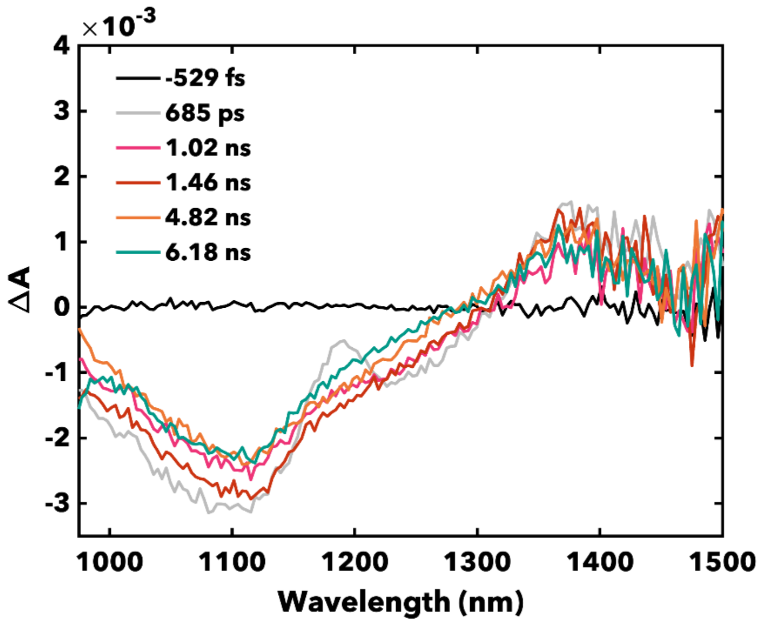

Figure S40. Decay of NIR transient absorption spectra of **1** in 1:1 DBrM:toluene at 100 K, at long timepoints.

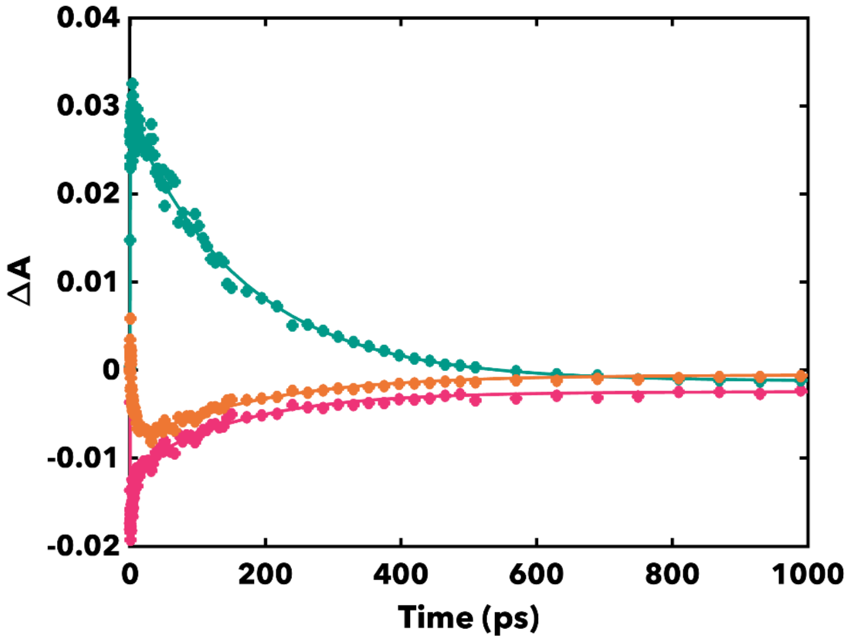

Figure S41. NIR transient kinetics of **1** in 1:1 DBrM:toluene at 100 K.

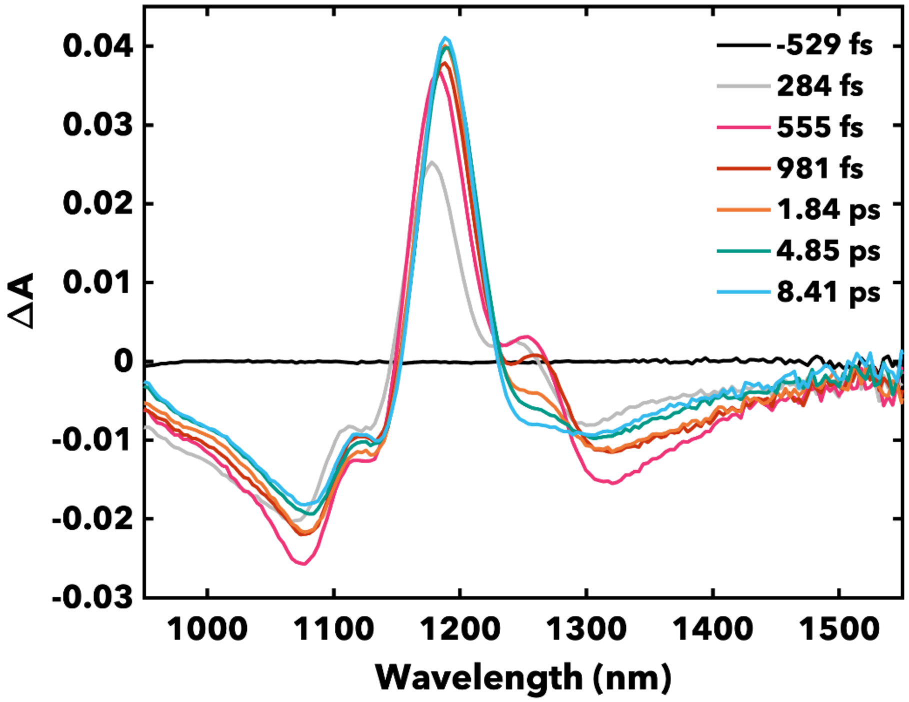

Figure S42. Growth of NIR transient absorption spectra of **1** in 1:1 DBrM:toluene at 2.9 K.

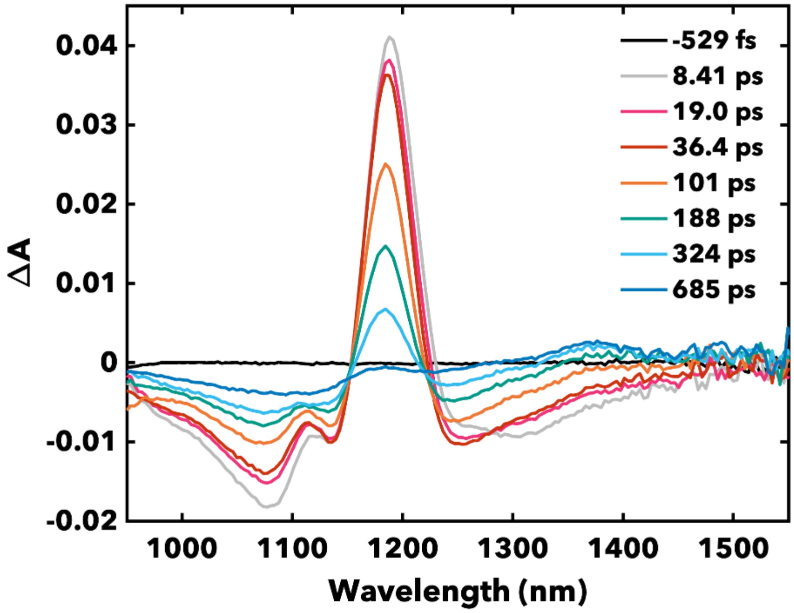

Figure S43. Decay of NIR transient absorption spectra of **1** in 1:1 DBrM:toluene at 2.9 K.

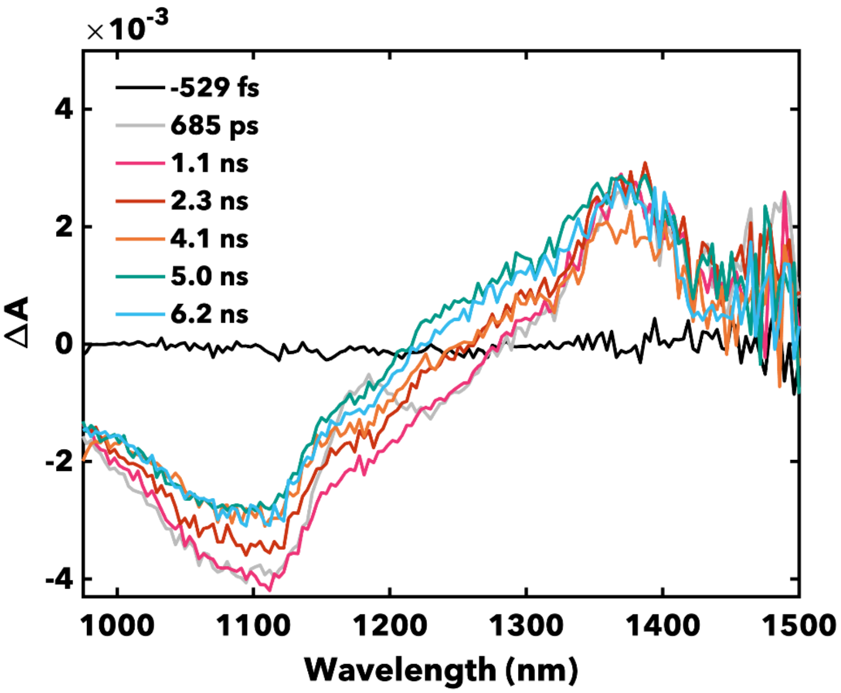

Figure S44. Decay of NIR transient absorption spectra of **1** in 1:1 DBrM:toluene at 2.9 K, at long timepoints.

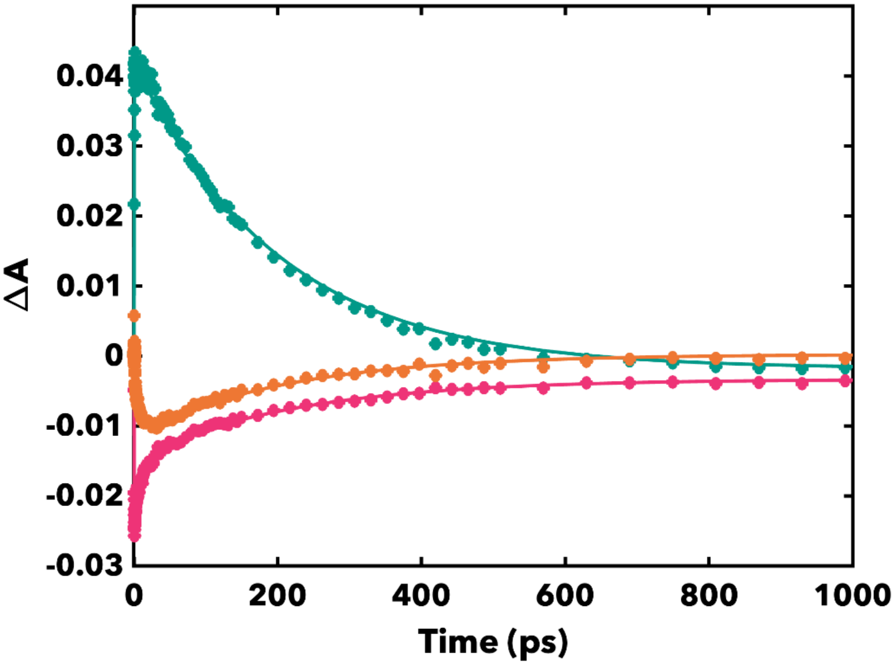

Figure S45. NIR transient kinetics of **1** in 1:1 DBrM:toluene at 2.9 K.

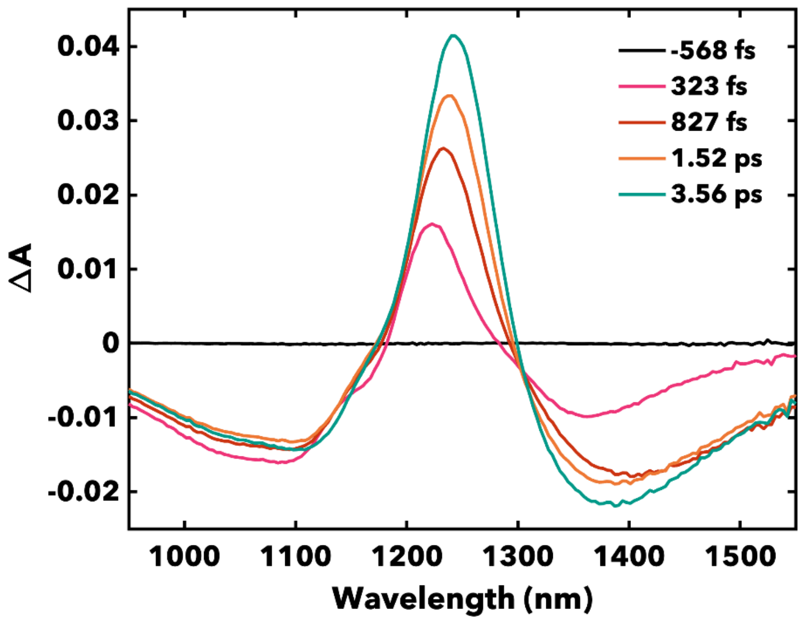

Figure S46. Growth of NIR transient absorption spectra of **2** in 1:1 DBrM:toluene at 298 K.

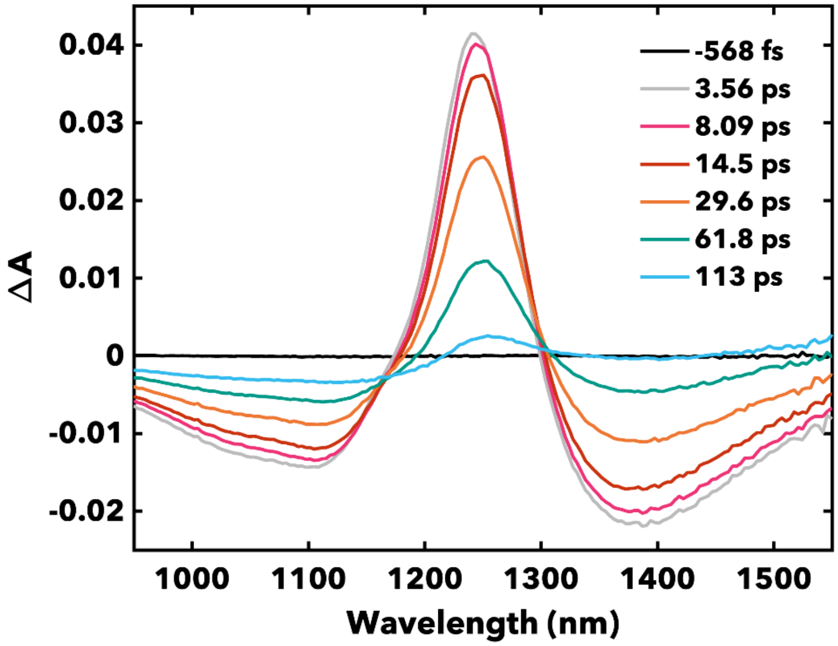

Figure S47. Decay of NIR transient absorption spectra of **2** in 1:1 DBrM:toluene at 298 K.

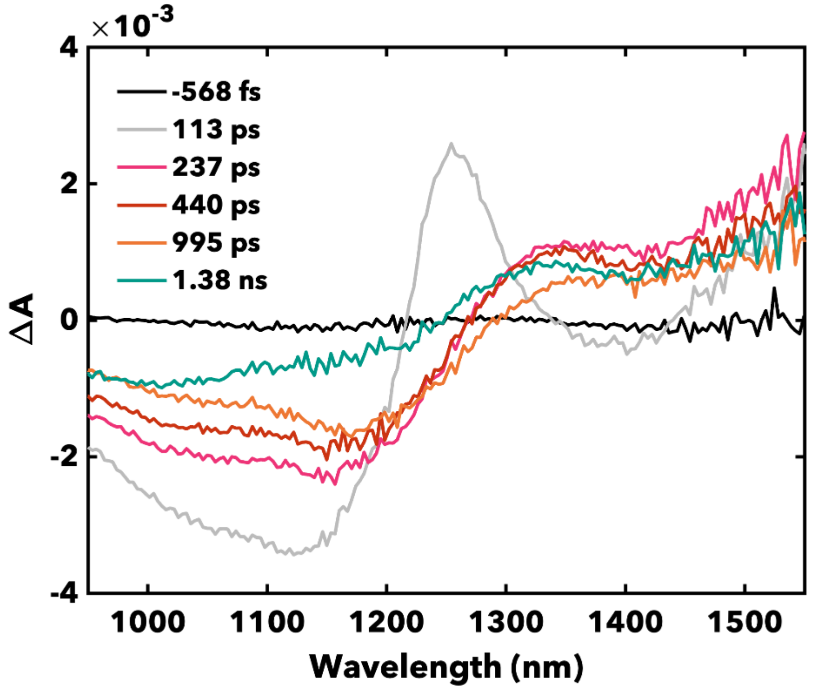

Figure S48. Decay of NIR transient absorption spectra of **2** in 1:1 DBrM:toluene at 298 K, at long timepoints.

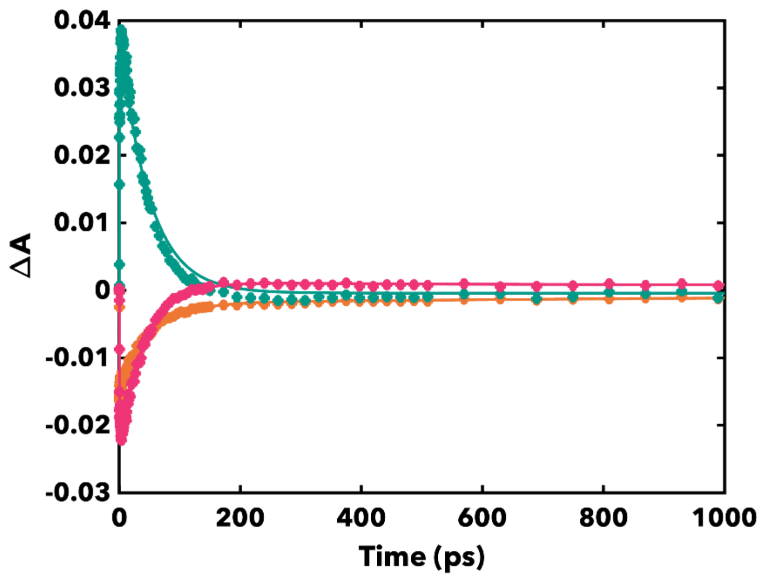

Figure S49. NIR transient kinetics of **2** in 1:1 DBrM:toluene at 298 K.

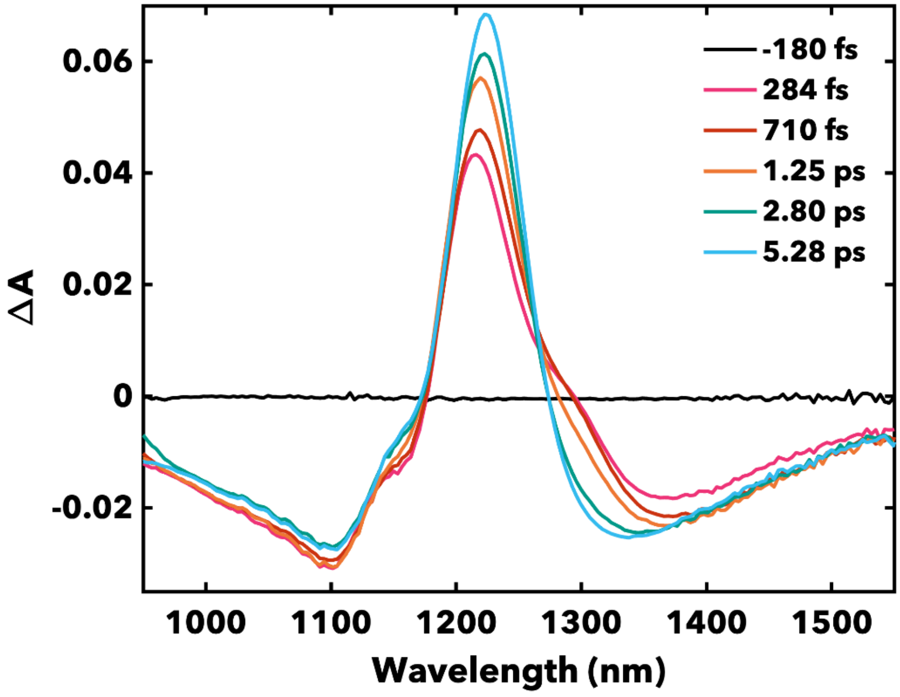

Figure S50. Growth of NIR transient absorption spectra of **2** in 1:1 DBrM:toluene at 195 K.

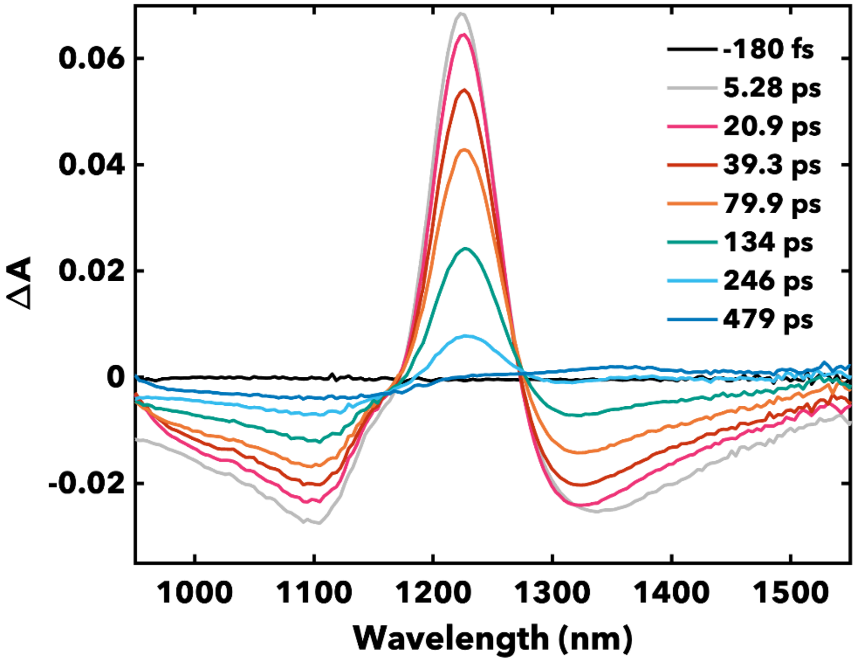

Figure S51. Decay of NIR transient absorption spectra of **2** in 1:1 DBrM:toluene at 195 K.

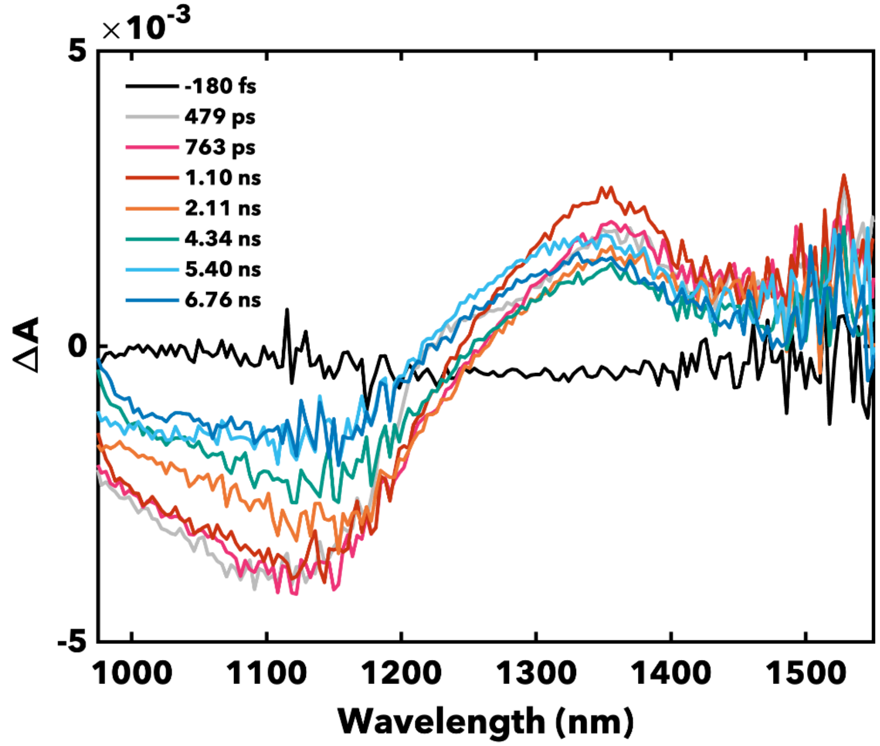

Figure S52. Decay of NIR transient absorption spectra of **2** in 1:1 DBrM:toluene at 195 K, at long timepoints.

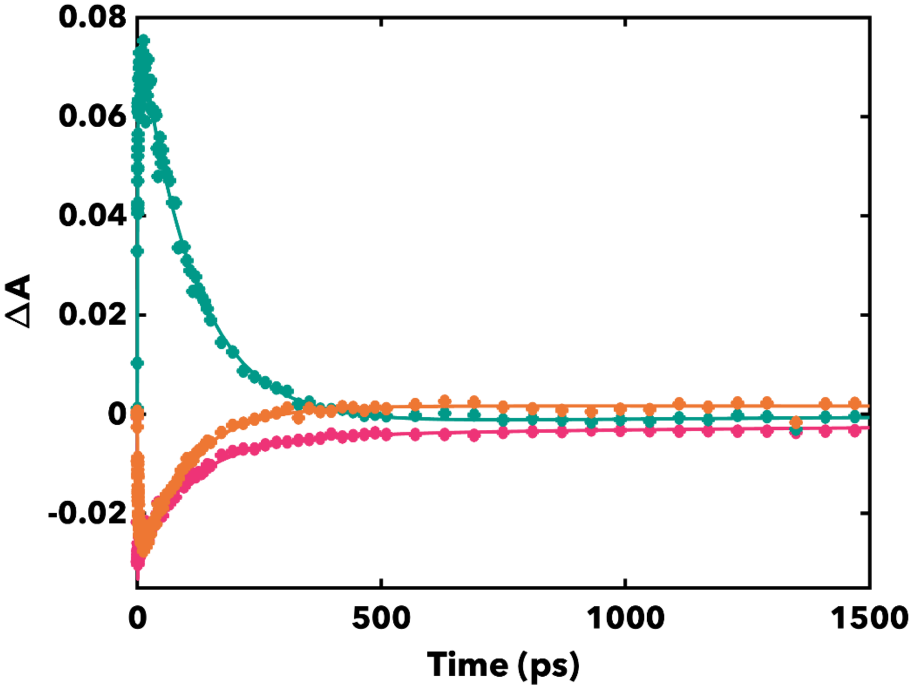

Figure S53. NIR transient kinetics of **2** in 1:1 DBrM:toluene at 195 K.

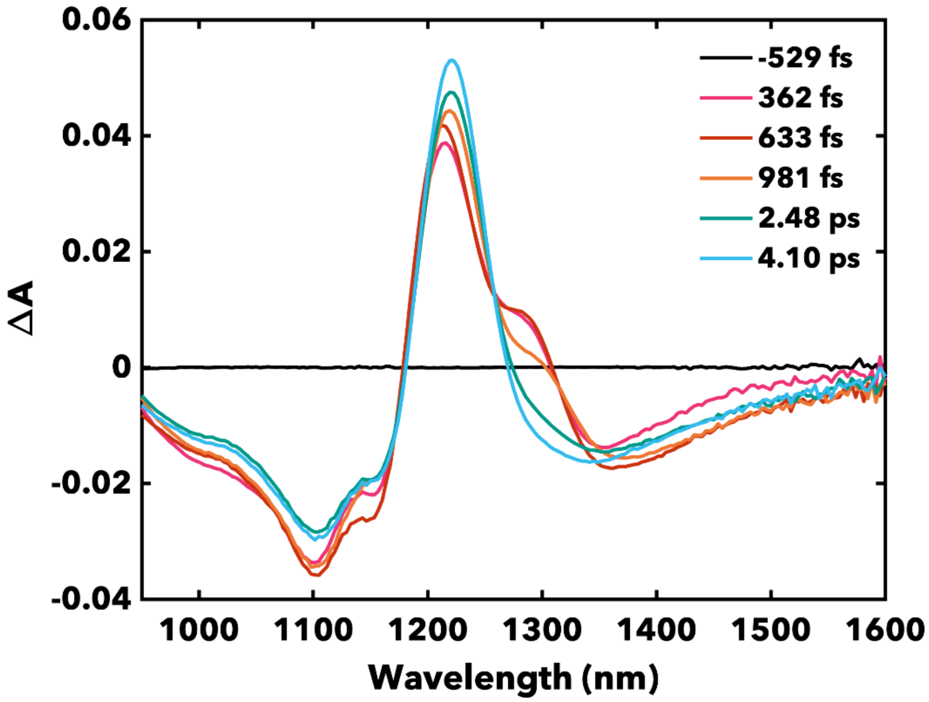

Figure S54. Growth of NIR transient absorption spectra of **2** in 1:1 DBrM:toluene at 100 K.

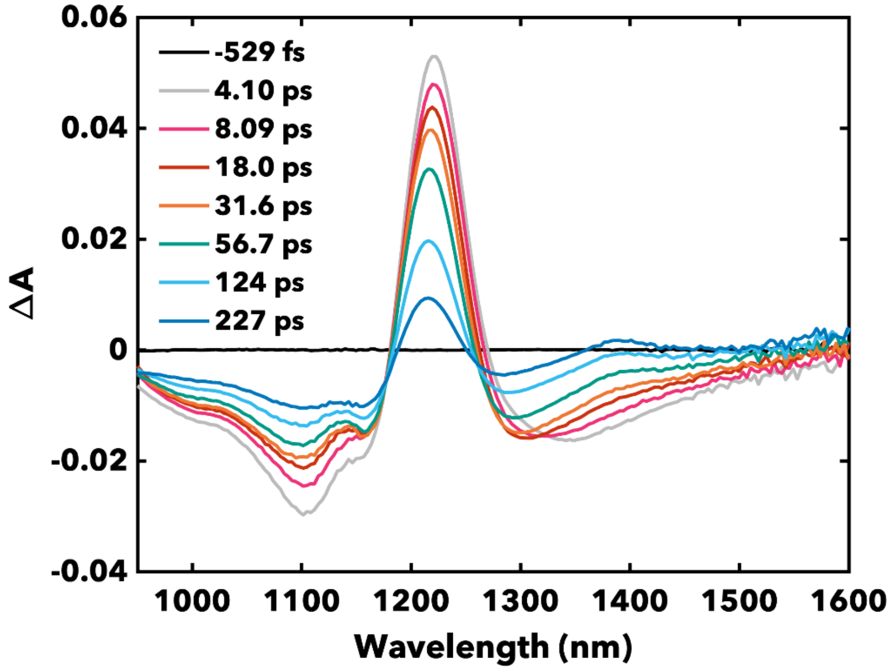

Figure S55. Decay of NIR transient absorption spectra of **2** in 1:1 DBrM:toluene at 100 K.

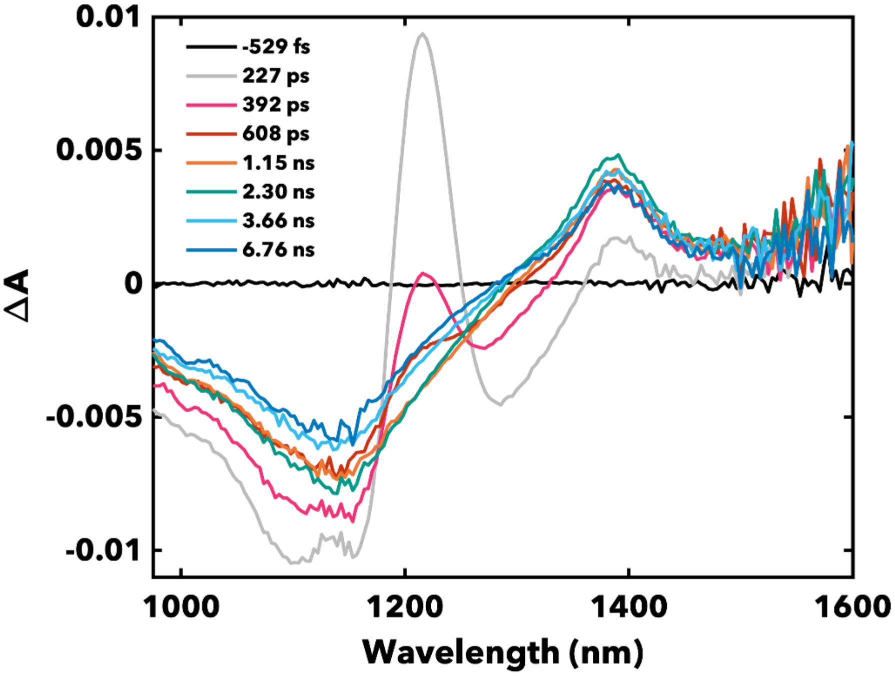

Figure S56. Decay of NIR transient absorption spectra of **2** in 1:1 DBrM:toluene at 100 K, at long timepoints.

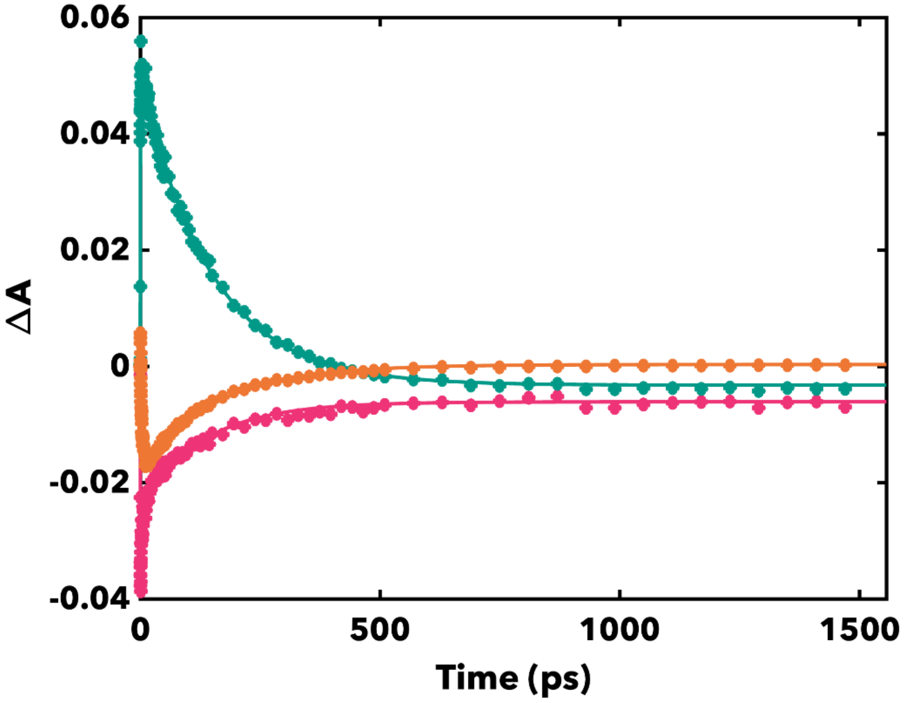

Figure S57. NIR transient kinetics of **2** in 1:1 DBrM:toluene at 100 K.

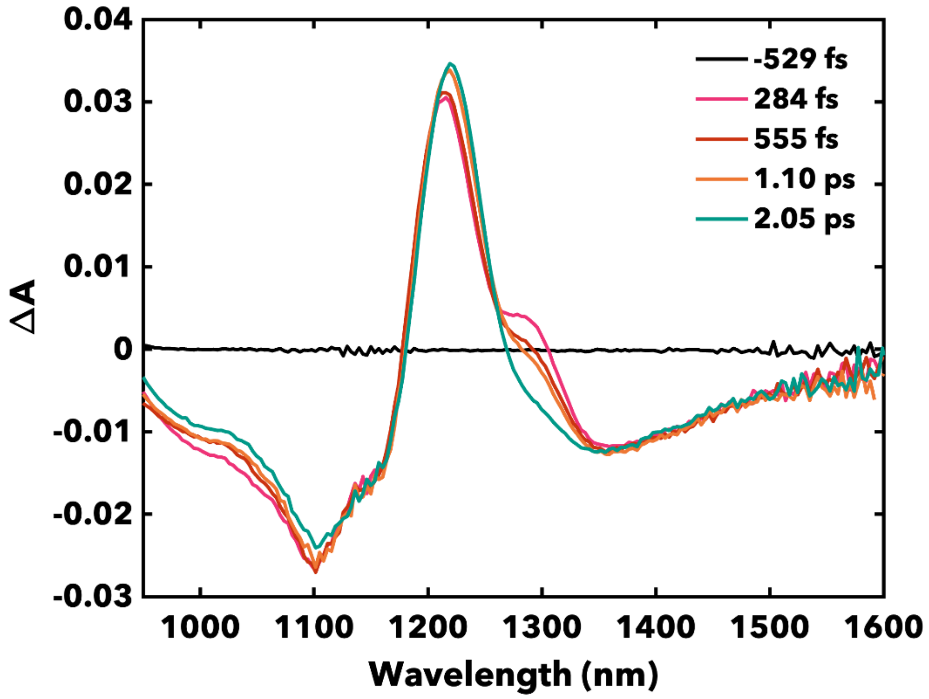

Figure S58. Growth of NIR transient absorption spectra of **2** in 1:1 DBrM:toluene at 50 K.

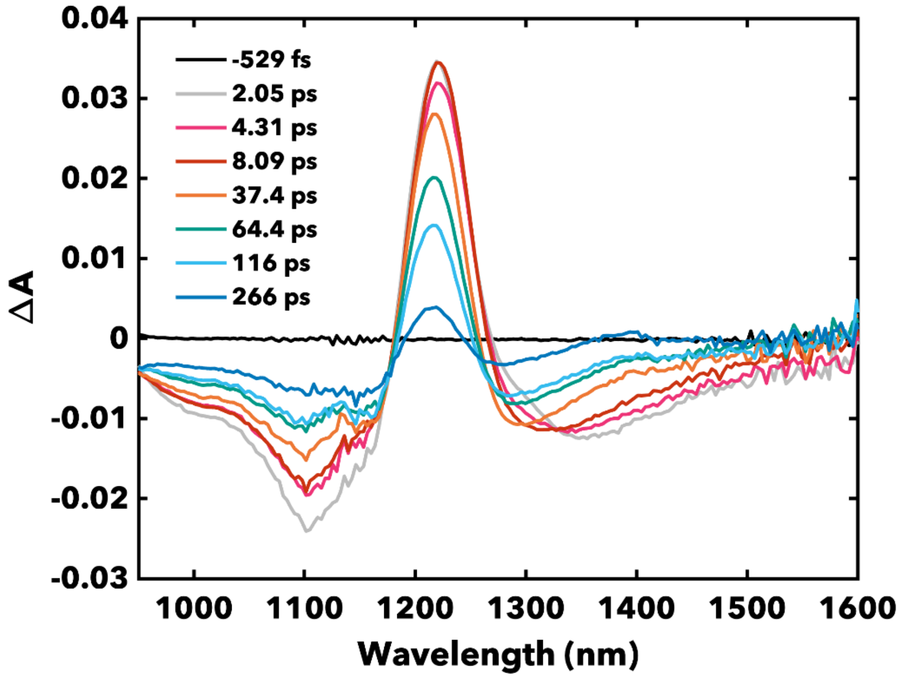

Figure S59. Decay of NIR transient absorption spectra of **2** in 1:1 DBrM:toluene at 50 K.

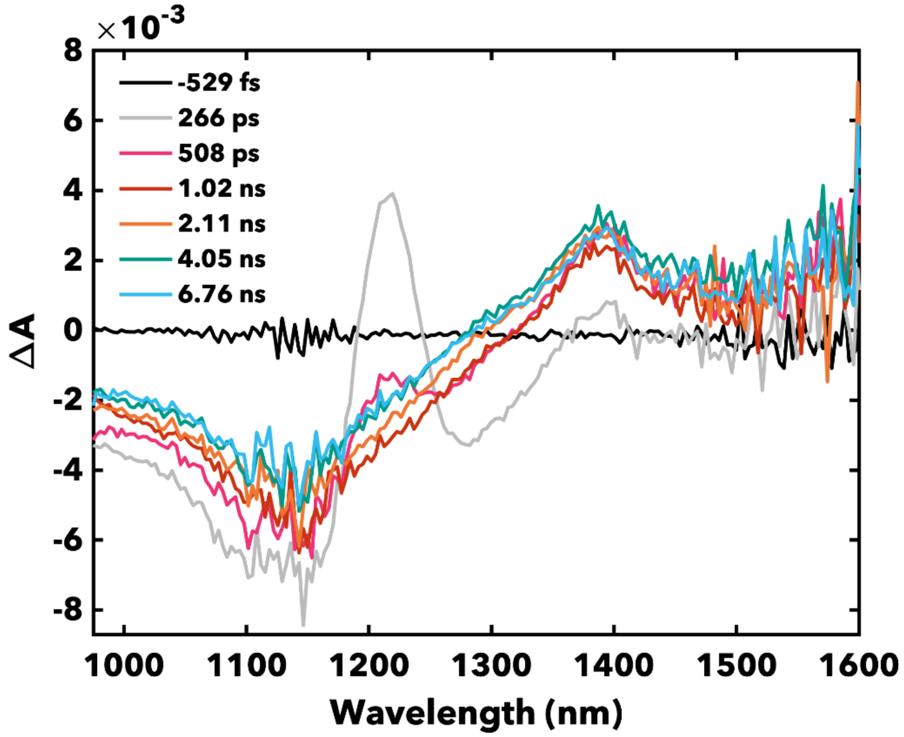

Figure S60. Decay of NIR transient absorption spectra of **2** in 1:1 DBrM:toluene at 50 K, at long timepoints.

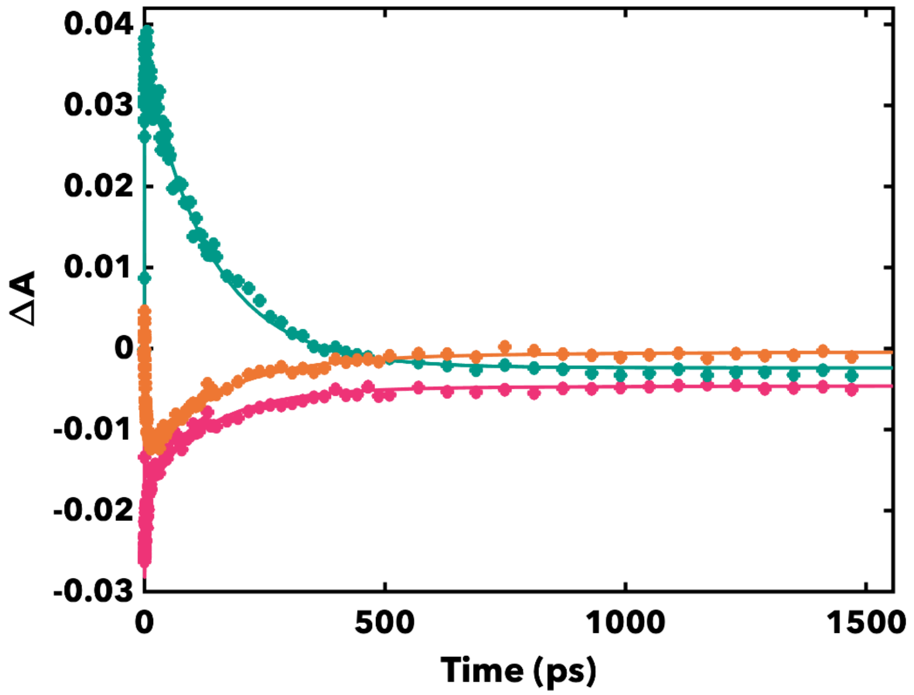

Figure S61. NIR transient kinetics of **2** in 1:1 DBrM:toluene at 50 K.

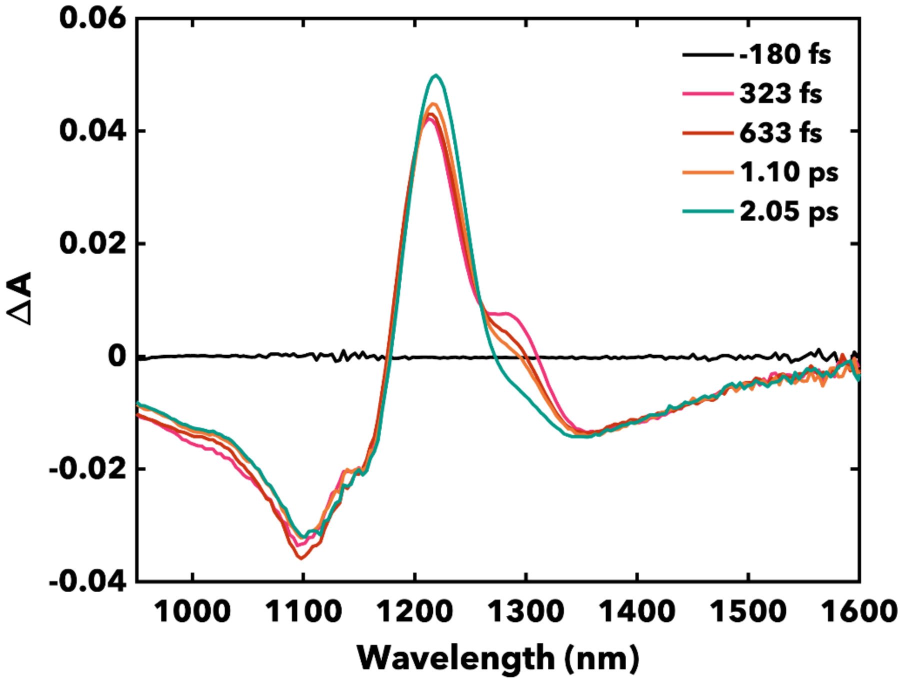

Figure S62. Growth of NIR transient absorption spectra of **2** in 1:1 DBrM:toluene at 10 K.

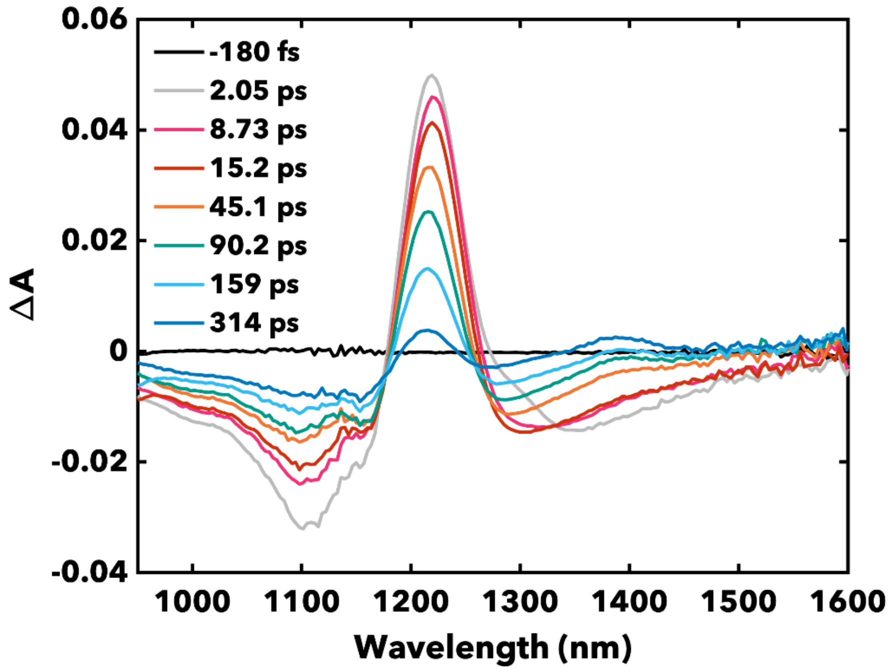

Figure S63. Decay of NIR transient absorption spectra of **2** in 1:1 DBrM:toluene at 10 K.

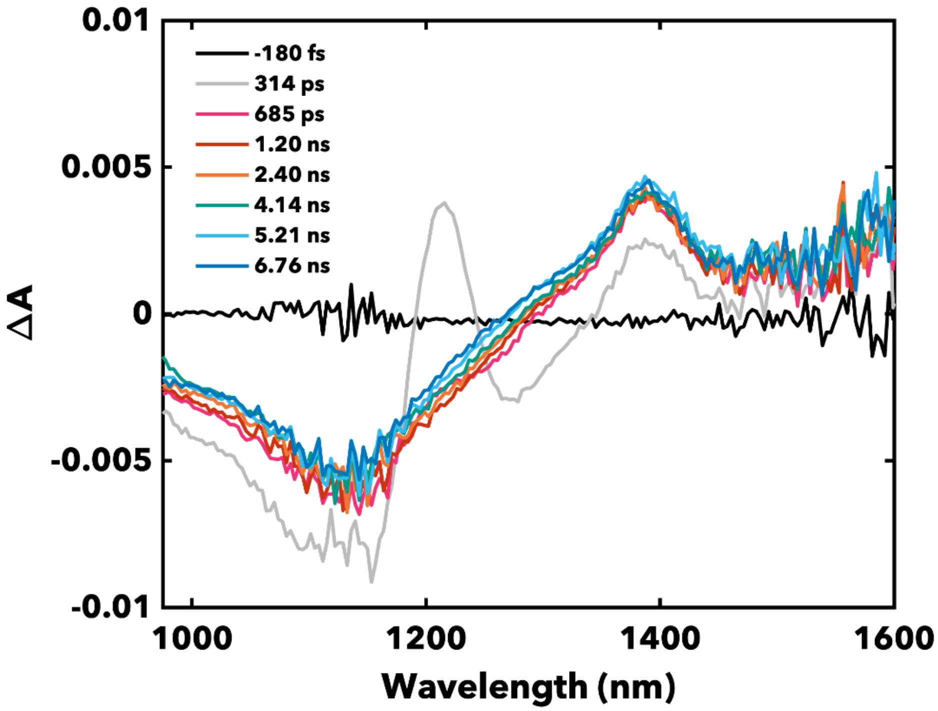

Figure S64. Decay of NIR transient absorption spectra of **2** in 1:1 DBrM:toluene at 10 K, at long timepoints.

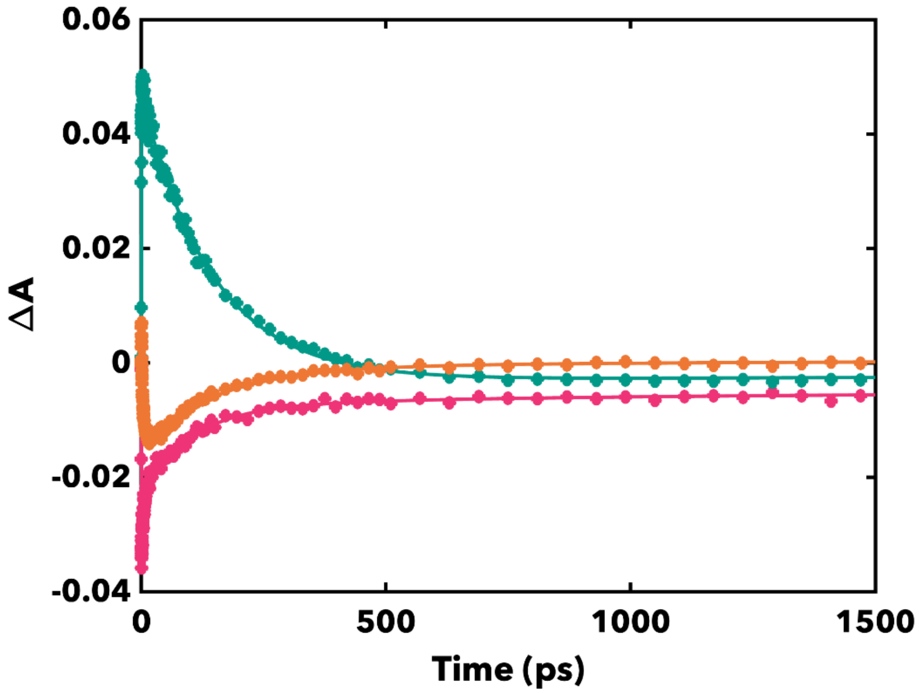

Figure S65. NIR transient kinetics of **2** in 1:1 DBrM:toluene at 10 K.

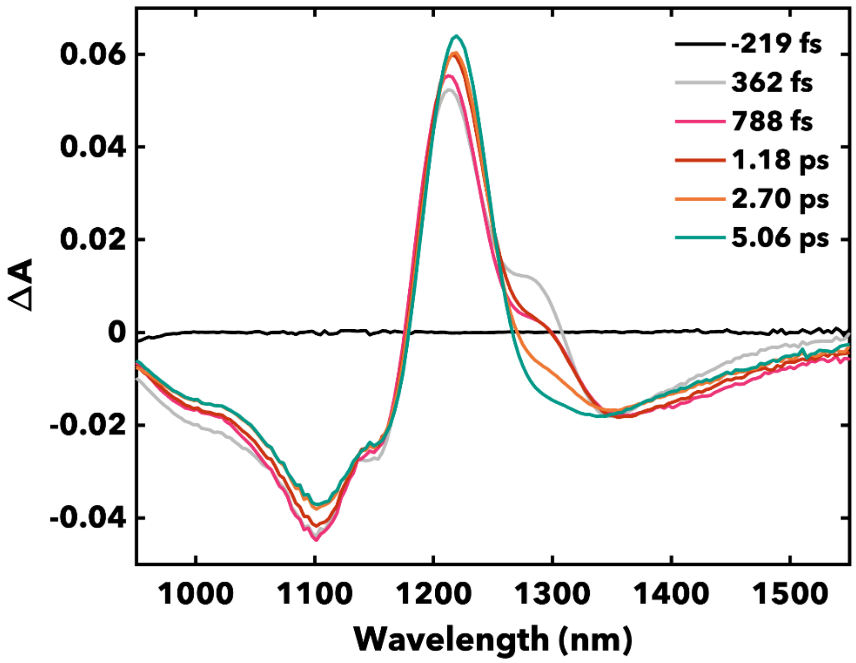

Figure S66. Growth of NIR transient absorption spectra of **2** in 1:1 DBrM:toluene at 2.9 K.

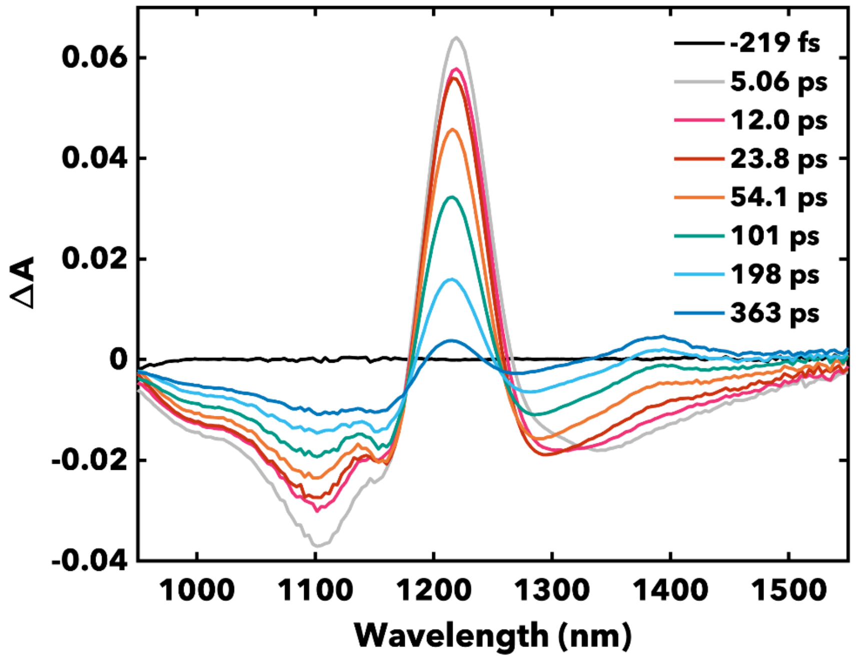

Figure S67. Decay of NIR transient absorption spectra of **2** in 1:1 DBrM:toluene at 2.9 K.

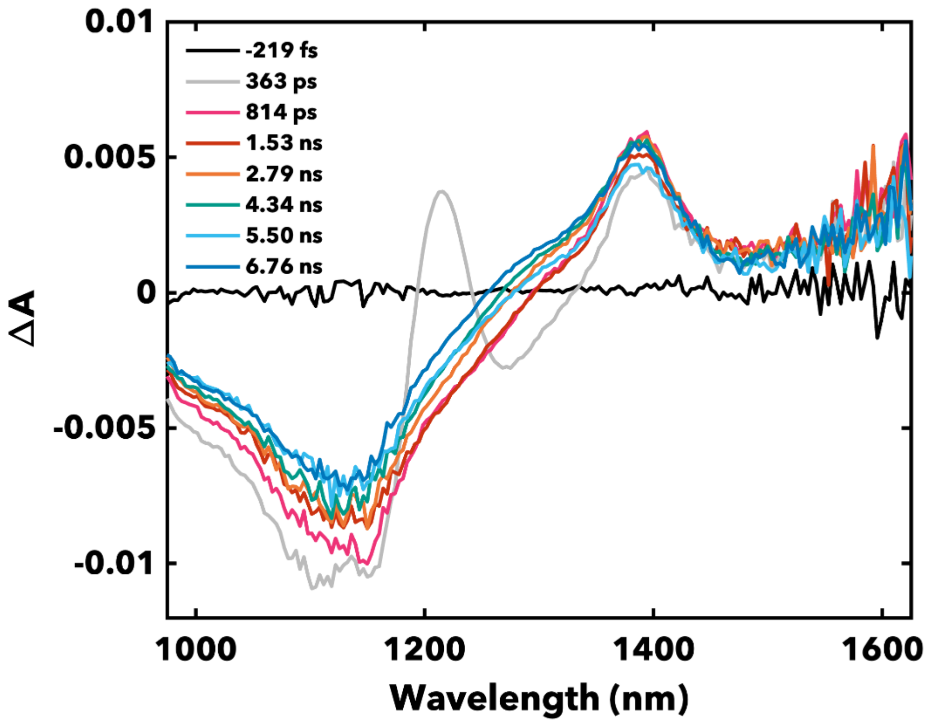

Figure S68. Decay of NIR transient absorption spectra of **2** in 1:1 DBrM:toluene at 2.9 K, at long timepoints.

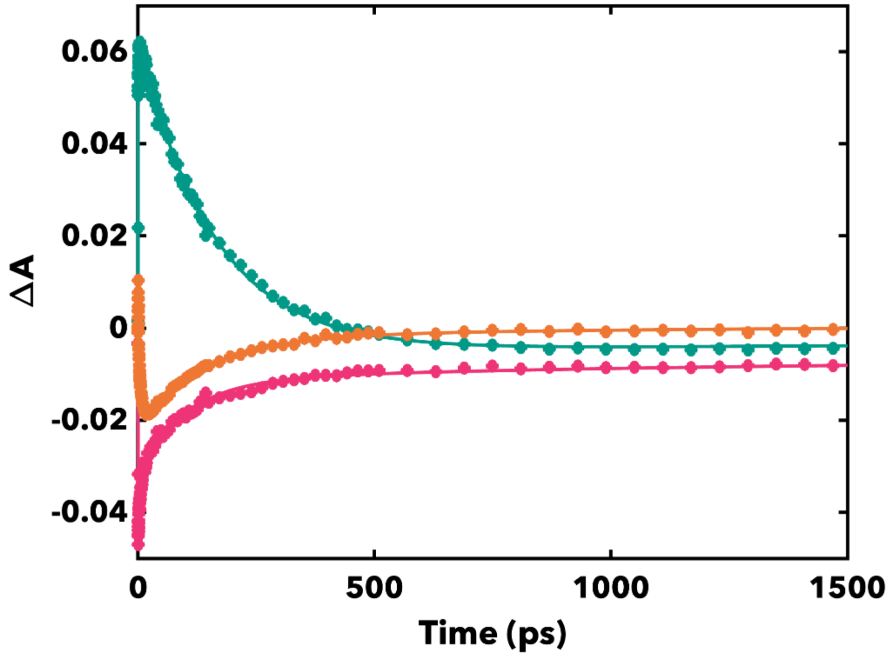

Figure S69. NIR transient kinetics of **2** in 1:1 DBrM:toluene at 2.9 K.

2. TCSPC lifetime determination

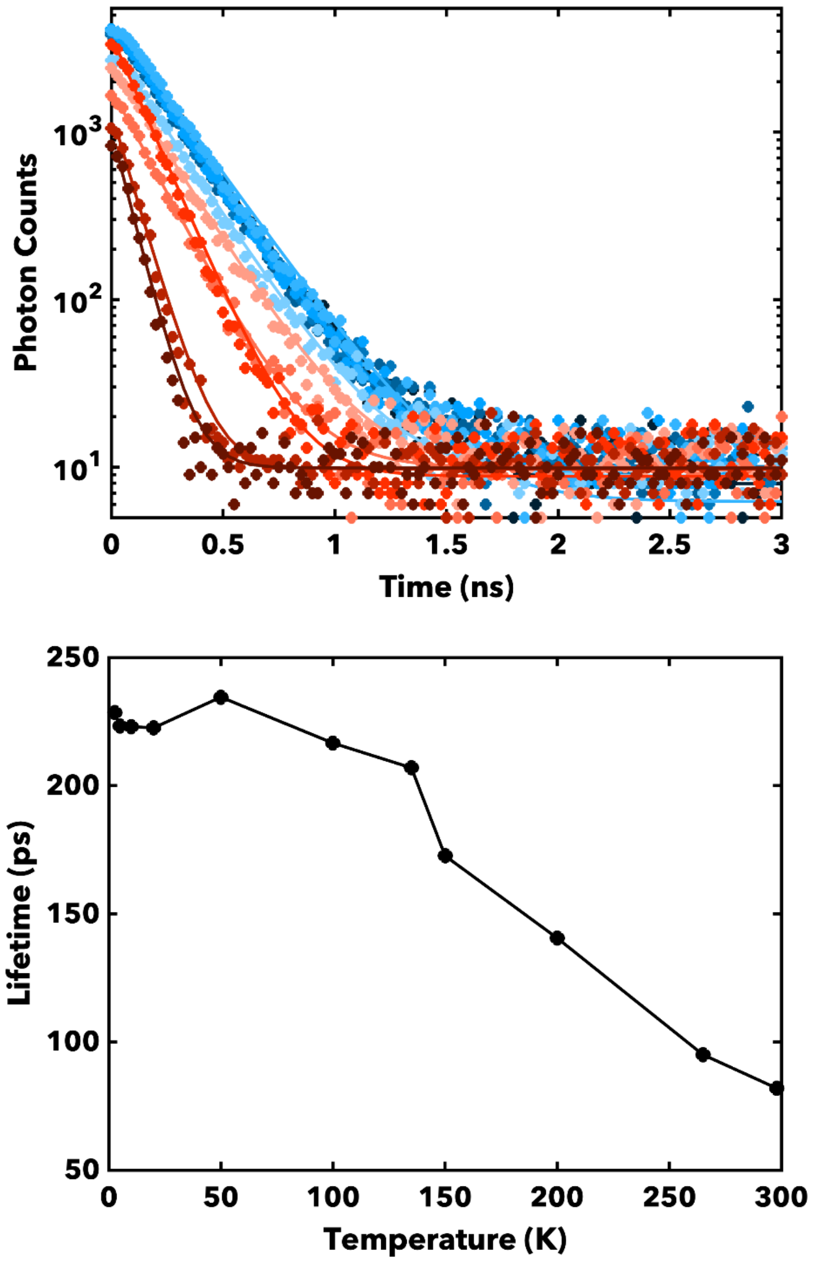

Figure S70. Photoluminescence decay (upper) and fit lifetimes (lower) of **1** in 1:1 DBrM:toluene from 298 K (red) to 2.8 K (blue).

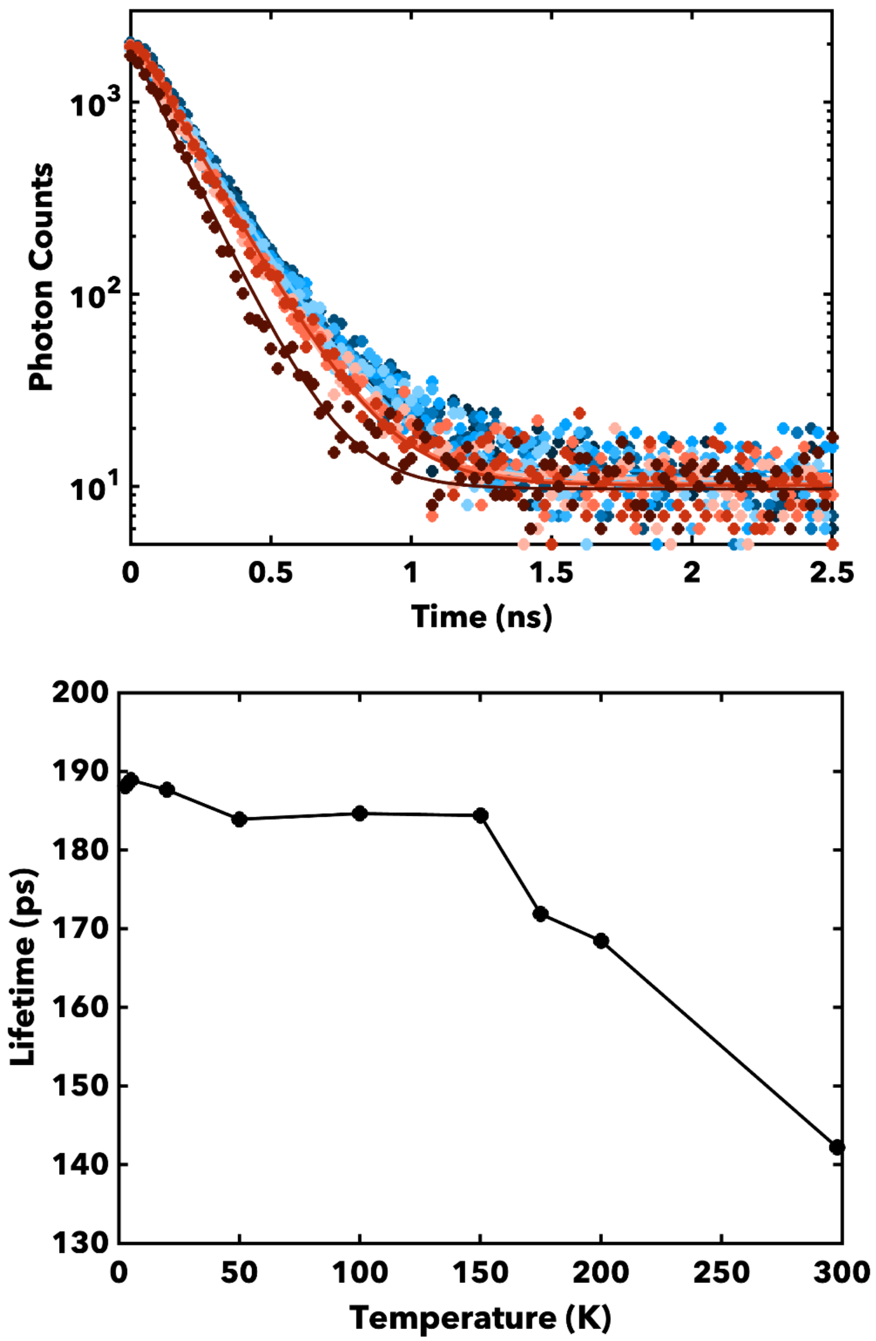

Figure S71. Photoluminescence decay (upper) and fit lifetimes (lower) of **1** in polymer from 298 K (red) to 2.8 K (blue). Polymer used is (poly(butyl methacrylate-co-isobutyl methacrylate).

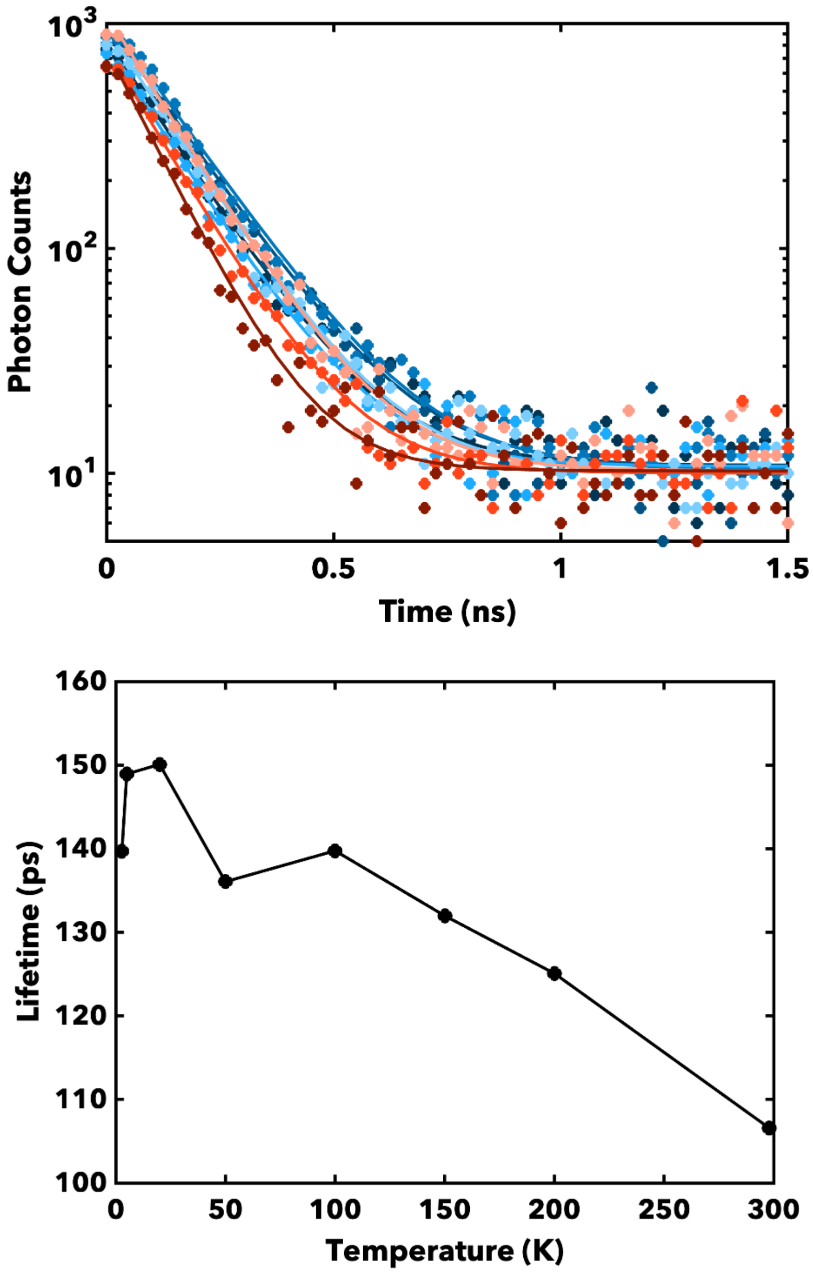

Figure S72. Photoluminescence decay (upper) and fit lifetimes (lower) of **2** in polymer from 298 K (red) to 2.8 K (blue).

3. Variable temperature photoluminescence

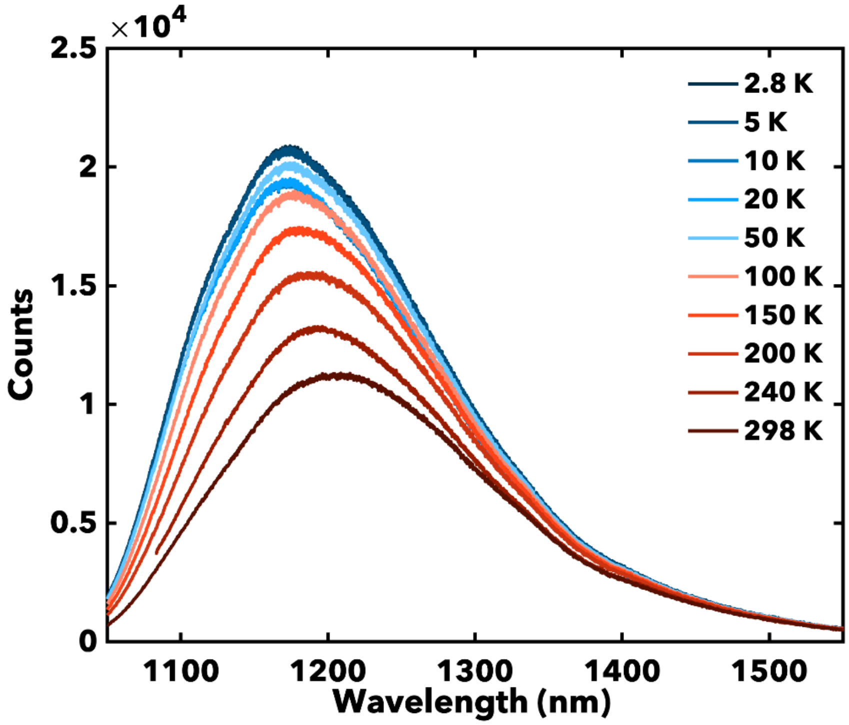

Figure S73. Photoluminescence across temperatures of **1** in polymer.

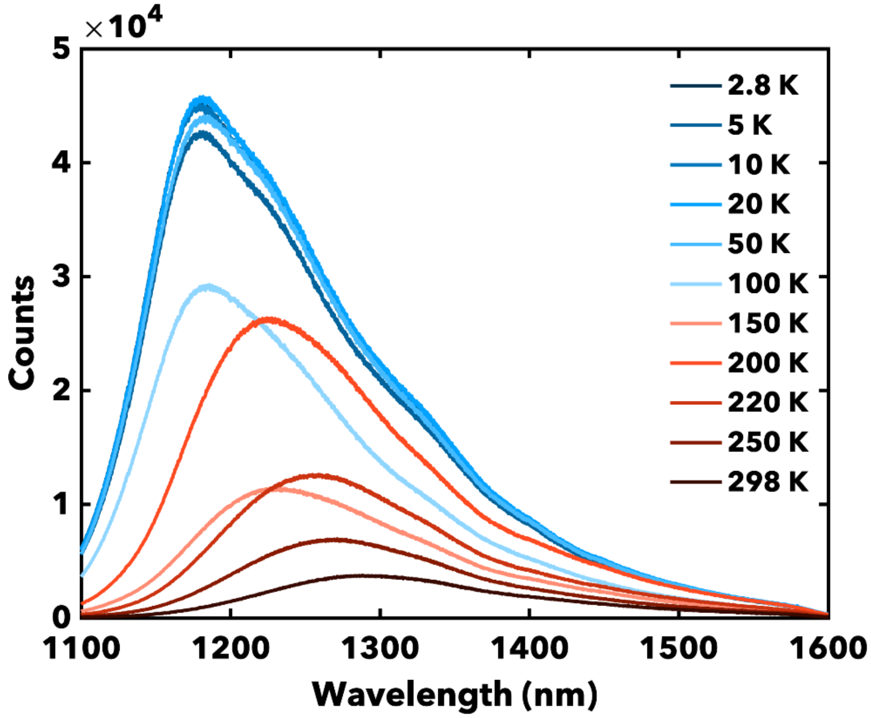

Figure S74. Photoluminescence across temperatures of **1** in 1:1 DBrM:toluene.

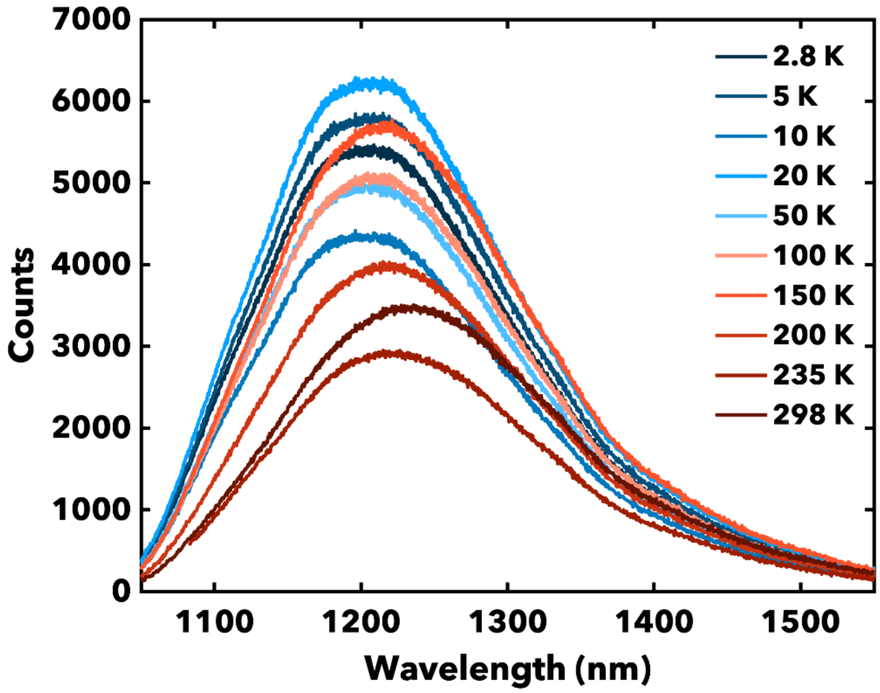

Figure S75. Photoluminescence across temperatures of **2** in polymer.

4. Stability and solvatochromism experiments

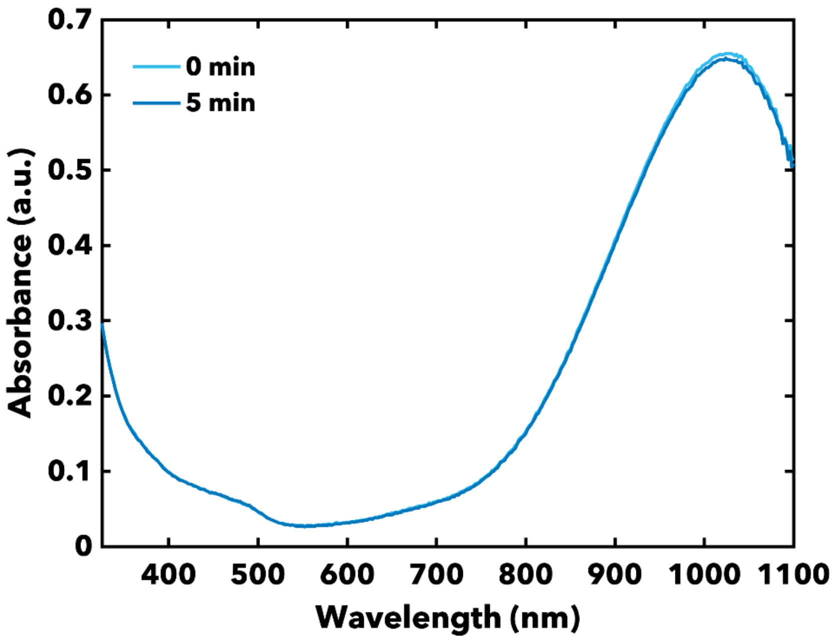

Figure S76. Stability of **1** in MeCN in air, at 298 K.

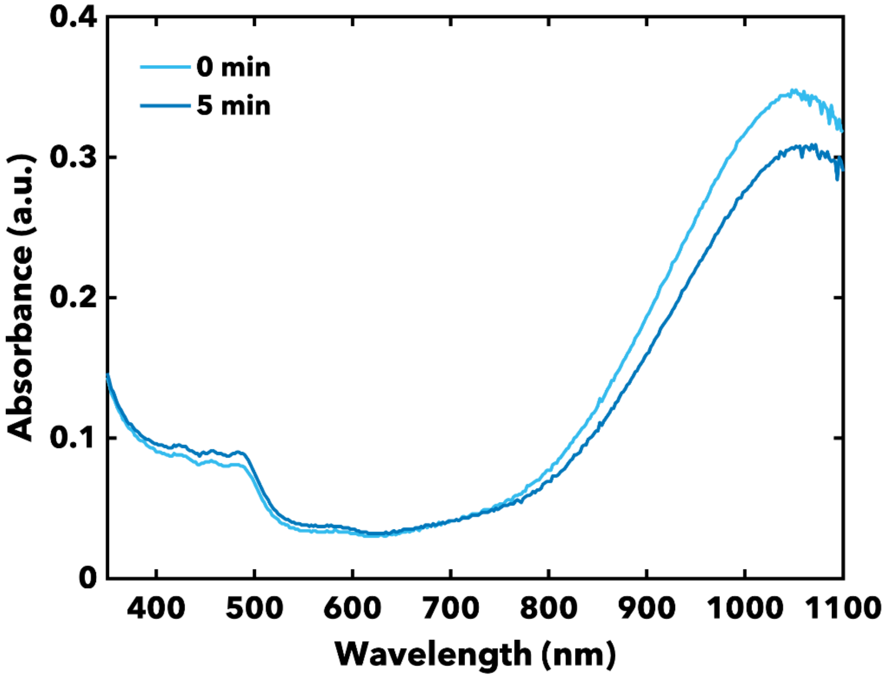

Figure S77. Stability of **1** in acetone in air, at 298 K.

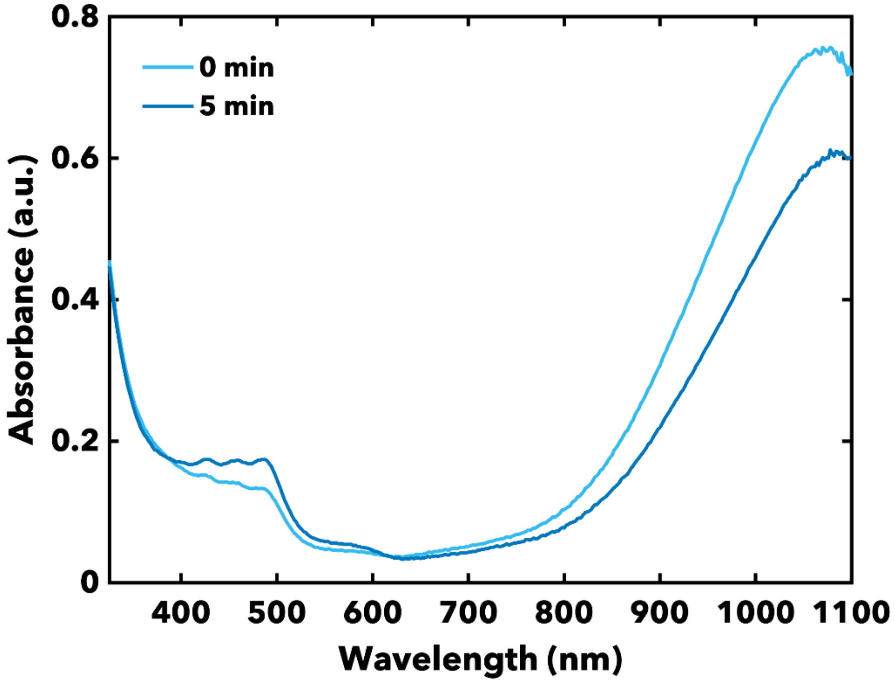

Figure S78. Stability of **1** in THF in air, at 298 K.

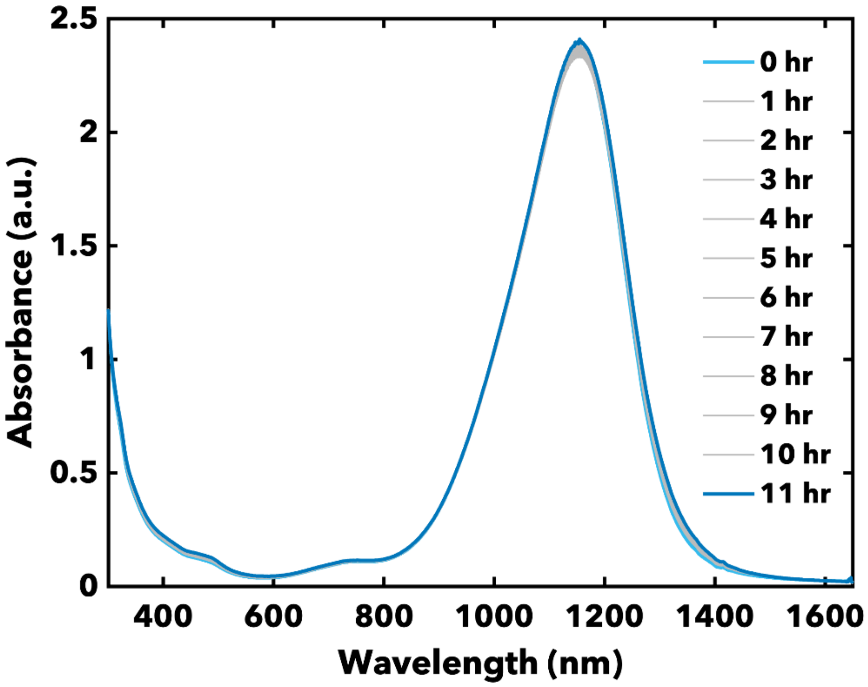

Figure S79. Stability of **1** in DCM in air, at 298 K.

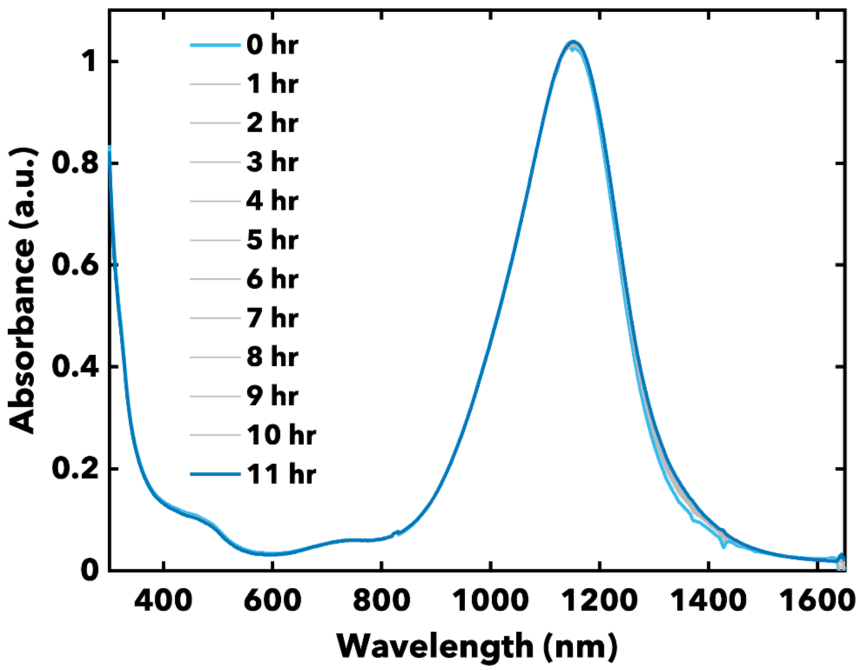

Figure S80. Stability of **1** in 1:1 DBrM:toluene in air, at 298 K.

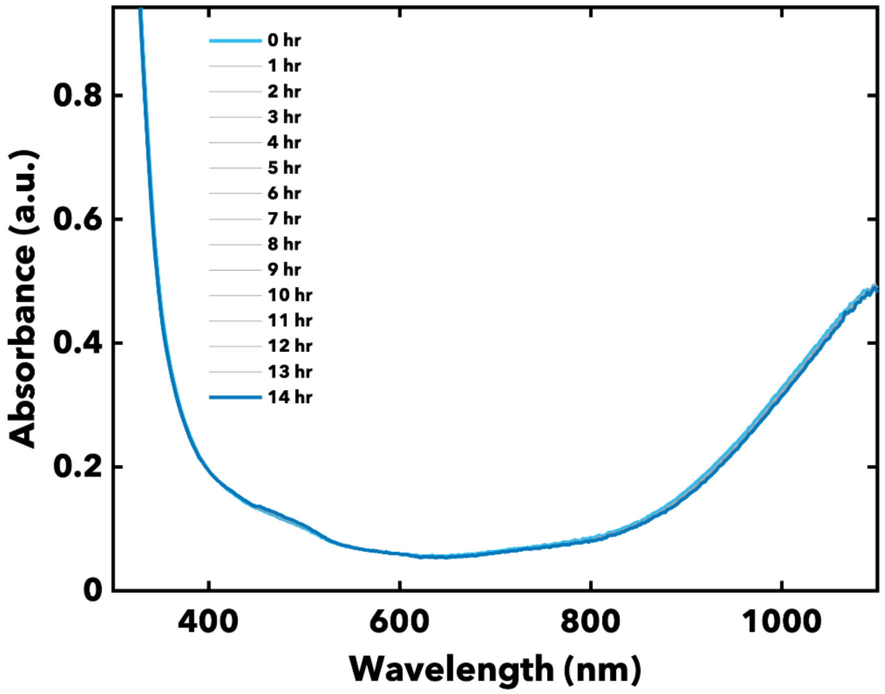

Figure S81. Stability of **2** in 1:40 DMSO:water, in air, at 298 K.

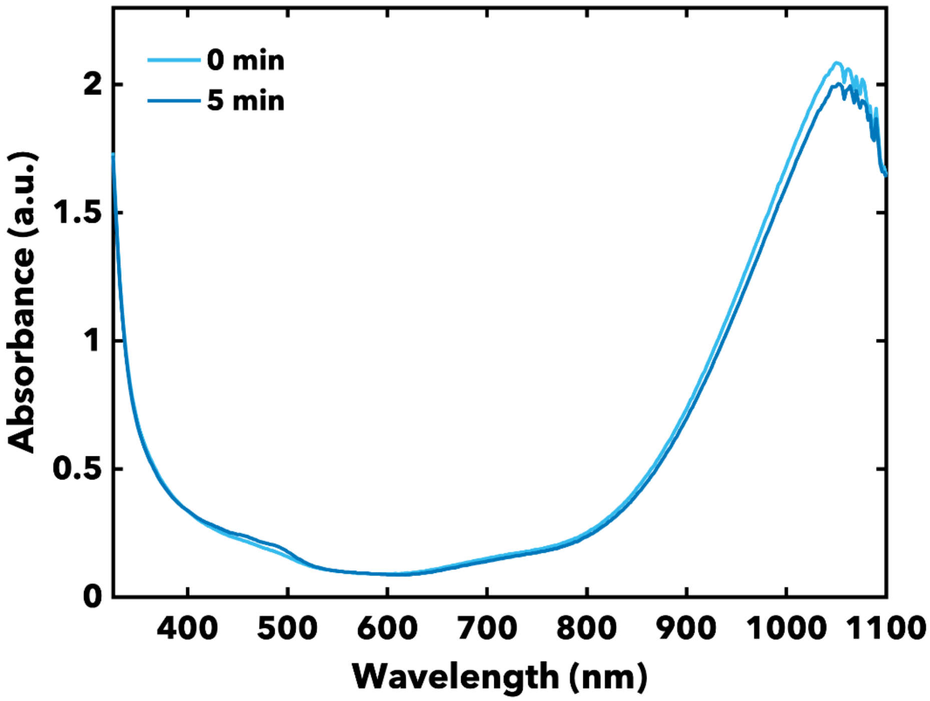

Figure S82. Stability of **2** in THF in air, at 298 K.

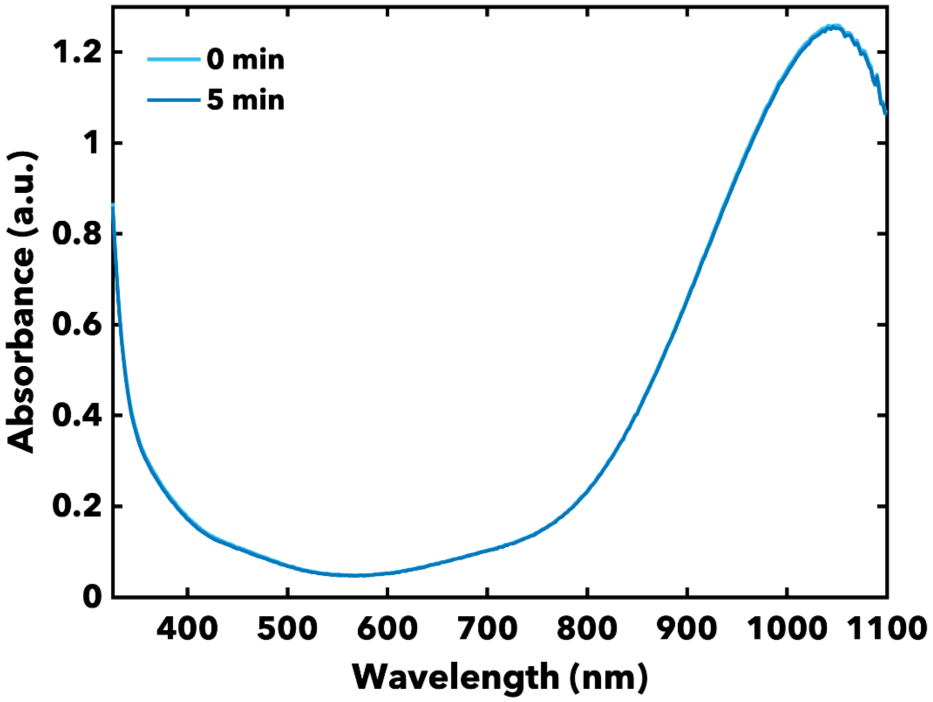

Figure S83. Stability of **2** in MeCN in air, at 298 K.

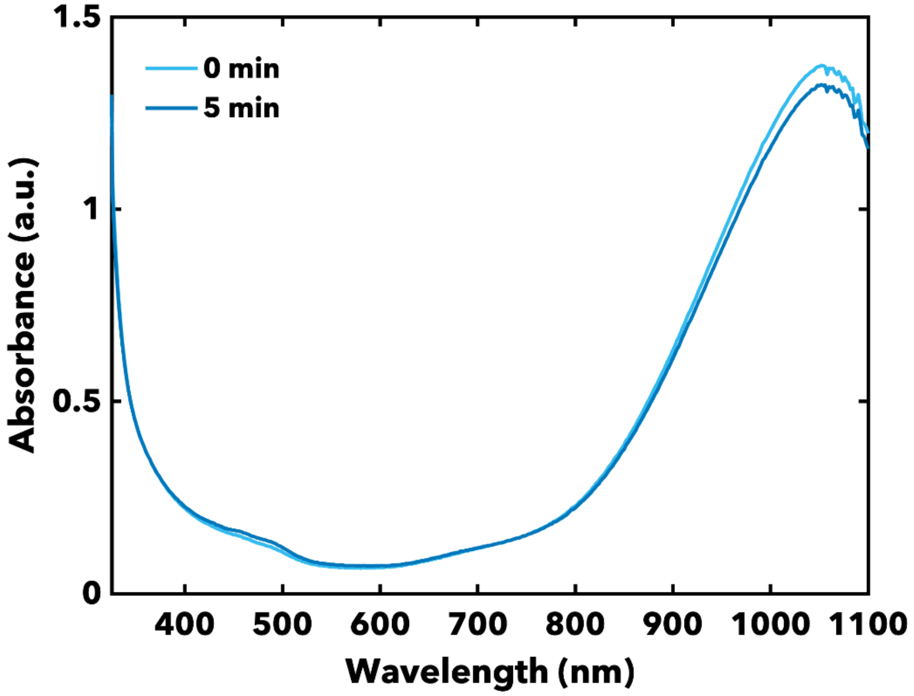

Figure S84. Stability of **2** in acetone in air, at 298 K.

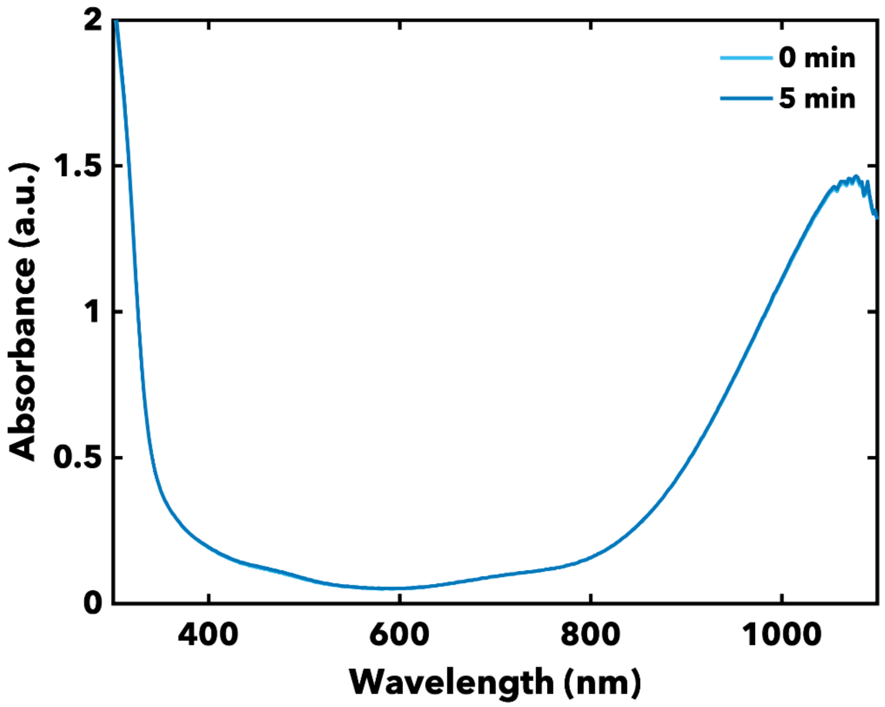

Figure S85. Stability of **2** in glacial acetic acid in air, at 298 K.

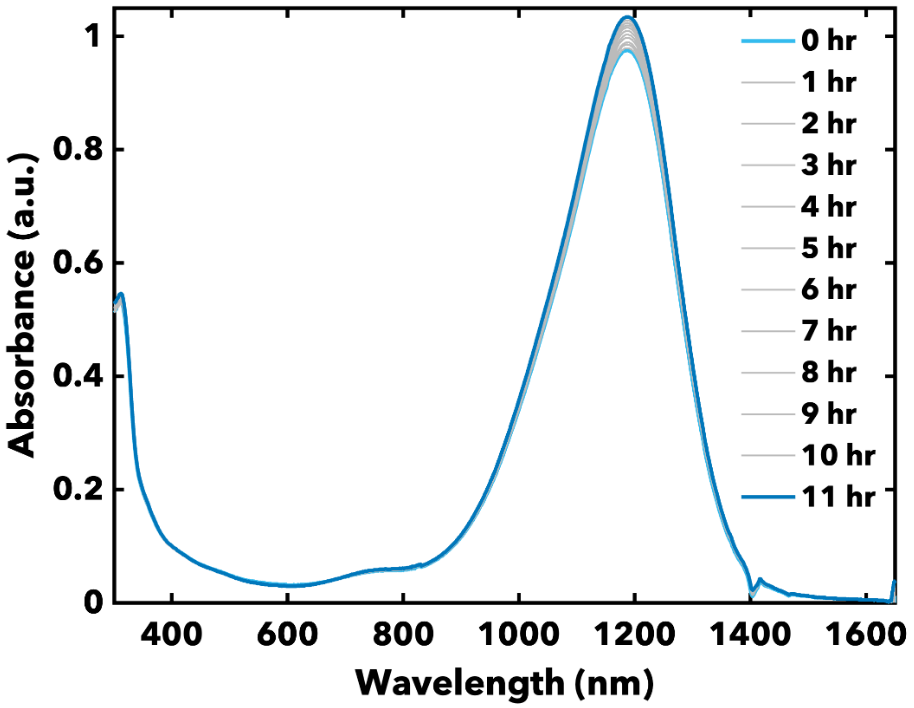

Figure S86. Stability of **2** in DCM in air, at 298 K.

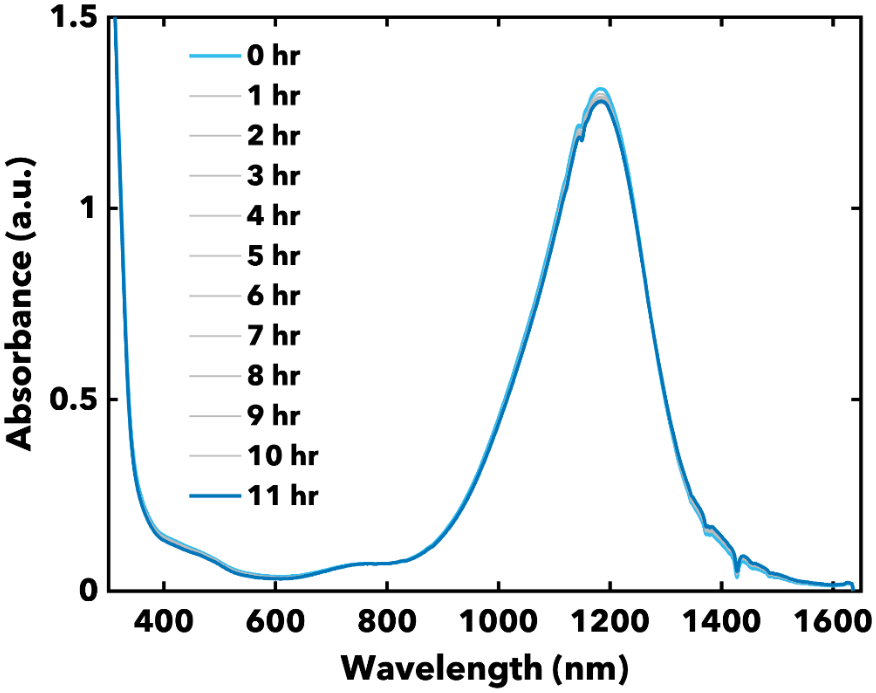

Figure S87. Stability of **2** in 1:1 DBrM:toluene in air, at 298 K.

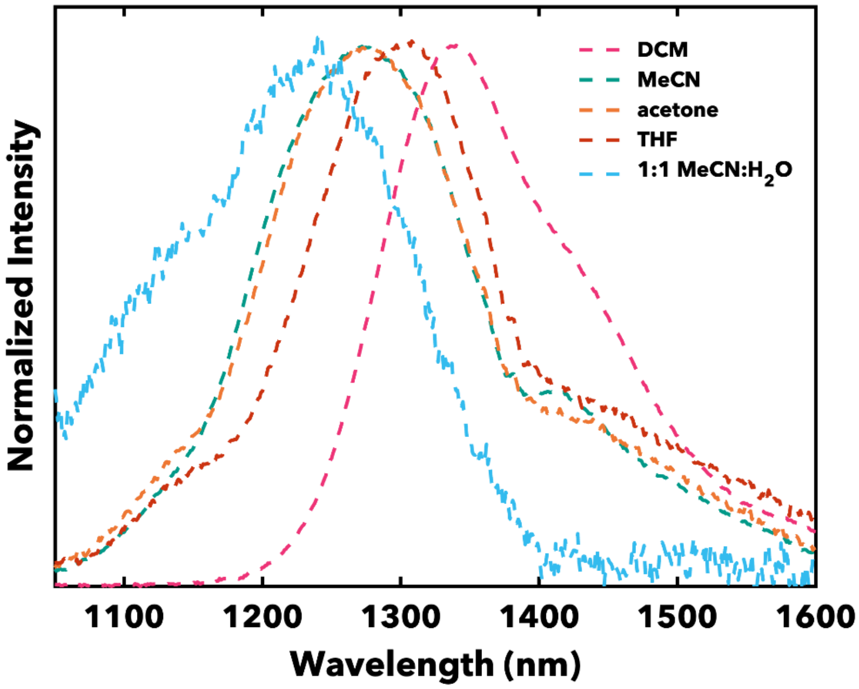

Figure S88. Solvatochromism of **1** across solvents at 298 K.

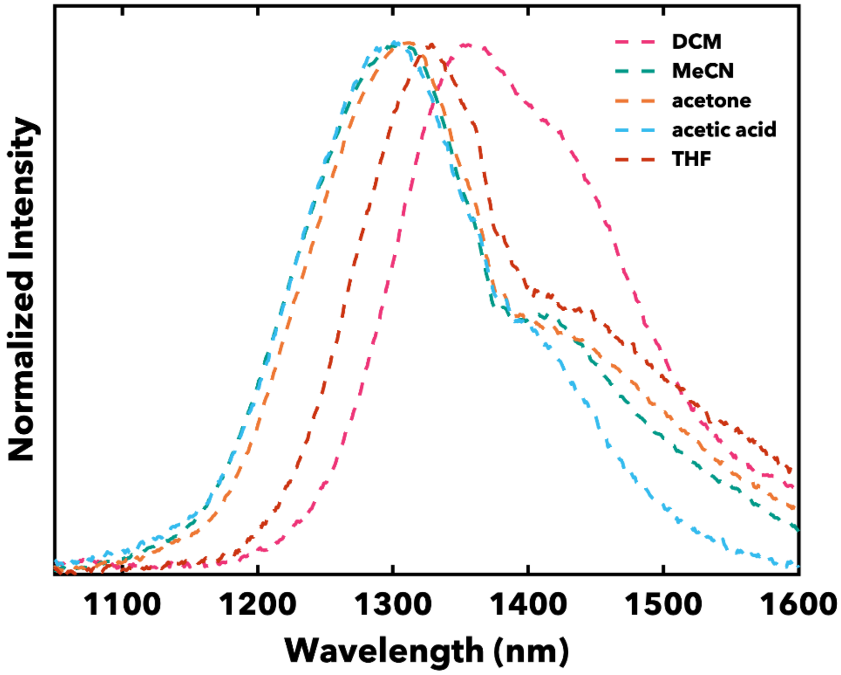

Figure S89. Solvatochromism of **2** across solvents at 298 K.

5. Photoluminescence quantum yield measurements

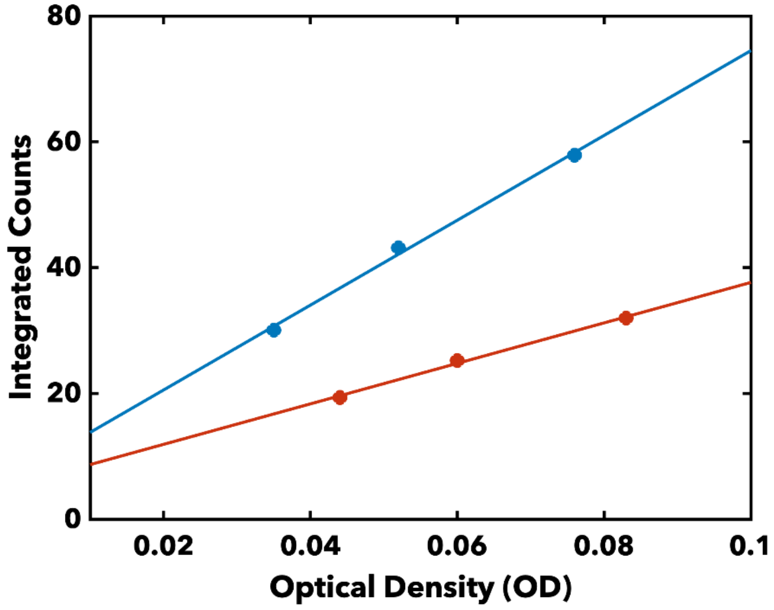

Figure S90. Photoluminescence quantum yield determination of **1** (red) in DCM at 298 K. **PtdppeTTFtt**[9] **(**blue) used as a reference (PLQY = 0.136% in DCM).

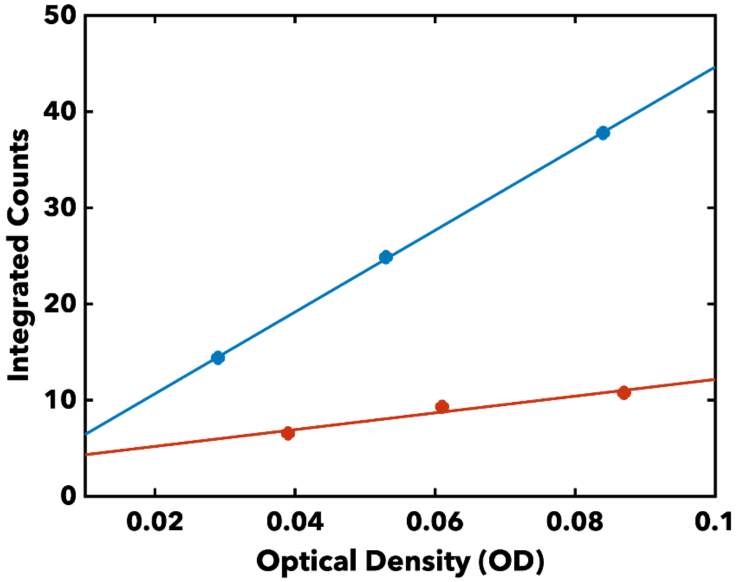

Figure S91. Photoluminescence quantum yield determination of **2** (red) in DCM at 298 K. **PtdppeTTFtt**[9] **(**blue) used as a reference (PLQY = 0.136% in DCM).

6. Molar absorption coefficient determination

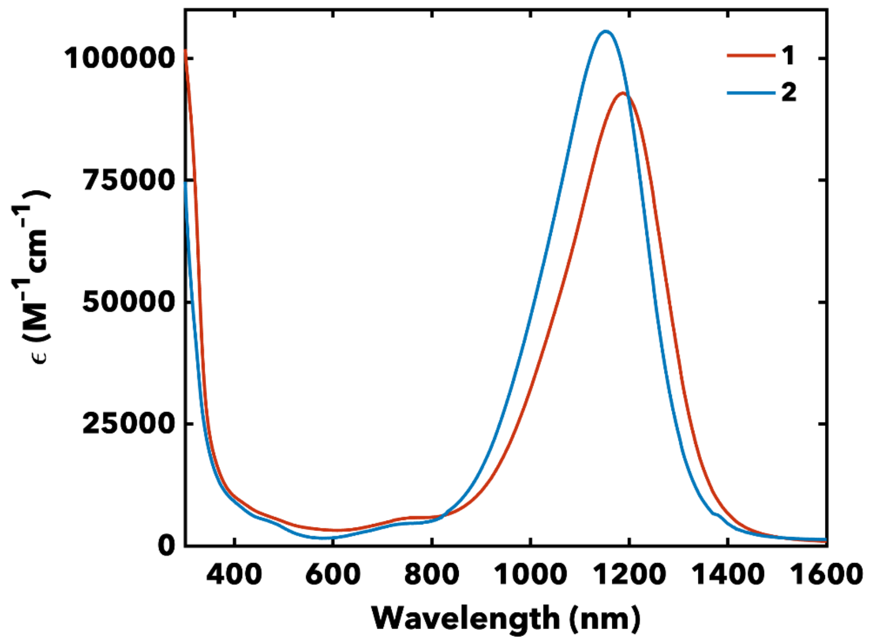

Figure S92. Absorption spectra of **1** and **2** in DCM at 298 K.

7. Variable wavelength excitation and oxygen experiments

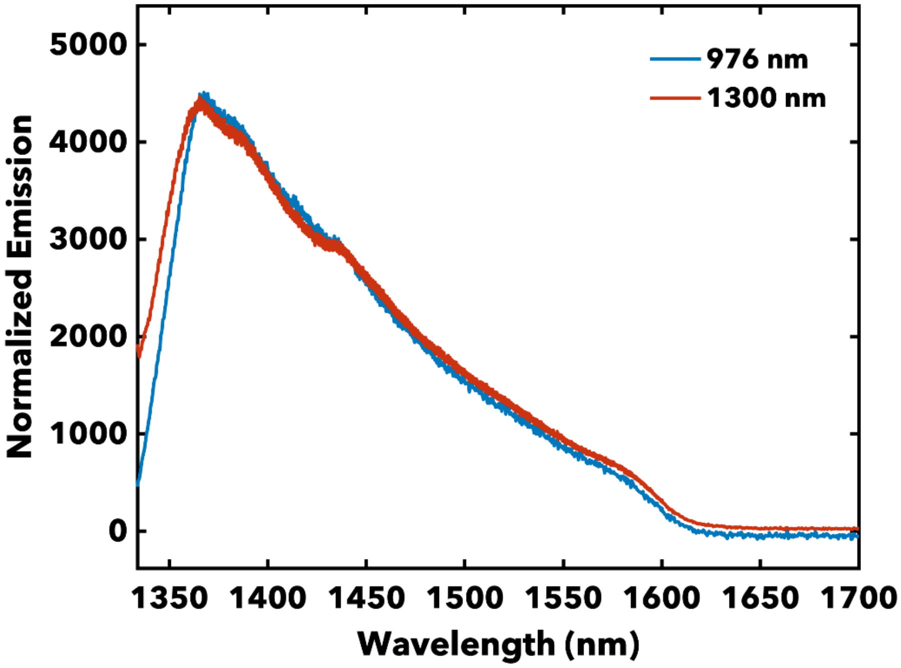

Figure S93. Photoluminescence spectra of **1** in anaerobic DCM at 298 K, at varying excitation wavelengths.

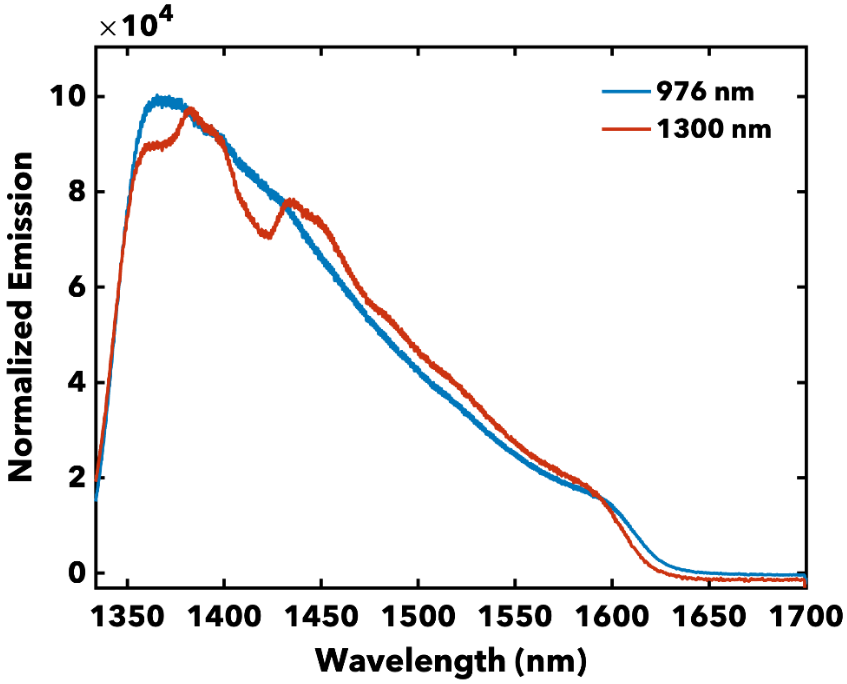

Figure S94. Photoluminescence spectra of **2** in anaerobic DCM at 298 K, at varying excitation wavelengths.

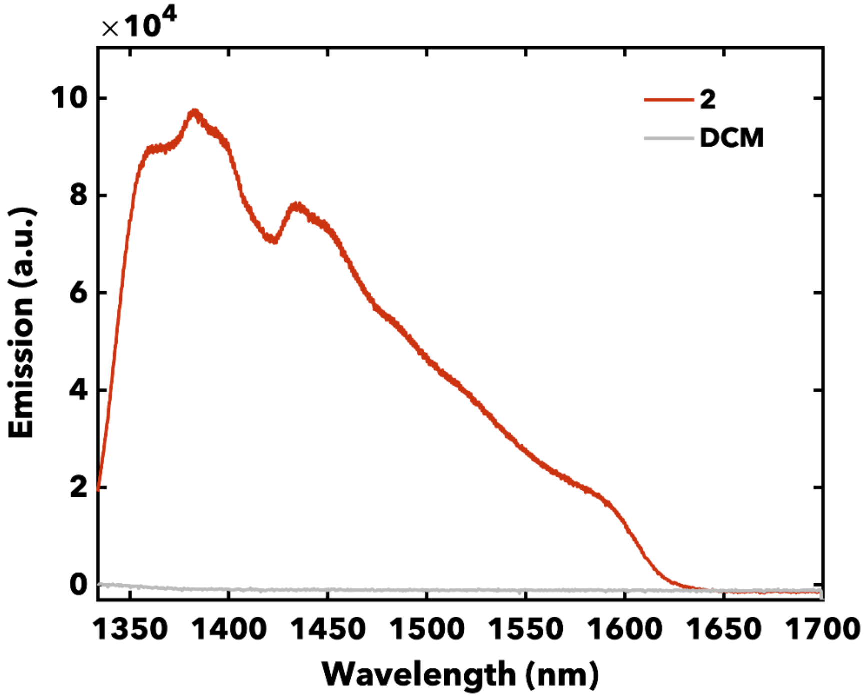

Figure S95. Photoluminescence spectra of **2** in anaerobic DCM and a DCM blank at 298 K, using 1300 nm light for excitation.

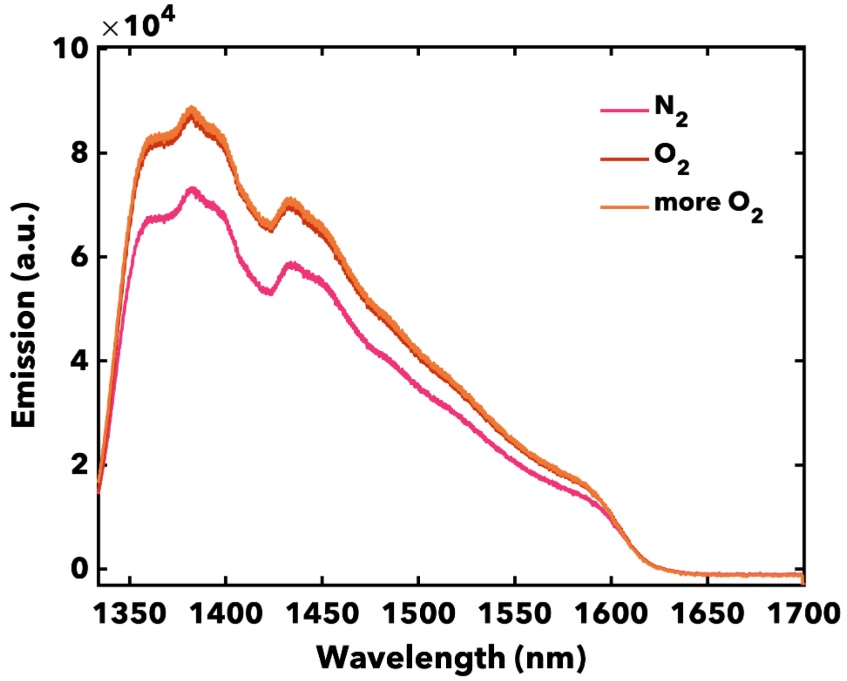

Figure S96. Photoluminescence spectra of **2** in anaerobic DCM and after increasing exposure to oxygen at 298 K, using 1300 nm light for excitation.

EPR spectroscopy

1. CW spectra

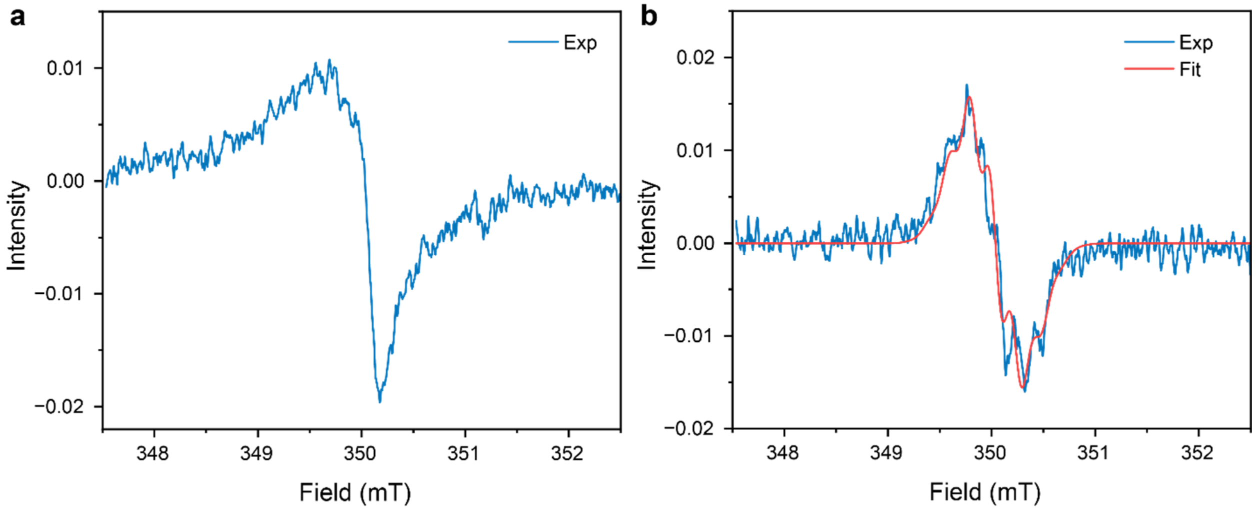

Figure S97. Experimental X-band CW EPR spectrum of a 1×10^{-4} mol/L 1:1 DCM:toluene solution of (**a**) **2** and (**b**) **2-TTFtt** at 295 K.

2. Pulsed EPR

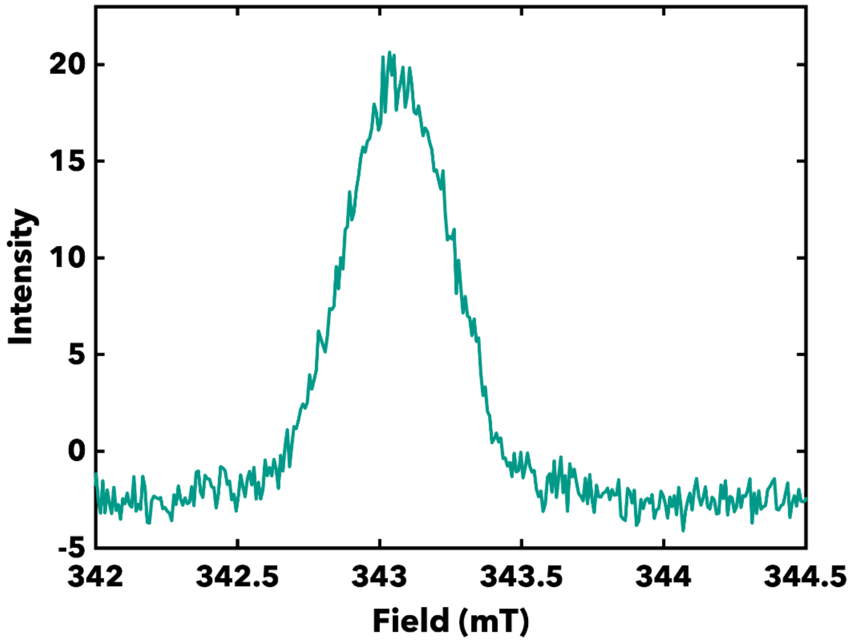

Figure S98. Experimental X-band EDFS spectrum of a 1×10^{-4} mol/L 1:1 DCM:toluene solution of **1** at 295 K.

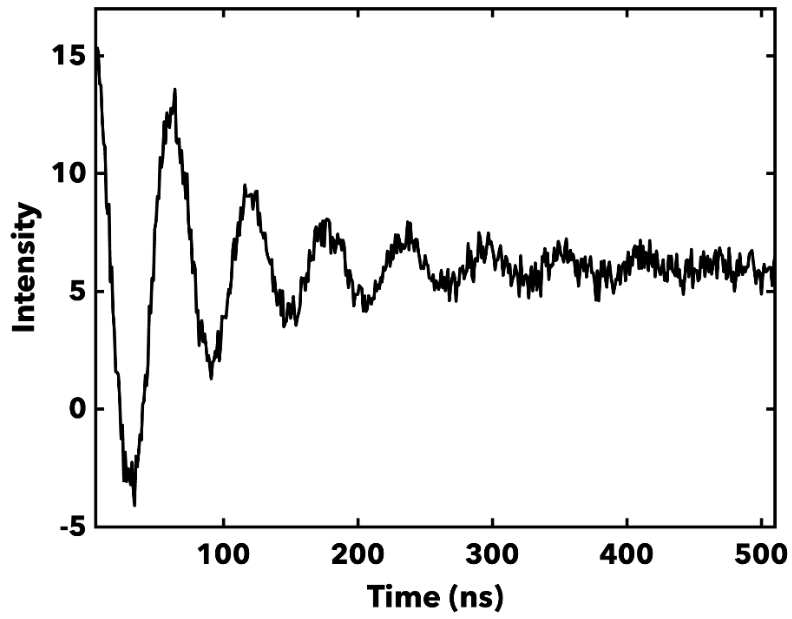

Figure S99. Rabi oscillations for a 1 × 10^{-4} mol/L 1:1 DCM:toluene solution of **1** at 295 K.

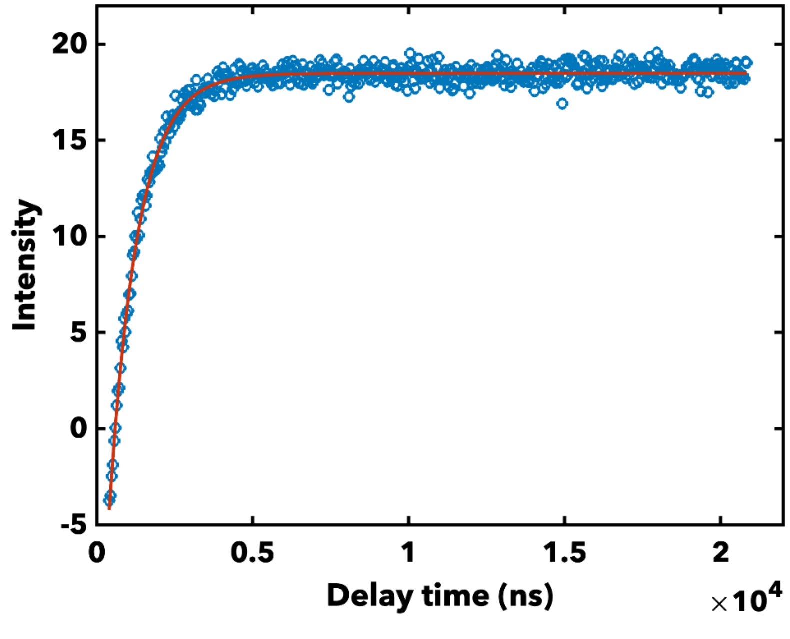

Figure S100. Spin−lattice relaxation time (T_1) of a 1 × 10^{-4} mol/L 1:1 DCM:toluene solution of **1** at 295 K. T_1 = 0.916 µs.

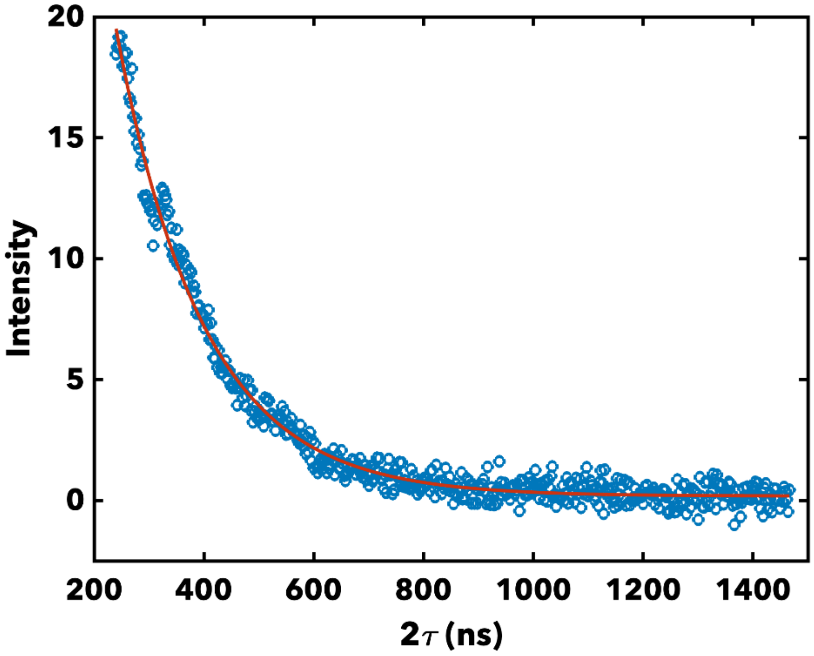

Figure S101. Phase memory time (T_m) of a 1 × 10^{-4} mol/L 1:1 DCM:toluene solution of **1** at 295 K. T_m = 158 ns.

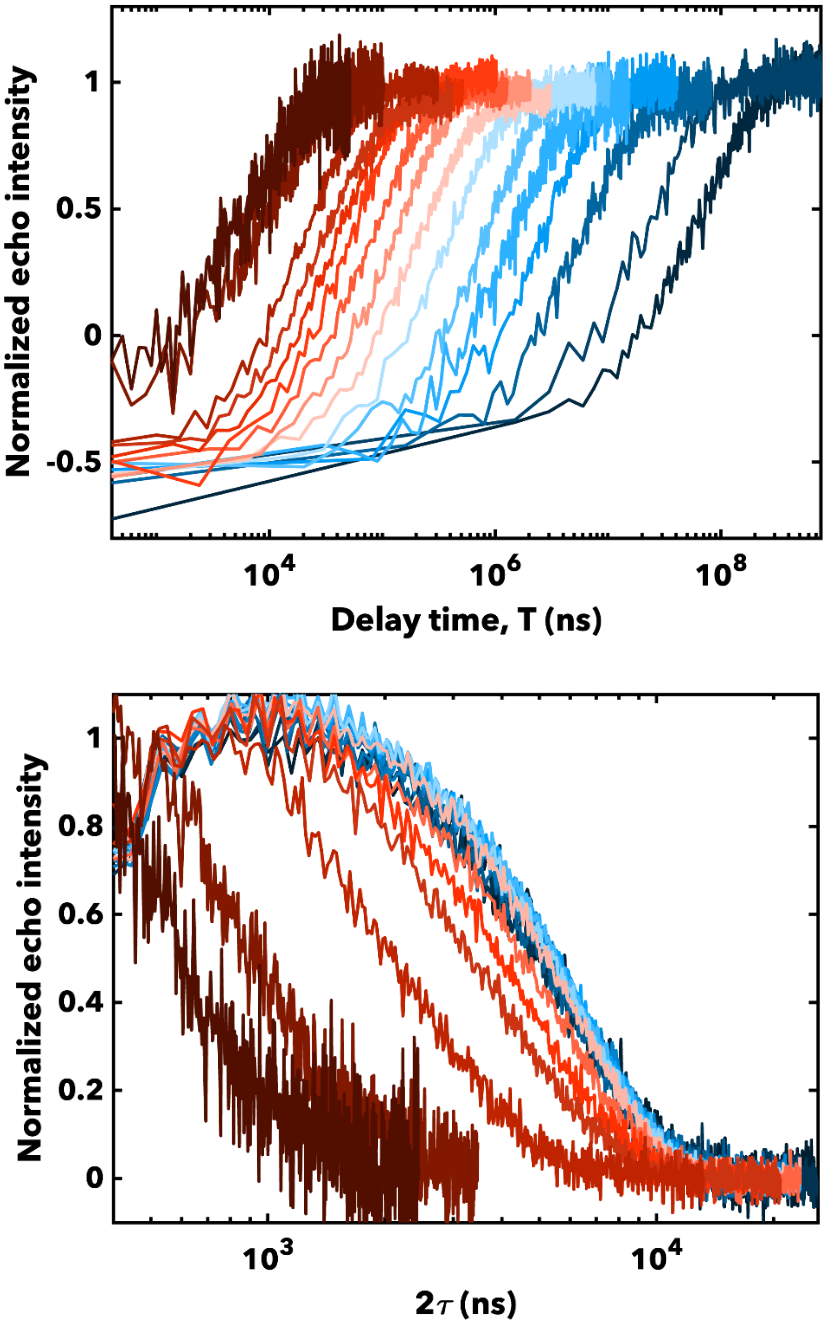

Figure S102. Measurement of (upper) spin−lattice relaxation times (T_1) and (lower) phase memory times (T_m) of 1×10^{-4} mol/L DCM:toluene solution of **2** across various temperatures. Normalized echo intensity shown as a function of delay time T and 2 τ from 153 K to 10 K. See methods for experimental and fitting details.

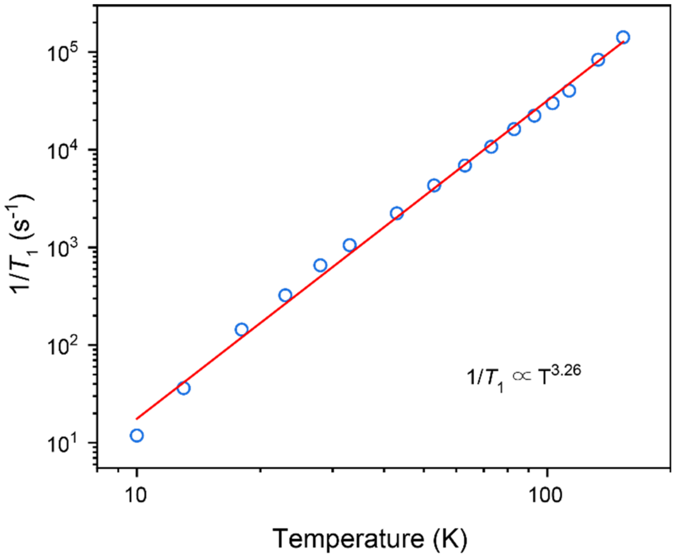

Figure S103. Variable-temperature inversion recovery experiments of **2**, showing the temperature dependence of T_1. The $1/T_1$ vs T data is fitted by the Raman spin−lattice process, $\frac{1}{T_1} = aT^m$, where a is a pre-factor and m is the exponent. Fitting revealed m = 3.26

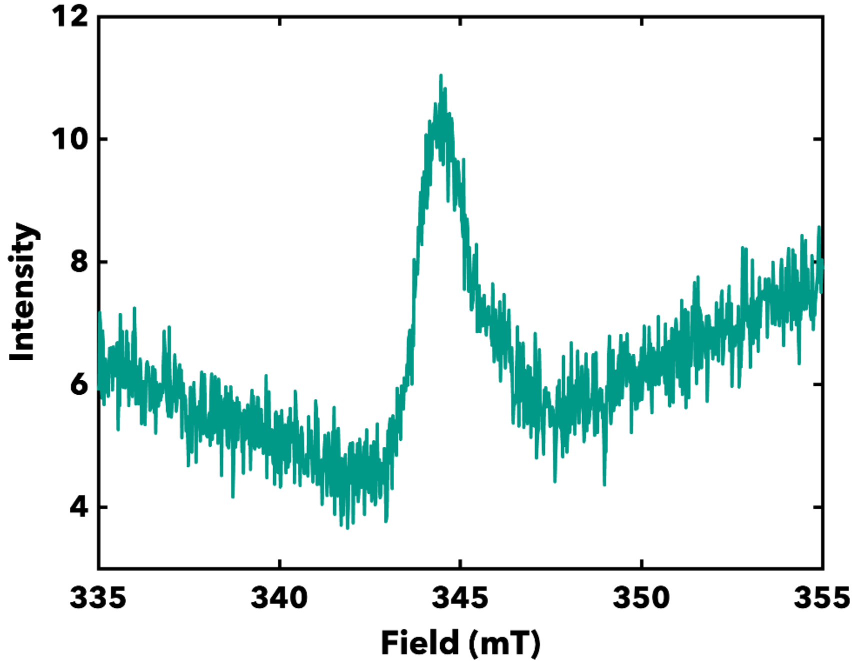

Figure S104. Experimental X-band EDFS spectrum of a 1 × 10^{-4} mol/L 1:1 DCM:toluene solution of **2-TTFtt** at 30 K.

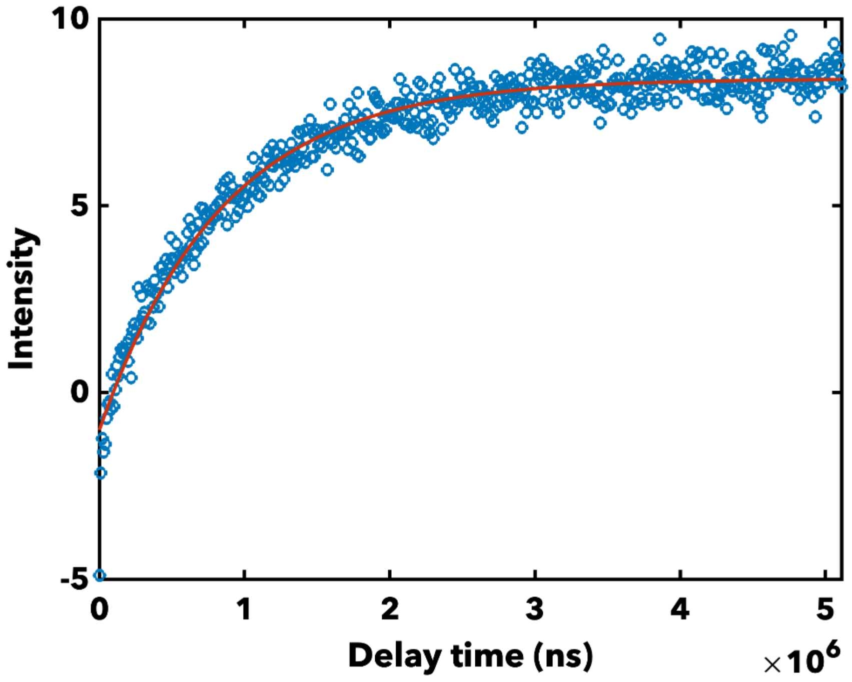

Figure S105. Spin−lattice relaxation time (T_1) of a 1 × 10^{-4} mol/L 1:1 DCM:toluene solution of **2-TTFtt** at 30 K. T_1 = 840.49 μs.

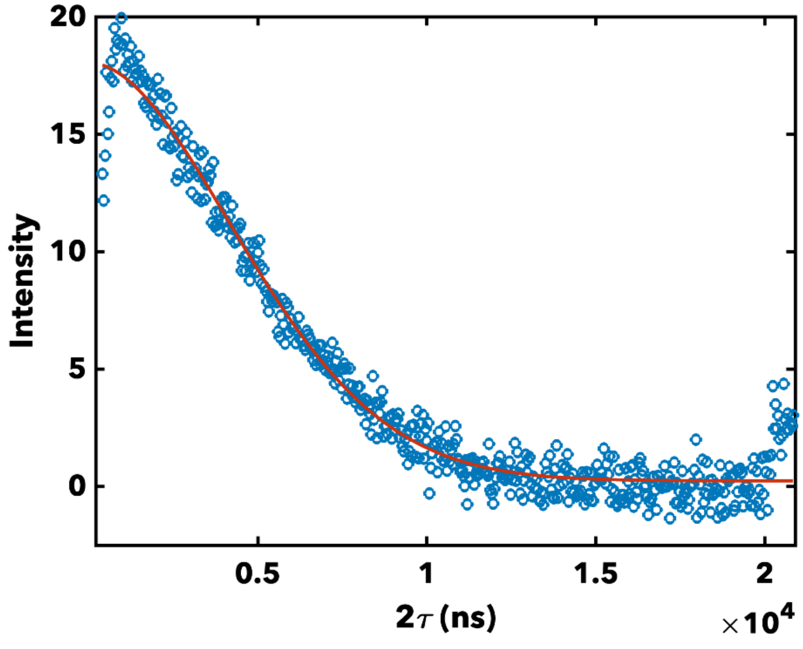

Figure S106. Phase memory time (T_m) of a 1 × 10^{-4} mol/L 1:1 DCM:toluene solution of **2-TTFtt** at 30 K. T_m = 6.112 μs.

X-ray crystallography

Compound	1
Empirical formula	$C_{146}H_{92}B_2Cl_8F_{48}P_4Pt_2S_4Se_4$
Formula weight	4021.55 g/mol
Temperature (K)	100(2)
Crystal system	triclinic
Space group	P-1
a (Å)	13.2573(6)
b (Å)	17.7601(9)
c (Å)	18.4355(9)
α (°)	66.493(2)
β (°)	70.047(2)
γ (°)	89.853(2)
Volume (Å^3)	3696.3(3)
Z	1
ρ_{calc}g (cm^3)	1.807
μ (mm^{-1})	3.232
F(000)	1962.0
Crystal size (mm^3)	0.184 x 0.16 x 0.04
Radiation	MoKα (λ = 0.71073)
2θ range for data collection (°)	3.84 to 56.45
Index ranges	-17≤ h ≤ 17, -23 ≤ k ≤ 23, -24 ≤ l ≤ 24
Reflections collected	202383
Independent reflections	18225 [R_{int} = 0.0452, R_{sigma} = 0.0251]
Data/restraints/parameters	18225/1140/1002
Goodness-of-fit on F^2	1.061
Final R indexes [I>=2σ (I)]	R_1 = 0.0480, wR_2 = 0.1015
Final R indexes [all data]	R_1 = 0.0645, wR_2 = 0.1160
Largest diff. peak/hole/e Å^{-3}	5.76/-3.83

Table S1. Crystallographic data for complex **1**.

Table S2. SXRD coordinates of **1**.

Pt	5.382111	6.773219	9.555791
Se	5.642416	8.165273	7.578666

S	4.514083	11.031387	7.088074
S	2.729557	11.014789	9.312457
P	7.024347	5.389404	8.757499
P	5.177333	5.711642	11.597439
C	3.365328	11.877364	8.001752
C	4.532742	9.600260	8.031580
C	3.680200	9.601091	9.116377
C	7.713225	5.894045	7.143020
C	6.923052	5.801705	5.995427
H	6.055987	5.417347	6.046511
C	7.401882	6.268182	4.781899
H	6.851847	6.220725	4.008694
C	8.672795	6.804913	4.687705
H	8.995585	7.116228	3.850056
C	9.470770	6.888024	5.811749
H	10.346402	7.250849	5.744385
C	9.000843	6.441006	7.044117
H	9.550771	6.507683	7.816301
C	6.418319	3.706626	8.428764
C	5.086648	3.391367	8.654828
H	4.497129	4.059087	8.984930
C	4.599803	2.112158	8.406785
H	3.684595	1.905623	8.561137
C	5.471259	1.138169	7.926397
H	5.153508	0.254442	7.778639
C	6.792514	1.438638	7.661085
H	7.374025	0.770726	7.317577
C	7.268970	2.718772	7.899709
H	8.174789	2.929153	7.704147
C	8.476009	5.362143	9.835389
C	8.920177	6.608978	10.295368
H	8.409383	7.387736	10.111973
C	10.101187	6.713828	11.017520
H	10.411687	7.565366	11.302330
C	10.826486	5.567830	11.320509
H	11.634321	5.636407	11.817569
C	10.381997	4.340497	10.906057
H	10.873850	3.560539	11.137350
C	9.211077	4.218038	10.146228
H	8.921875	3.365440	9.845813
C	3.584832	4.860910	11.818166
C	2.563127	5.018581	10.882509
H	2.716262	5.540288	10.103433
C	1.330959	4.426108	11.074036
H	0.641685	4.545127	10.432263
C	1.103230	3.663532	12.201220

H	0.247561	3.276217	12.343562
C	2.115811	3.455591	13.124318
H	1.960533	2.907015	13.884398
C	3.350482	4.045671	12.939070
H	4.044424	3.899899	13.571597
C	6.405353	4.491959	12.183951
C	7.454645	4.891439	12.989307
H	7.553817	5.807814	13.221322
C	8.368157	3.951513	13.463415
H	9.093366	4.227252	14.009896
C	8.218987	2.616816	13.138447
H	8.834142	1.977428	13.476634
C	7.180485	2.205829	12.323672
H	7.095162	1.289743	12.090715
C	6.255177	3.142821	11.843284
H	5.534341	2.863493	11.291215
C	5.243736	7.017269	12.890404
C	4.555943	6.907960	14.092943
H	4.042748	6.130988	14.277500
C	4.616891	7.938436	15.028600
H	4.152642	7.855171	15.853658
C	5.346936	9.081155	14.767998
H	5.380268	9.782720	15.408059
C	6.029236	9.200194	13.574878
H	6.529422	9.987261	13.392236
C	5.985532	8.173270	12.639221
H	6.462667	8.258307	11.822248
Se	3.536253	8.187738	10.292856
C	1.551979	8.529497	13.306426
H	1.710469	9.218954	13.998216
H	2.427774	8.262914	12.928348
Cl	0.540904	9.179979	12.045801
Cl	0.773859	7.144896	14.022298
Cl	3.774736	1.614162	16.050601
Cl	6.556238	0.511094	15.716214
C	4.905583	0.630576	15.151052
H	4.931471	0.976710	14.223778
H	4.536731	-0.286681	15.108445
F	4.524296	13.782060	-0.029828
F	5.928058	12.842861	-1.324991
F	5.915515	14.971686	-1.174281
F	10.484443	15.457037	3.094262
F	10.676042	15.355225	0.949786
F	9.395440	16.820547	1.843056
F	7.364615	10.283362	9.709798
F	8.868294	9.428602	8.431903

F	9.329384	11.131168	9.657991
F	1.364420	12.590470	4.595081
F	1.424437	12.230369	2.494562
F	2.665980	13.759971	3.339166
F	7.513431	7.630823	0.313979
F	9.567501	7.064532	0.139721
F	8.847588	8.898641	-0.731571
F	12.951543	9.423581	2.854068
F	12.226673	8.542188	4.639038
F	12.330509	10.665001	4.480479
C	7.398578	12.686945	2.791272
C	6.584418	12.791007	1.642110
H	5.877859	12.166439	1.526204
C	6.771171	13.767375	0.679764
C	5.791586	13.851401	-0.455269
C	7.807250	14.678204	0.781807
H	7.932858	15.353106	0.124477
C	8.657714	14.573017	1.879164
C	9.796816	15.545849	1.946669
C	8.458000	13.606496	2.858778
H	9.055089	13.566631	3.595357
C	7.462132	11.977361	5.430265
C	7.164786	13.289709	5.829018
H	6.892551	13.916809	5.169160
C	7.253714	13.713290	7.157149
C	6.948683	15.130423	7.555902
C	7.656567	12.825030	8.144613
H	7.723801	13.105202	9.049955
C	7.959631	11.519326	7.786677
C	8.377744	10.589031	8.885603
C	7.861523	11.106709	6.455406
H	8.073039	10.206094	6.236704
C	5.660275	10.931075	3.860371
C	5.265976	9.597193	3.996951
H	5.929784	8.925367	4.098492
C	3.923547	9.233729	3.990672
C	3.510505	7.800699	4.124113
C	2.915021	10.160790	3.846242
H	2.003140	9.895758	3.830778
C	3.277156	11.498458	3.725360
C	2.196119	12.522308	3.544822
C	4.616735	11.868100	3.741059
H	4.836258	12.789800	3.669330
C	8.316283	10.381318	3.364284
C	8.066965	9.594369	2.227680
H	7.187408	9.568934	1.868912

C	9.065378	8.853874	1.612282
C	8.759439	8.111544	0.346947
C	10.359257	8.823919	2.106798
H	11.037241	8.303223	1.691436
C	10.623809	9.592375	3.241832
C	12.015689	9.561577	3.799145
C	9.640195	10.333796	3.847811
H	9.859607	10.836279	4.624501
B	7.209550	11.473840	3.885489
F	7.971477	15.675023	8.230957
F	5.912004	15.196714	8.408355
F	7.326471	15.963319	6.571578
F	4.053488	7.018491	5.053490
F	4.185157	7.105346	3.175897
F	2.261420	7.526311	3.753618
H	2.363026	8.080491	12.957627
H	1.809389	9.436185	13.608081
Cl	0.362268	8.655825	12.030102
Cl	0.921729	7.623200	14.658105
Cl	5.726792	-0.131412	15.097675
C	5.434290	1.546485	15.499568
H	5.562408	2.122476	14.705045
H	6.051507	1.846710	16.213431
F	6.452373	15.868533	6.551170
F	2.838649	7.640771	5.290544
F	4.495856	6.930627	4.303081
F	2.611151	7.407657	3.226133

Computational methodology

Computational Analysis and Electronic Structure

Geometry optimizations (ground and TD-DFT excited state) and frequency calculations were carried out at the PBE0-D3BJ/def2-SVP level of theory as implemented in Gaussian 16 Rev.A.03. CASSCF and TD-DFT calculations were carried out as implemented in ORCA version 5.0.4. CASSCF calculations utilized the def2-SVP basis set in combination with RIJK integral fitting and the def2/JK auxiliary basis set. A minimal [4e,4o] active space was chosen, shown previously to accurately capture the biradical character arising from the near-degenerate frontier pi orbitals in TTFtt-based system. TD-DFT calculations utilized the def2-TZVP basis set, in combination with RIJCOSX integral fitting and the def2/J auxiliary basis set. DCM solvation was modeled using the implicit CPCM model. Orbitals associated with electronic transitions shown below are NTOs as discussed in the main text unless otherwise noted. The diradical character is calculated directly from the occupations of the natural orbitals. The reported value $N_{diradical}$ is simply the deviation from the full/empty occupancies of the frontier orbitals of a closed-shell singlet, i.e. y = (2-N(HONO)+N(LUNO)). In this case, a value of 2 would correspond to a full diradical (N(HONO) = N(LUNO) = 1) with a maximum of 2 unpaired electrons.

Table S3. Triplet-singlet gaps for **1** and **2**.

	dG(T1-S0) (kcal/mol)	S^2 (S)	dE(H-L) a.u.	dG(T2-S1) (kcal/mol)
**1**	2.5532975	0	0.04748	2.32911
**2**	1.745705	0	0.04654	2.25993

Table S4. Predicted diradical character for **1** and **2**.

	CASSCF(4,4)		
	**N(HONO)**	**N(LUNO)**	**$N_{diradical}$**
**1**	1.63274	0.36876	0.736
**2**	1.64163	0.35837	0.71674

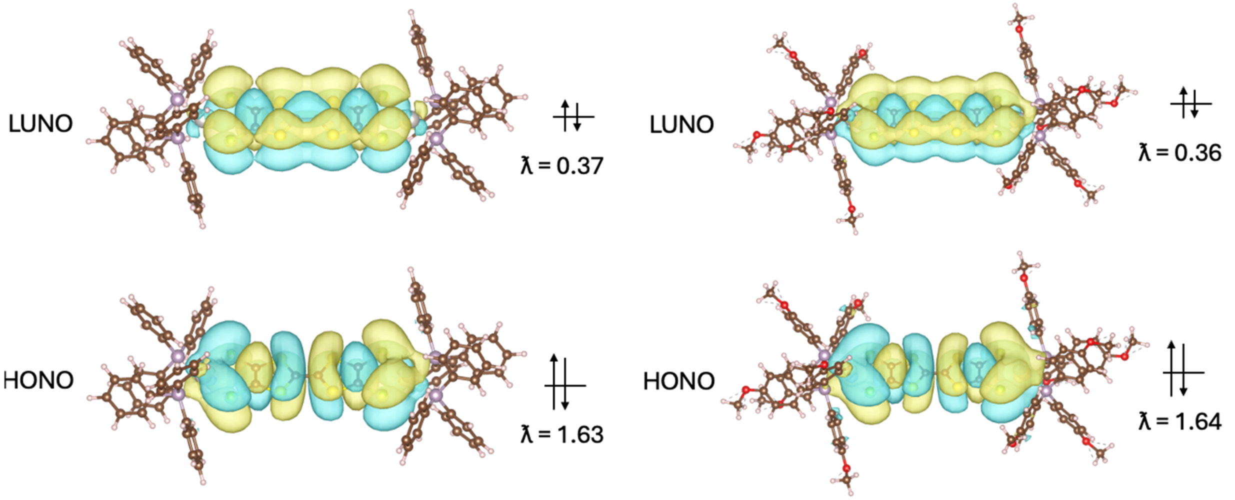

Figure S107. HONO-LUNO diagrams for **1** and **2**, showing multireference character.

Predicted Photophysical Properties

Table S5. Predicted absorption and emission for singlet and triplet states for **1** and **2**, along with calculated oscillator strengths.

	**Abs$_S$ (nm)**	***f***	**Abs$_T$ (nm)**	***f***	**Emiss$_S$ (nm)**	***f***
**1**	1111.4	1.0317	1386.8	0.7821	1213.6	0.9664
**2**	1138.4	1.0512	1391.4	0.7989	1245.7	0.9839

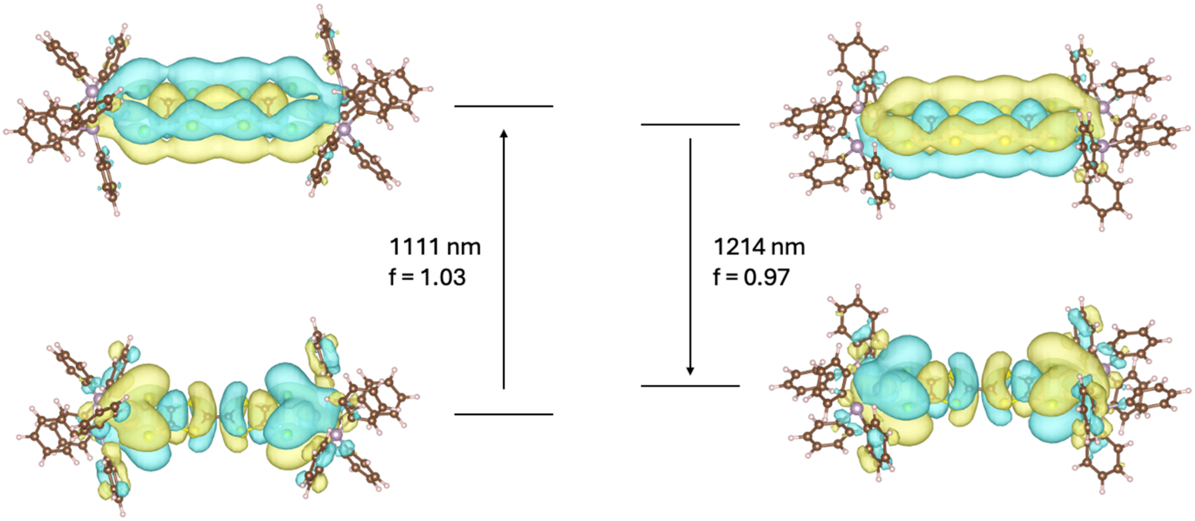

Figure S108. Calculated singlet absorption and emission for **1**.

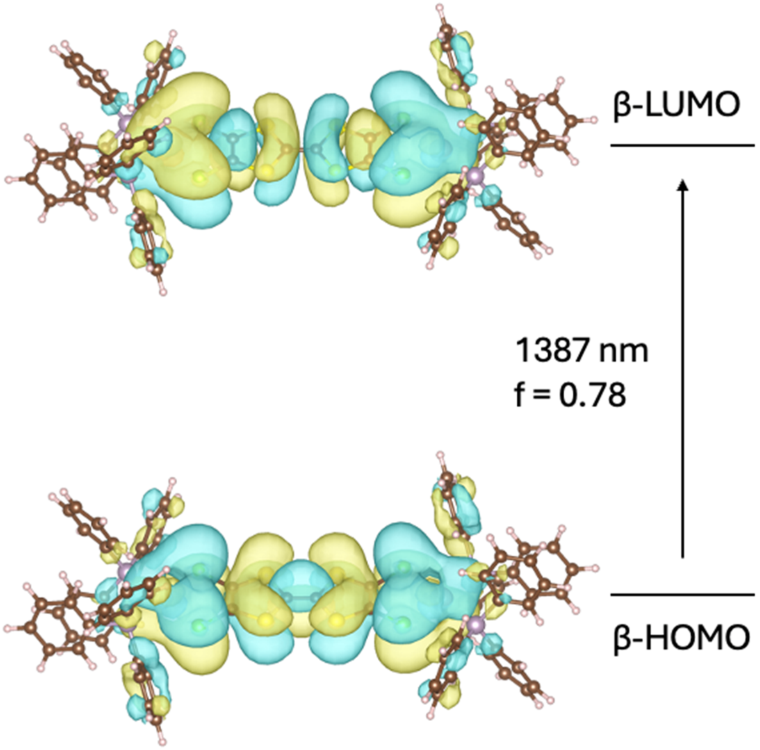

Figure S109. Calculated triplet absorption for **1**.

Table S6. Calculated vibrational frequencies (in cm^{-1}) and intensities for **1** and its corresponding TTFtt analog.[11] Cells with no values were either not reported or a vibration at that frequency was not predicted. Green cells indicate core vibrations.

	**1**			**1-TTFtt**[11]	
**Freq.**	**Intensity**	**Type**	**Freq.**	**Intensity**	**Type**
1348.72	13082	C=C TTF	1346	15099	C=C TTF
935	96	S-C-Se TTF	995	286	S-C-S TTF
513	38	S-C-S TTF	--	--	--
339	193	Pt-Se-TTF	--	--	--
3246	4.6	Ph C-H	3241	5.3	Ph C-H
--	--	--	1654	7.7	Ph C=C
1664	25	Ph C=C	1671	3.3	Ph C=C
1480	78	Ph C=C	--	--	--
--	--	--	1390	1.1	Ph C=C
--	--	--	1394	3.8	Ph C=C
1325	23	Ph C=C/H	1396	4.7	Ph C=C
1135	234	P-Ph	1126	115	C=C Ph/C-P
721	153	Ph-H	--	--	--
552	927	Pt-P-Ph	--	--	--
509	128	P-Ph	--	--	--

References

(1) Decker, C.; Henderson, W.; Nicholson, B. K. Triphenyl-Phosphine and -Arsine Analogues Which Facilitate the Electrospray Mass Spectrometric Analysis of Neutral Metal Complexes. *J. Chem. Soc., Dalton Trans.* **1999**, No. 19, 3507–3513. https://doi.org/10.1039/A906010C.

(2) Fawcett, J.; Henderson, W.; Kemmitt, R. D. W.; Russell, D. R.; Upreti, A. Synthesis and Electrospray Mass Spectrometry of Platinum(II) Complexes of 5,5-Diethylbarbituric Acid (Hdebarb); Crystal Structure of Cis-[PtCl(Debarb)(PPh3)2]·CH2Cl2. *J. Chem. Soc., Dalton Trans.* **1996**, No. 9, 1897–1903. https://doi.org/10.1039/DT9960001897.

(3) McNamara, L. E.; Boyn, J.-N.; Anferov, S. W.; Filatov, A. S.; Maloney, M. W.; Mazziotti, D. A.; Schaller, R. D.; Anderson, J. S. Variable Peripheral Ligand Donation Tunes Electronic Structure and NIR II Emission in Tetrathiafulvalene Tetrathiolate Diradicaloids. *J. Am. Chem. Soc.* **2024**, *146* (25), 17285–17295. https://doi.org/10.1021/jacs.4c04032.

(4) Xie, J.; Boyn, J.-N.; Filatov, A. S.; McNeece, A. J.; Mazziotti, D. A.; Anderson, J. S. Redox, Transmetalation, and Stacking Properties of Tetrathiafulvalene-2,3,6,7-Tetrathiolate Bridged Tin, Nickel, and Palladium Compounds. *Chem. Sci.* **2020**, *11* (4), 1066–1078. https://doi.org/10.1039/C9SC04381K.

(5) Sheldrick, G. M. Crystal Structure Refinement with SHELXL. *Acta Cryst C* **2015**, *71* (1), 3–8. https://doi.org/10.1107/S2053229614024218.

(6) Sheldrick, G. M. SHELXT – Integrated Space-Group and Crystal-Structure Determination. *Acta Cryst A* **2015**, *71* (1), 3–8. https://doi.org/10.1107/S2053273314026370.

(7) Dolomanov, O. V.; Bourhis, L. J.; Gildea, R. J.; Howard, J. a. K.; Puschmann, H. OLEX2: A Complete Structure Solution, Refinement and Analysis Program. *J Appl Cryst* **2009**, *42* (2), 339–341. https://doi.org/10.1107/S0021889808042726.

(8) Bain, G. A.; Berry, J. F. Diamagnetic Corrections and Pascal's Constants. *J. Chem. Educ.* **2008**, *85* (4), 532. https://doi.org/10.1021/ed085p532.

(9) McNamara, L. E.; Boyn, J.-N.; Melnychuk, C.; Anferov, S. W.; Mazziotti, D. A.; Schaller, R. D.; Anderson, J. S. Bright, Modular, and Switchable Near-Infrared II Emission from Compact Tetrathiafulvalene-Based Diradicaloid Complexes. *J. Am. Chem. Soc.* **2022**, *144* (36), 16447–16455. https://doi.org/10.1021/jacs.2c04976.

(10) Stoll, S.; Schweiger, A. EasySpin, a Comprehensive Software Package for Spectral Simulation and Analysis in EPR. *Journal of Magnetic Resonance* **2006**, *178* (1), 42–55. https://doi.org/10.1016/j.jmr.2005.08.013.

(11) McNamara, L. E.; Melnychuk, C.; Boyn, J.-N.; Anferov, S. W.; Mazziotti, D. A.; Schaller, R. D.; Anderson, J. S. Elucidating Non-Radiative Decay in near-Infrared Lumiphores: Leveraging New Design Principles to Develop a Telecom Band Organic Dye Laser. *Chem* **2024**, *10* (7), 2266–2282. https://doi.org/10.1016/j.chempr.2024.03.023.
