# Supplementary figures and images for: Above the Energy Gap Law: Heavy Chalcogenide Substitution in NIR II-Emissive Diradicaloid Qubits
Source: ACS Cent Sci. 2025 Oct 29;11(11):2257–67. doi: 10.1021/acscentsci.5c01001 (PMC12670300; doi:10.1021/acscentsci.5c01001)

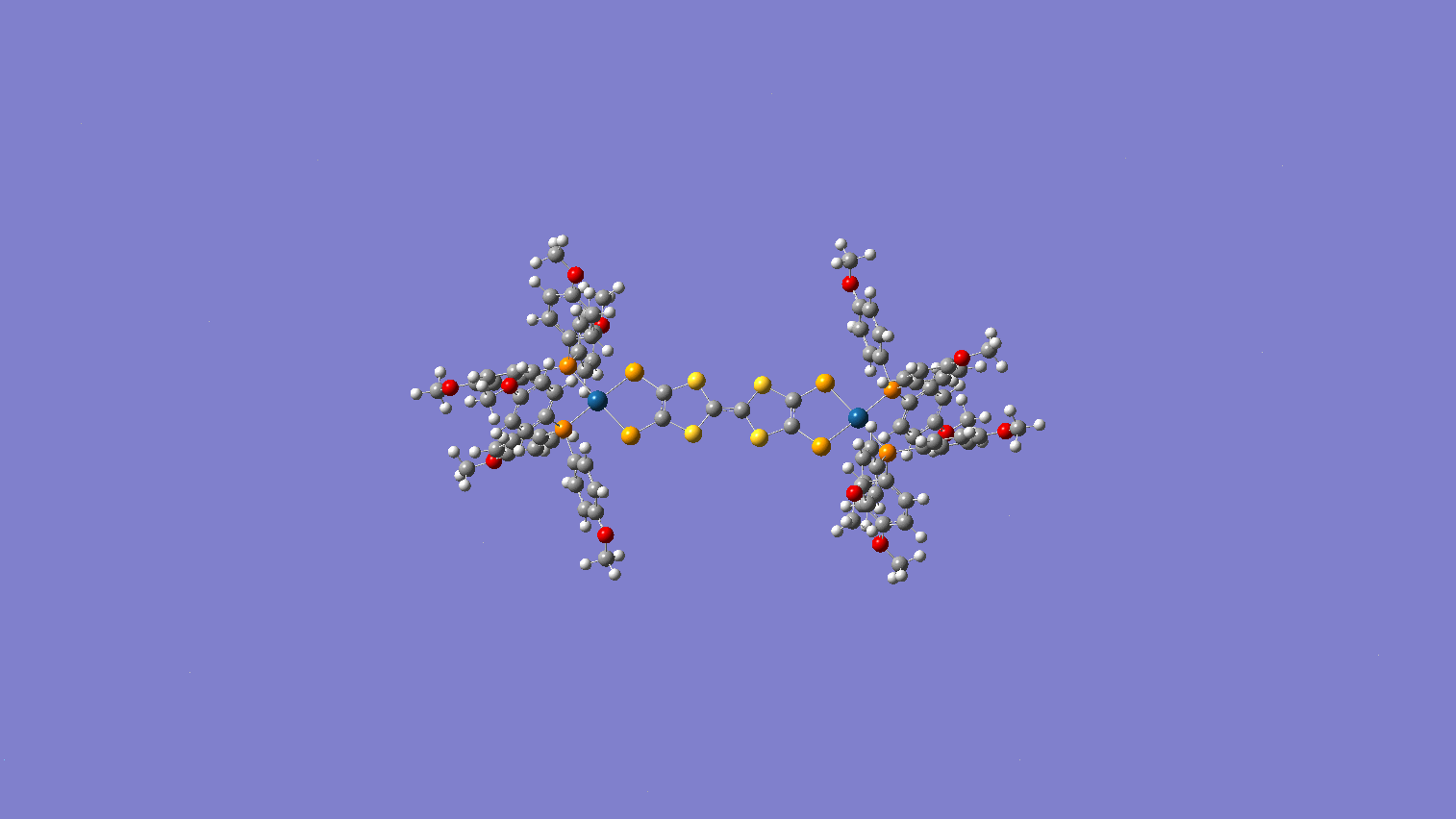

Supplement: Supplementary file 3 [file oc5c01001_si_003.zip › SuppVideo4.gif]

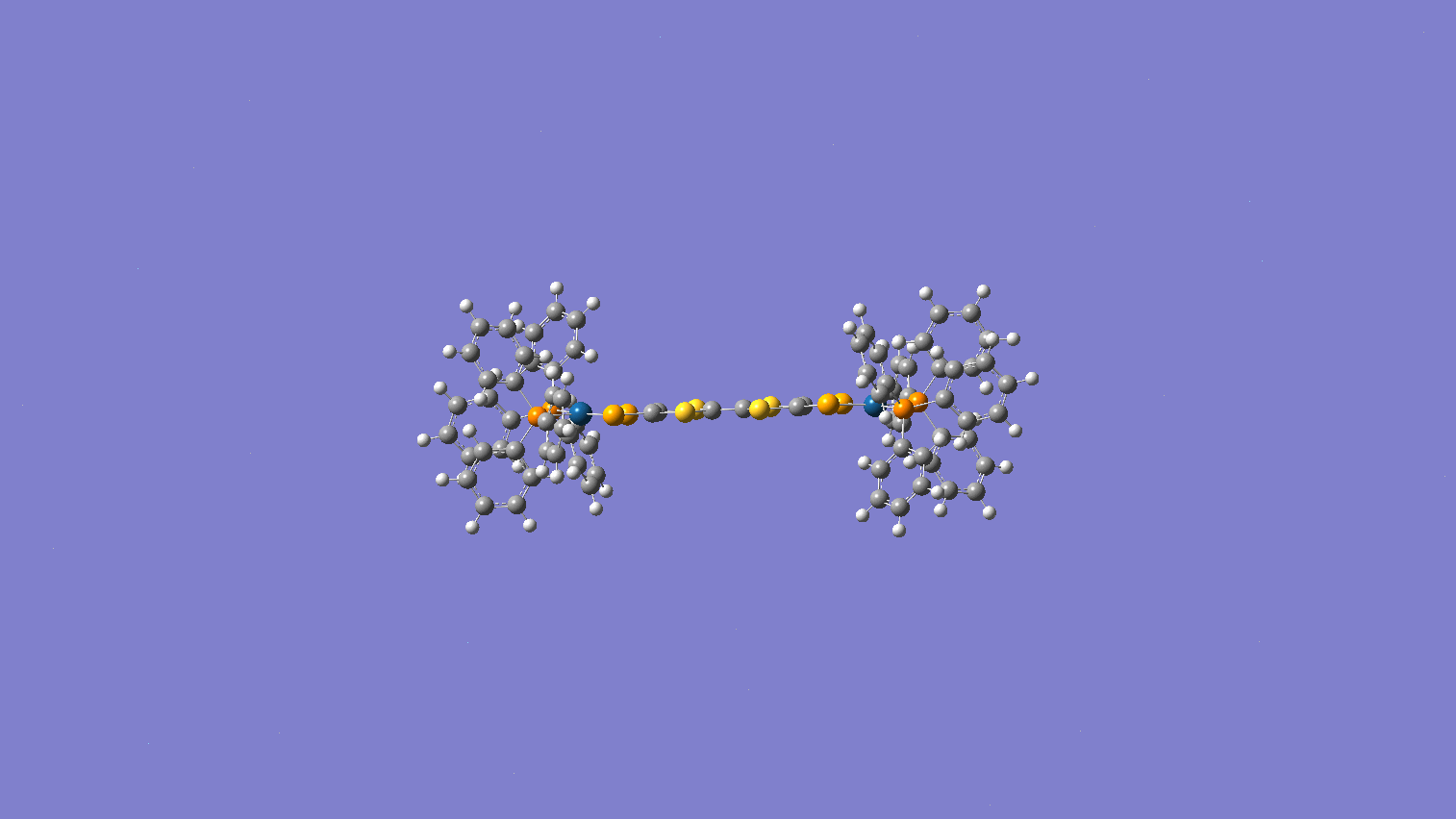

Supplement: Supplementary file 3 [file oc5c01001_si_003.zip › SuppVideo1.gif]

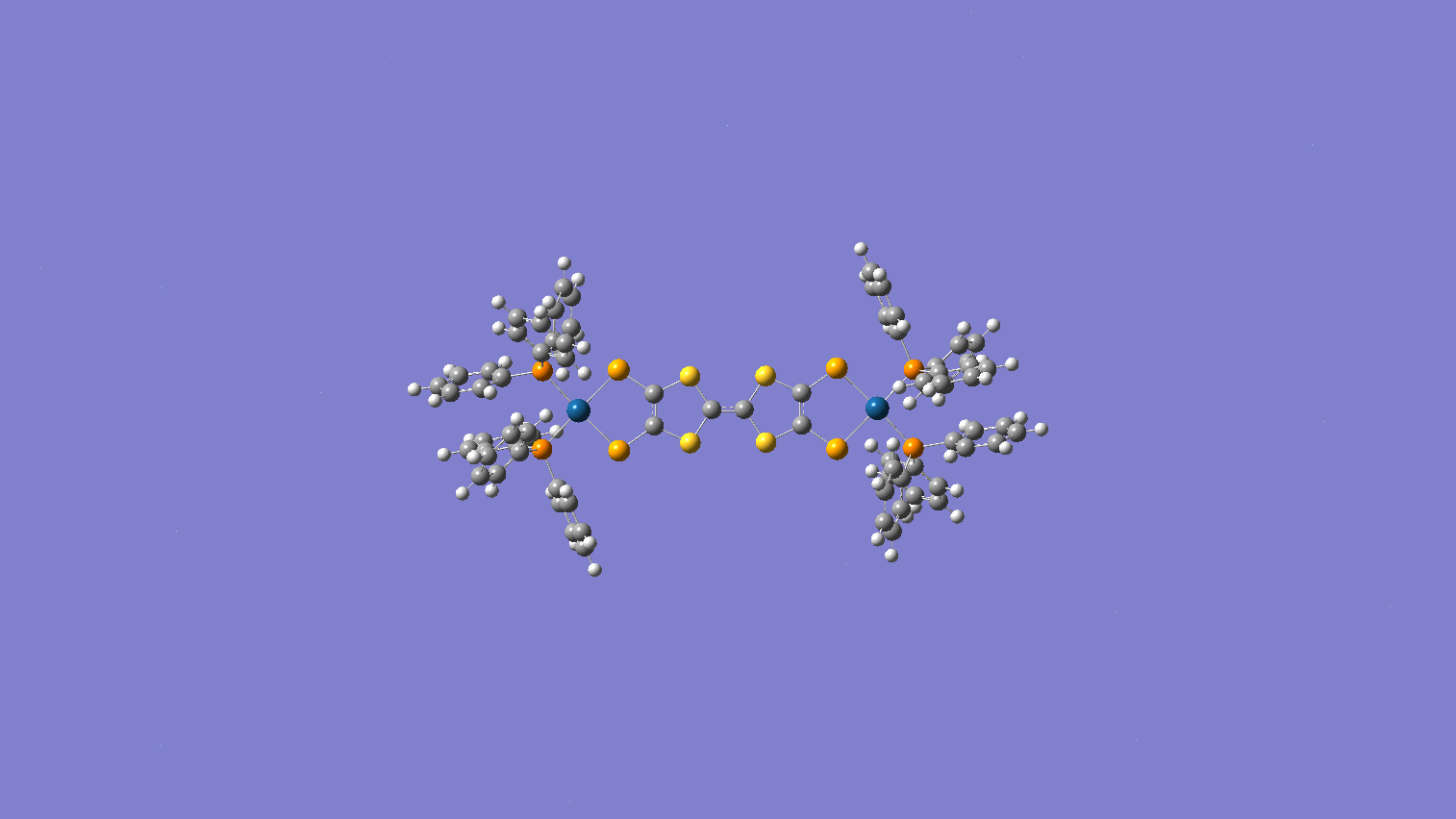

Supplement: Supplementary file 3 [file oc5c01001_si_003.zip › SuppVideo2.gif]

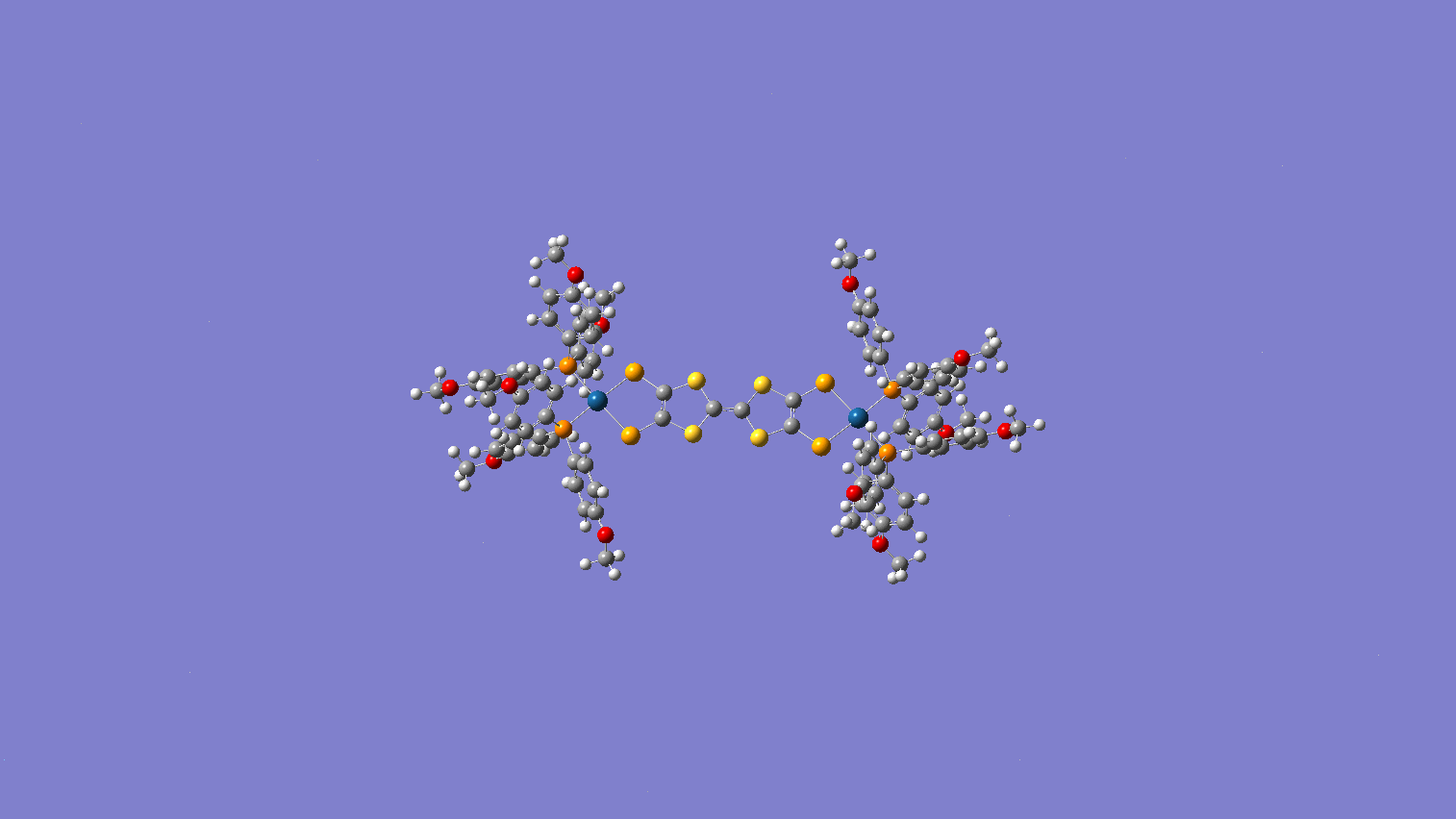

Supplement: Supplementary file 3 [file oc5c01001_si_003.zip › SuppVideo3.gif]
